# The Application of DNA Barcodes for the Identification of Marine Crustaceans from the North Sea and Adjacent Regions

**DOI:** 10.1371/journal.pone.0139421

**Published:** 2015-09-29

**Authors:** Michael J. Raupach, Andrea Barco, Dirk Steinke, Jan Beermann, Silke Laakmann, Inga Mohrbeck, Hermann Neumann, Terue C. Kihara, Karin Pointner, Adriana Radulovici, Alexandra Segelken-Voigt, Christina Wesse, Thomas Knebelsberger

**Affiliations:** 1 German Center of Marine Biodiversity (DZMB), Senckenberg am Meer, Wilhelmshaven, Niedersachsen, Germany; 2 Biodiversity Institute of Ontario, University of Guelph, Guelph, Ontario, Canada; 3 Alfred Wegener Institute Helmholtz Centre for Polar and Marine Research, Biologische Anstalt Helgoland, Helgoland, Schleswig-Holstein, Germany; 4 Department for Marine Research, Senckenberg am Meer, Wilhelmshaven, Niedersachsen, Germany; 5 Animal Biodiversity and Evolutionary Biology, Institute for Biology and Environmental Sciences, V. School of Mathematics and Science, Carl von Ossietzky University Oldenburg, Oldenburg, Niedersachsen, Germany; Tuscia University, ITALY

## Abstract

During the last years DNA barcoding has become a popular method of choice for molecular specimen identification. Here we present a comprehensive DNA barcode library of various crustacean taxa found in the North Sea, one of the most extensively studied marine regions of the world. Our data set includes 1,332 barcodes covering 205 species, including taxa of the Amphipoda, Copepoda, Decapoda, Isopoda, Thecostraca, and others. This dataset represents the most extensive DNA barcode library of the Crustacea in terms of species number to date. By using the Barcode of Life Data Systems (BOLD), unique BINs were identified for 198 (96.6%) of the analyzed species. Six species were characterized by two BINs (2.9%), and three BINs were found for the amphipod species *Gammarus salinus* Spooner, 1947 (0.4%). Intraspecific distances with values higher than 2.2% were revealed for 13 species (6.3%). Exceptionally high distances of up to 14.87% between two distinct but monophyletic clusters were found for the parasitic copepod *Caligus elongatus* Nordmann, 1832, supporting the results of previous studies that indicated the existence of an overlooked sea louse species. In contrast to these high distances, haplotype-sharing was observed for two decapod spider crab species, *Macropodia parva* Van Noort & Adema, 1985 and *Macropodia rostrata* (Linnaeus, 1761), underlining the need for a taxonomic revision of both species. Summarizing the results, our study confirms the application of DNA barcodes as highly effective identification system for the analyzed marine crustaceans of the North Sea and represents an important milestone for modern biodiversity assessment studies using barcode sequences.

## Introduction

In recent years, the use of molecular methods for specimen identification and classification has become quite popular, including proteome [1–3] or spectroscopic data [4,5]. However, the analysis of DNA sequence data represents the most used and accepted application to date. In most animals, mitochondrial DNA (mtDNA) exhibits several characteristics that make it highly attractive for molecular specimen identification, such as an almost exclusively maternal inheritance, a high number of copies within the mitochondria, the absence of introns, typically high substitution rates, and the absence of recombination [6–8]. Furthermore, as a consequence of uniparental inheritance and haploidy, mtDNA has a four-fold smaller effective population size compared to nuclear DNA, resulting in faster lineage sorting [9]. In this context, the standardized use of an approx. 650 base pair (bp) fragment of the cytochrome *c* oxidase subunit 1 (CO1) as DNA barcode represents a very successful mtDNA-based approach for the identification of animal specimens [10–12]. The idea of DNA barcoding is based on the assumption that each species will have similar DNA barcodes representing its intraspecific variability. In addition, the variation between species needs to exceed the variation within species, which allows a clear genetic delineation of species by so-called barcoding gaps [10,11,13]. The two main goals of DNA barcoding are (i) to assign unknown specimens to already described and classified species, and (ii) to enhance the discovery of new species and facilitate identification, particularly in cryptic, microscopic, and other organisms with complex or inaccessible morphology [10,11]. Whereas various phenomena may affect the application of DNA barcodes or mitochondrial DNA in general for successful specimen identification, e.g. heteroplasmy [14,15], incomplete lineage sorting [16], the presence of mitochondrial pseudogenes (numts) [17,18] or introgressive hybridization [19,20], DNA barcoding has become an important tool in numerous biological disciplines, e.g. modern biodiversity assessment studies [12,21–23], conservation biology [12,24], or the authentication of sea food [25,26]. As consequence, many recently published species descriptions and taxonomic studies included barcode sequence data [27–31].

Within the Arthropoda, most DNA barcoding publications focus on insects [32–38], whereas the number of comprehensive studies analyzing the utility of DNA barcodes for the discrimination of crustacean species is still limited [39–43]. Nevertheless, crustaceans represent one of the most ecologically and economically important invertebrate groups [44]. Currently, more than 67,000 extant species have been described so far [45], and probably five or ten times of that number are waiting to be discovered in the marine realm [46]. Crustaceans successfully colonized every marine, brackish, and freshwater environment on Earth, and exhibit an astonishing diversity of form, habit, and size. No other group of plants or animals shows a morphological diversity as seen among the extant Crustacea [47], ranging from the tiny tantulocarid species *Stygotantulus stocki* Boxshall & Huys, 1989 with a body length less than 0.1 mm up to the giant spider crab *Macrocheira kaempferi* Temminck, 1836 with a documented leg span of up to 3.7 m [48]. In the case of parasitic crustaceans, many species can only be identified as crustaceans by reference to their larval stages, for example species of the rhizocephalan genus *Sacculina* Thompson, 1836 (Thecostraca) or various copepod species (e.g. the genus *Lernaeenicus* Le Sueur, 1824).

In this study we present a comprehensive DNA barcode library of various crustacean taxa found in the North Sea, one of the most extensively studied ecosystems of the world. The North Sea is characterized by a high amount of anthropogenic pressure such as intensive fishing and ship traffic as well as offshore installations. Environmental parameters (e.g. depth, sediment characteristics, temperature and salinity) of this semi-enclosed shelf sea follow a distinct pattern: high seasonal fluctuations can be observed in southern areas, less fluctuations are found in the northern regions [49,50]. This heterogeneity is also displayed in macrobenthic community structures, with a lower number of species in the shallow southern parts (i.e. the German Bight) and more species in the central and northern North Sea [51–54]. Furthermore, species with a typical Mediterranean-Lusitanian distribution are also known to occur in parts of the North Sea where oceanic influences prevail [55].

Our new barcode library includes a broad coverage of crustacean species of different taxa inhabiting the North Sea, ranging from large king crabs (e.g. *Lithodes maja* (Linnaeus, 1758)) to minute species that are elements of the benthic meiofauna (e.g. *Asellopsis intermedia* (Scott T., 1895)) as well as highly modified parasites of crustaceans (e.g. *Peltogaster paguri* Rathke, 1842) or fish (e.g. *Chondracanthus merluccii* (Holten, 1802)).

## Material and Methods

### Sampling of specimens

All analyzed crustaceans were collected between 2003 and 2014 using various sampling methods (i.e. hand collecting, Van Veen grab sampler, various dredges, bottom trawls). The Nationalparkverwaltung Niedersächsisches Wattenmeer (Wilhelmshaven) and the Johann Heinrich von Thünen-Institut, Abteilung Seefischerei (Hamburg), issued the permission to conduct this study. Our field studies have not involved endangered or protected species. All crustaceans were morphologically identified to species level by eight of the authors (JB, TCK, SL, IM, HN, KP, AS-V, MR) or by other taxonomic experts and matched with the online database World Register of Marine Species [56]. The applied taxonomic classification is based on the most recent system [45] (Table 1).

**Table 1:** Number of barcoded species of different crustacean orders from the North Sea. Note that 136 DNA barcodes of 13 species of the Calanoida (Maxillopoda: Copepoda: Gymnoplea) were already published as part of a previous study [3].

Class	Subclass	Superorder	Order	Number of analyzed species	Number of analyzed specimens
Branchiopoda	Phyllopoda		Diplostraca	5	24
Maxillopoda	Thecostraca	Rhizocephala	Aktentrogonida	1	7
			Kentrogonida	2	8
		Thoracica	Lepadiformes	1	4
			Scalpelliformes	1	6
			Sessilia	7	67
	Copepoda	Gymnoplea	Calanoida	14	157
		Podoplea	Cyclopoida	4	22
			Harpacticoida	10	31
			Siphonostomatoida	5	26
			Monstrilloida	1	8
Malacostraca	Hoplocarida		Stomatopoda	1	1
	Eumalacostraca	Peracarida	Mysida	7	35
			Amphipoda	59	305
			Isopoda	16	84
			Cumacea	4	14
		Eucarida	Euphausiacea	1	4
			Decapoda	66	529
**Total**				**205**	**1332**

For our analysis we also included 136 DNA barcodes of 13 calanoid copepod species of a previous study [3]. Most specimens were collected in the North Sea (*n* = 1,285, 96.5%), but for comparison some specimens from the English Channel (30, 2.3%), the Baltic Sea (6, 0.4%), and some other locations of Germany (11, 0.8%) were also included. All specimens were stored in ethanol (96%). The number of analyzed specimens per species ranged from one individual (26 species, 12.7%) to a maximum of 32 for the long-clawed porcelain crab *Pisidia longicornis* (Linnaeus, 1767) (Malacostraca: Decapoda).

### DNA sequencing and data depository

Laboratory operations were carried out either at the Canadian Center for DNA Barcoding (CCDB), University of Guelph, following standardized high-throughput protocols for DNA barcode amplification and sequencing [57,58], or at the molecular lab of the German Center of Marine Biodiversity Research, Senckenberg am Meer, in Wilhelmshaven, Germany. For small specimens with a body length <3 mm, complete specimens were used for DNA extraction, whereas tissue samples (e.g. legs or pleon muscles) were used for individuals >3 mm. In Wilhelmshaven, DNA was extracted using the QIAmp Tissue Kit (Qiagen GmbH, Hilden, Germany) or NucleoSpin Tissue Kit (Macherey-Nagel, Düren, Germany), following the manufactures protocol. Polymerase chain reaction (PCR) was performed for amplifying the CO1 barcode fragment using two primer pairs (LCO1480/HCO2198 [59]; or jgLCO1490/ jgHCO2198 [60]). For the primer pair jgLCO1490/jgHCO2198 we added M13 forward and reverse tails to provide defined nucleotide sequences for sequencing [61]. All PCR products were amplified using illustra puReTaq Ready-To-Go PCR Beads (GE Healthcare, Buckinghamshire, UK) in a total volume of 20 μl, containing 17.5 μl sterile molecular grade H_2_O, 2 μl DNA template with an DNA amount between 2 to 150 ng/μl, and 0.25 μl of each primer (20 pmol/μl). The PCR thermal conditions included an initial denaturation at 94°C (5 min), followed by 38 cycles at 94°C (denaturation, 45 s), 48°C (annealing, 45 s), 72°C (extension, 80 s), and a final extension step at 72°C (7 min). All PCR reactions were conducted using an Eppendorf Mastercycler Pro system (Eppendorf, Hamburg, Germany). Negative and positive controls were included with each round of reactions. Two μl of the amplified products were verified for size conformity by electrophoresis in a 1% agarose gel with GelRed using commercial DNA size standards, whereas the remaining PCR product was purified with the QIAquick PCR Purification Kit (Qiagen GmbH, Hilden, Germany). Purified amplicons were cycle sequenced and sequenced in both directions at a contract sequencing facility (GATC, Konstanz, Germany) using LCO1480 and HCO2198 as sequencing primers or the M13 sequence tails for jgLCO1490/jgHCO2198 as matrix (see above). Double stranded sequences were assembled and checked for the presence of mitochondrial pseudogenes (numts) with the Geneious version 7.0.4 program package [62] by translating all nucleotide sequences in amino acid sequences. BLAST searches were performed to confirm the identity of all new sequences [63,64].

All analyzed barcodes had a length of at least 500 base pairs (bp). Relevant voucher information, taxonomic classifications, photos, DNA barcodes, used primer pairs and trace files are publicly accessible through the public data set “Crustacea of the North Sea” (Dataset ID: DS-CRNS; dx.doi.org/10.5883/DS-CRNS) on the Barcode of Life Data Systems (BOLD; www.boldsystems.org) [65]. In addition, all barcode sequences were deposited on GenBank (accession numbers KT208391 to KT209586; Bankit: 1835202).

### DNA barcode analysis

Intra- and interspecific nucleotide variability of the analyzed crustaceans was based on the Kimura 2-parameter model (K2P; [66]), using the analytical tools on BOLD (align sequences: BOLD aligner; ambiguous base/gap handling: pairwise deletion). The BOLD workbench was also used to calculate base frequencies. In addition, all barcodes were subject to the Barcode Index Number (BIN) system implemented in BOLD [67]. This approach clusters DNA barcodes to calculate operational taxonomic units (OTUs) that closely correspond to species. BIN clusters are indexed in a regimented way which means that genetically identical taxa of different studies reside under a shared identifier [67]. However, BINs are not necessarily stable over time and may change, for example as a consequence of the addition of new barcode sequences to BOLD. A recommended threshold of 2.2%, as it has been demonstrated in eight test datasets, was used for a rough differentiation of low and high intraspecific as well as interspecific K2P distances [67].

We performed neighbor joining cluster analyses (NJ; [68]) to construct a graphical representation of patterns of nucleotide divergences based on K2P distances using MEGA6.4 [69] for all Copepoda, Amphipoda, Decapoda, Thecostraca, Isopoda, and all other taxa. Non-parametric bootstrap support values were obtained by resampling and analyzing 1,000 replicates [70]. Another NJ analysis (K2P distances) was performed for all analyzed specimen with non-parametric bootstrap replicates (*n* = 1,000). For all analyses, barcodes were aligned using MUSCLE [71], implemented in MEGA6.4. The sequence alignment for all taxa and the MEGA-generated K2P NJ tree file in text format were uploaded to TreeParser [72], producing an output FASTA file that followed the order of terminals in the tree. A Klee diagram was generated by indicator vector analysis [73] with parameters *n* = 1 sequence/vector and bp window size = 10–600.

For species that showed identical haplotypes, statistical maximum parsimony networks were constructed with TCS 1.21 using default settings [74]. Such networks allow the identification of haplotype sharing between species as a consequence of recent speciation or on-going hybridization.

Finally we performed a simulation of sequence-based identification of specimens using the R library SPIDER [75]. Each sequence was used as a query against the entire dataset. Identification was provided following three different criteria: Best Match (BM); Best Close Match (BCM); and All Species Barcode (ASB). The BM criterion assigns identifications to the closest match regardless of the distance. The BCM criterion [76] is similar to BM, but the query is identified by the closest match with a distance below a defined threshold. Finally, the ASB criterion simulates the BOLD ID engine by applying a threshold and querying at all the sequences within it. A query is identified when all the matching sequences below the threshold are conspecific. Results are reported as correct when corresponding to prior morphological identifications, otherwise a result counts as incorrect. For BCM and ASB, a query may provide ambiguous results if sequences divergences of different species are below the threshold (ASB) or sequences from different species are the closest match below threshold (BCM). A query resulting in “no ID” has no match below the defined threshold. For BCM and ASB we used three different thresholds: the value of 1% (K2P), which is the standard used by the BOLD ID engine [65]. As second threshold we used the value that minimizes the cumulative identification errors (function ‘threshVal’ in SPIDER), i.e. the sum of false positive (no conspecific matches within threshold of query) and false negative (sequences from multiple species within threshold). Finally, we used a density plot of genetic distances and evaluated where a minimum in the density corresponds to the transition between intra- and interspecific distances (function ‘localMinima’ in SPIDER) as third threshold. All simulations were run twice, with and without singletons (species represented by a single sequence).

## Results

In total, 1,332 DNA barcodes of 205 species were analyzed. No numts were found. A full list of the analyzed species can be found in the supporting information (S1 Table). Fragment lengths of the analyzed DNA barcodes ranged from 514 to 667 bp. For 129 species (61.4%), five or more DNA barcodes have been generated (S1 Fig). Similar to other arthropod studies, our data indicated a high AT-content for this mitochondrial gene fragment: the mean sequence compositions were A = 26%, C = 19%, G = 19% and T = 36%. Intraspecific K2P divergences ranged from zero to 14.87% whereas interspecific distances were between 0% and 44.38% (S1 Table). The lowest intraspecific distances of clearly distinct barcode clusters were revealed for the closely related spider crab species pair *Hyas araneus* (Linnaeus, 1758) and *Hyas coarctatus* Leach, 1816 with a value of 2.36%. Maximum intraspecific pairwise distances >2.2% were found for 13 species, including one thecostrac, one isopod, three amphipod, three decapod and five copepod species (Table 2). In contrast to this we found low pairwise distances with values <2.2% only for one decapod species pair: *Macropodia parva* Van Noort & Adema, 1985 and *Macropodia rostrata* (Linnaeus, 1761). Unique BINs were revealed for 198 species (96.7%), two BINs for six species (2.9%), and three BINs for the amphipod species *Gammarus salinus* Spooner, 1947 (0.4%).

**Table 2:** Table of 13 species of the Crustacea with a maximum intraspecific distance (K2P) of >2.2%. At least two specimens of the listed species showed a distance value higher than the threshold as part of a pairwise comparison.

Order	Species	Number of analyzed specimens (*n*)	Mean pairwise K2P distance (%)	Maximum pairwise K2P distance (%)	BINs
**Amphipoda**	*Photis longicaudata*	6	0.98	2.36	ACG9506
**Calanoida**	*Temora longicornis*	19	0.48	2.66	AAO2762
**Decapoda**	*Pagurus pubenscens*	12	1.6	2.89	AAB6221
**Calanoida**	*Calanus helgolandicus*	14	0.64	2.99	AAB3934
**Amphipoda**	*Monocorophium insidiosum*	9	1.24	3.41	AAE9749, AAE1628
**Sessilia**	*Austrominius modestus*	11	1.39	3.81	ABX4245, ACR4768
**Decapoda**	*Pandalus montagui*	21	1.49	4	AAB2199
**Amphipoda**	*Gammarus salinus*	6	2.01	4.14	AAB7068, ACG9079, ACG8870
**Calanoida**	*Pseudocalanus elongatus*	9	1.93	4.43	AAF6145
**Decapoda**	*Eriocheir sinensis*	6	2.06	4.78	ABB0750, AAA8754
**Isopoda**	*Astacilla intermedia*	3	3.19	4.79	ACP7495, ACP7496
**Calanoida**	*Anomalocera patersoni*	22	1.87	6.11	ACM8185, ACM8186
**Siphonostomatoida**	*Caligus elongatus*	10	6.9	14.87	AAE8403, AAE8404

**Fig 1:**
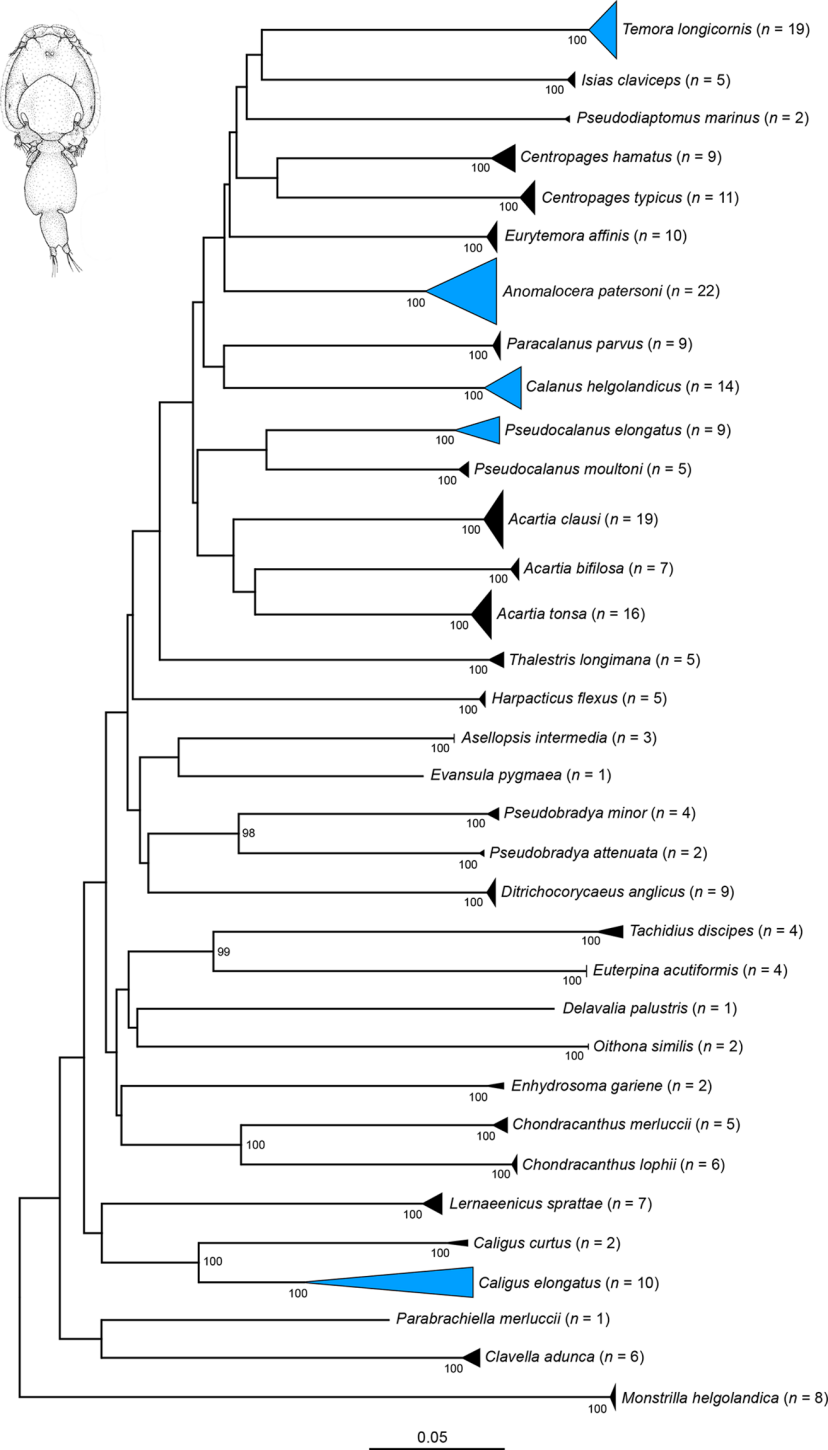
Neighbor joining topology of the analyzed Copepoda based on Kimura 2-parameter distances. The number of analyzed specimens collapsed into a single node is provided following the species name. Triangles indicate the relative number of individual’s sampled (height) and sequence divergence (width). Blue triangles indicate species with intraspecific maximum pairwise distances >2.2%. Numbers next to nodes represent non-parametric bootstrap values >90% (1,000 replicates). Drawing of *Caligus curtus* O.F. Müller, 1789 is taken and modified from a previous publication [77].

**Fig 2:**
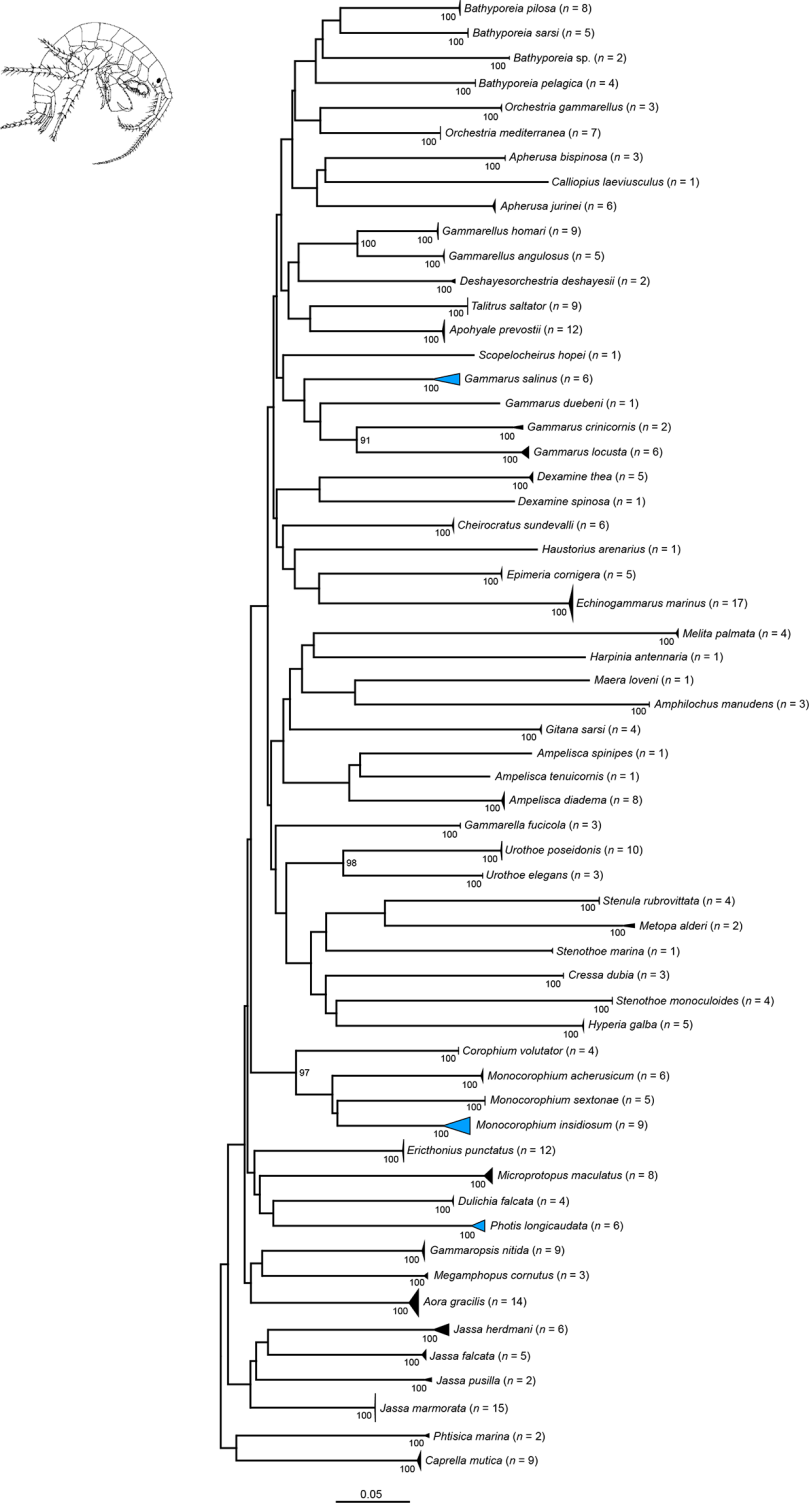
Neighbor joining topology of the analyzed Amphipoda based on Kimura 2-parameter distances. The number of analyzed specimens collapsed into a single node is provided following the species name. Triangles indicate the relative number of individual’s sampled (height) and sequence divergence (width). Blue triangles indicate species with intraspecific maximum pairwise distances >2.2%. Numbers next to nodes represent non-parametric bootstrap values >90% (1,000 replicates). Drawing of *Melita palmata* Montagu, 1804 is taken and modified from a previous publication [78].

**Fig 3:**
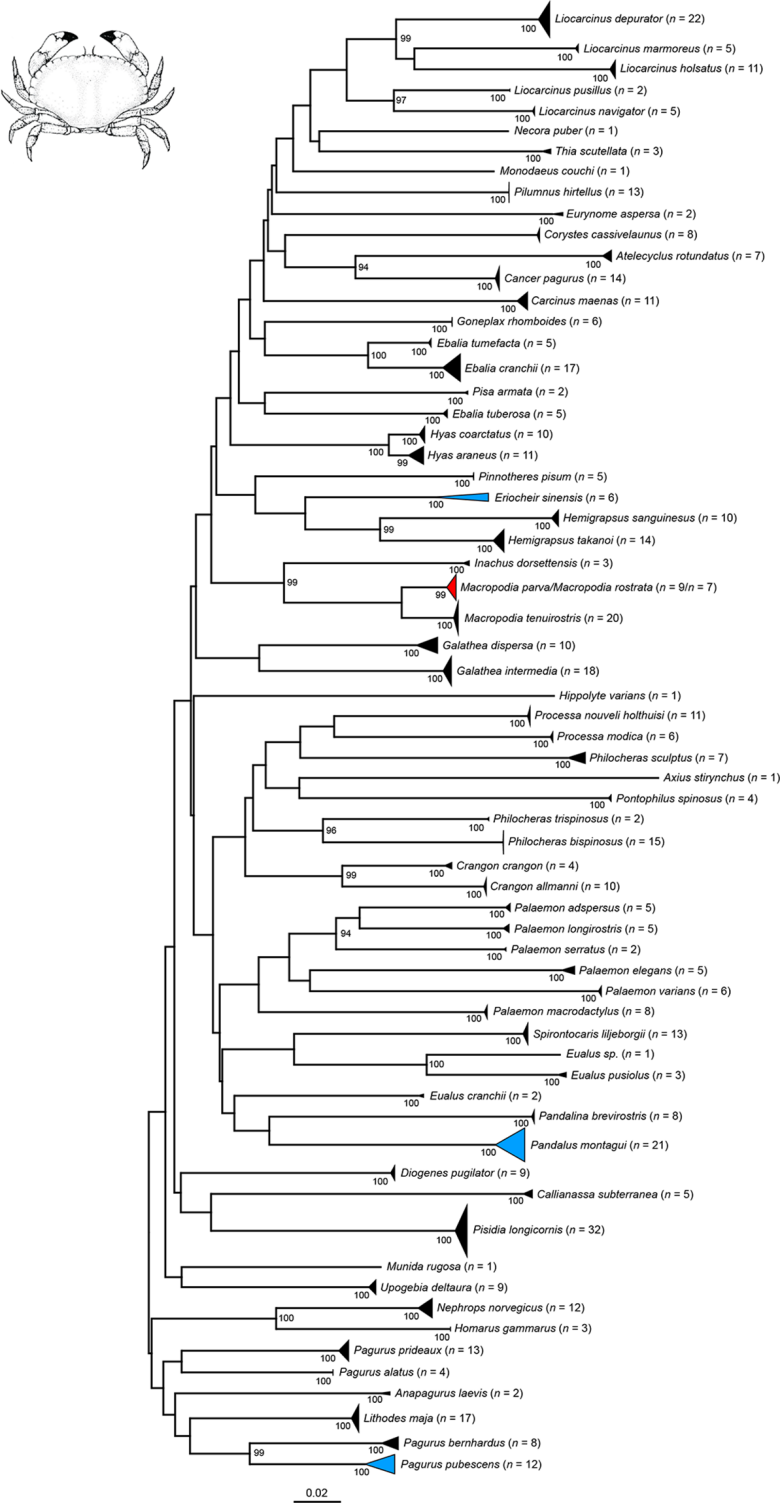
Neighbor joining topology of the analyzed Decapoda based on Kimura 2-parameter distances. The number of analyzed specimens collapsed into a single node is provided following the species name. Triangles indicate the relative number of individual’s sampled (height) and sequence divergence (width). Blue triangles indicate species with intraspecific maximum pairwise distances >2.2%), the red triangle species with interspecific distance values <2.2%. Numbers next to nodes represent non-parametric bootstrap values >90% (1,000 replicates). Drawing of *Cancer pagurus* Linnaeus, 1758 is taken and modified from a previous publication [79].

**Fig 4:**
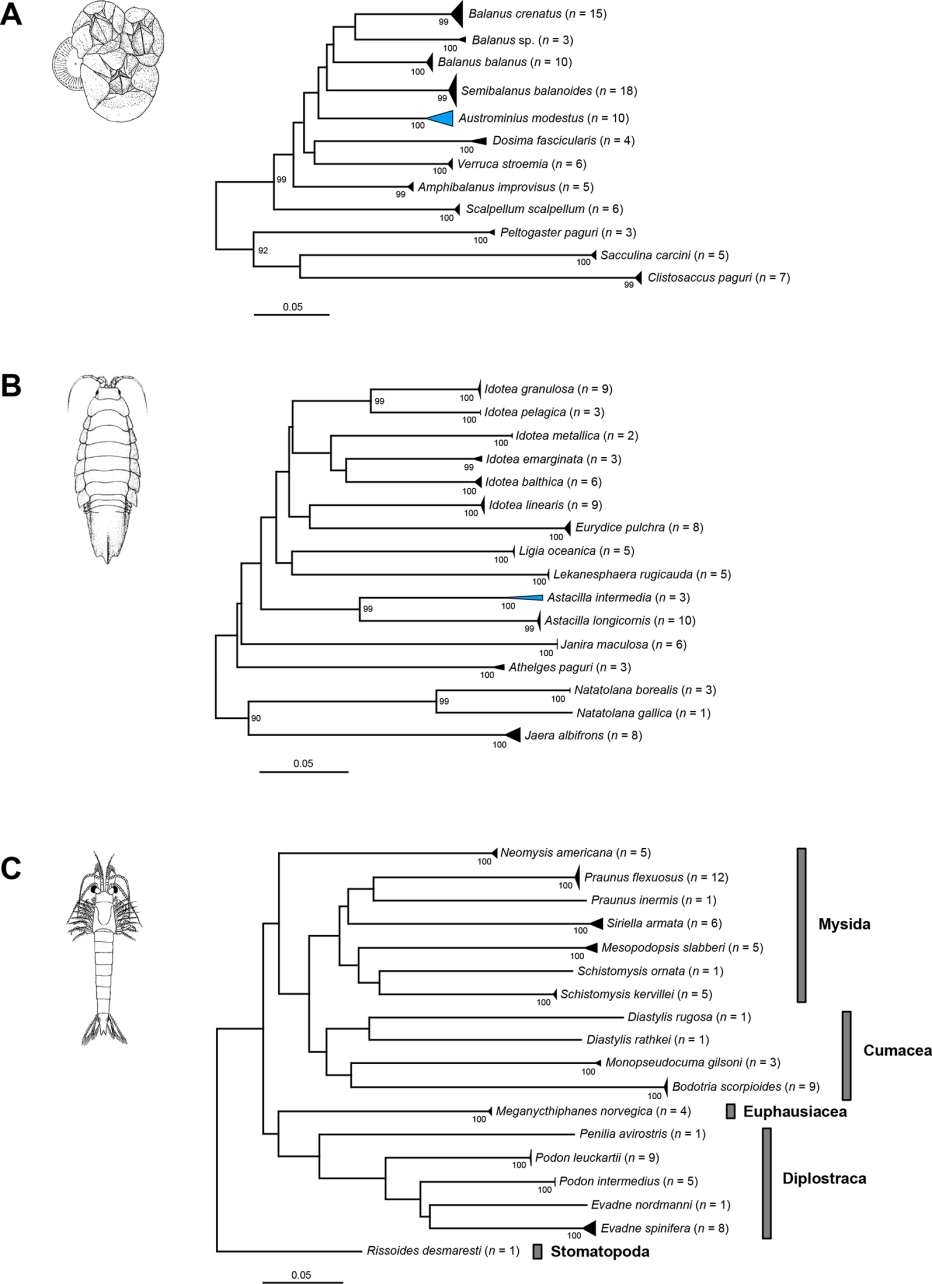
Neighbor joining topologies of various crustacean taxa based on Kimura 2-parameter distances. The number of analyzed specimens collapsed into a single node is provided following the species name. Triangles indicate the relative number of individual’s sampled (height) and sequence divergence (width). Blue triangles indicate species with intraspecific maximum pairwise distances >2.2%. Numbers next to nodes represent non-parametric bootstrap values >90% (1,000 replicates). Drawings of species are taken and modified from previous publications (*Balanus crenatus* Bruguière, 1789 (A) and *Praunus inermis* (Rathke, 1843) (C): [79]; *Idotea balthica* (Pallas, 1772) (B): [80]).

Our NJ analyses based on K2P genetic distances revealed non-overlapping species clusters with bootstrap support values of 99 or 100% for all Copepoda (Fig 1), all Amphipoda (Fig 2), most Decapoda (Fig 3) as well as all Thecostraca, Isopoda, Cumacea, Diplostraca, Euphausiacea, Mysida and Stomatopoda (all in Fig 4). A NJ topology of all analyzed crustacean specimens based on K2P is presented in the supporting information (S2 Fig).

**Fig 5:**
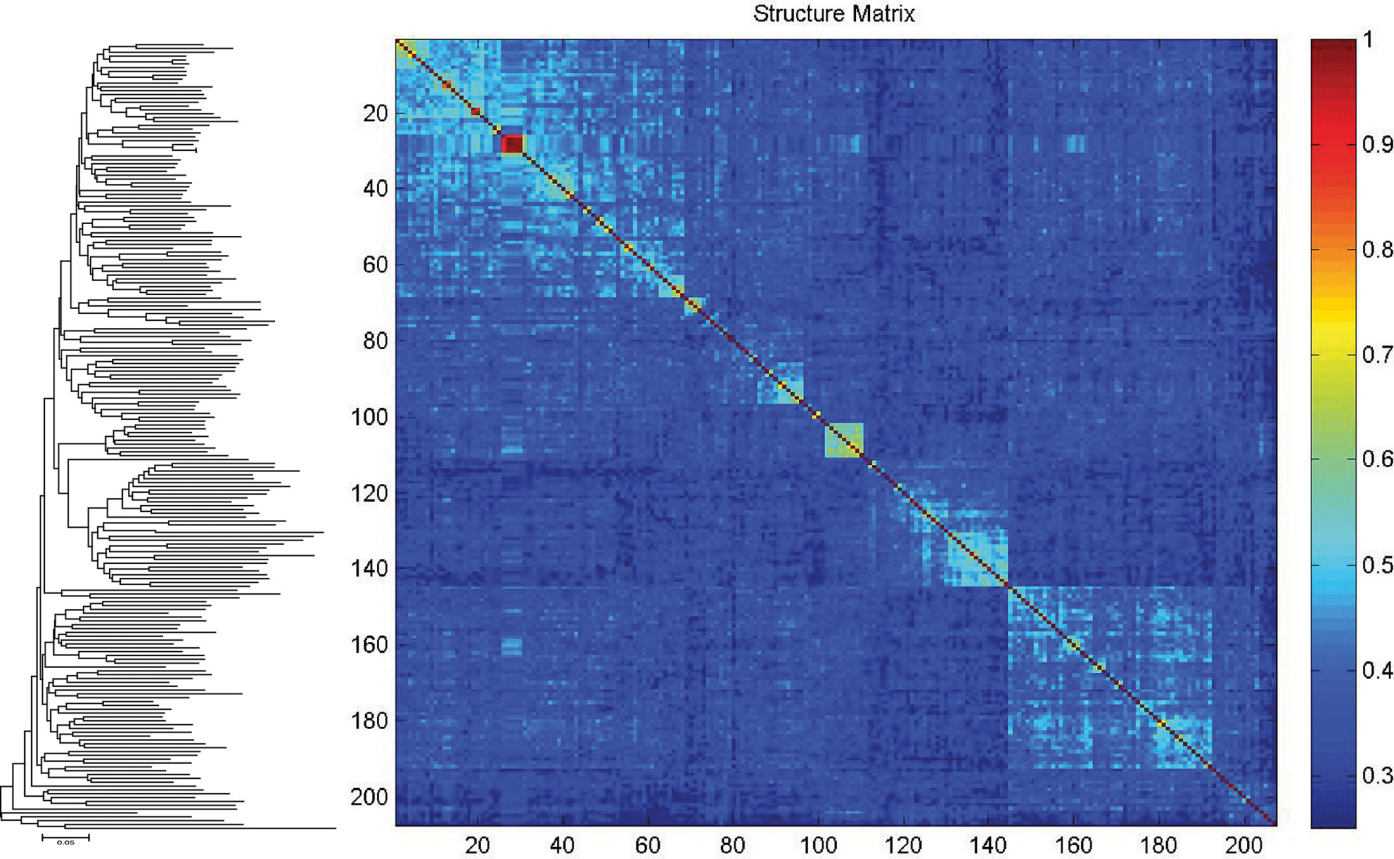
Klee diagram of the analyzed crustacean species. The image was generated from a TreeParser-ordered alignment with a correlation scale presented at the right of the diagram. Sequence clusters appear as blocks of higher correlation along the diagonal, on the left a corresponding NJ topology in identical order (see S3 Fig for details).

A Klee diagram of the TreeParser-ordered alignment showed blocks of high correlation on the diagonal, reflecting the affinity among species (Fig 5). The used NJ topology is presented in the supporting information (S3 Fig). Maximum correlation was observed among neighboring species, and decorrelation among more distant species. Given the broad sampling across all crustaceans the latter occurs much more frequently. Unusual strong correlation was observed among two species (*Macropodia parva* and *Macropodia rostrata*) as a result of haplotype sharing.

**Fig 6:**
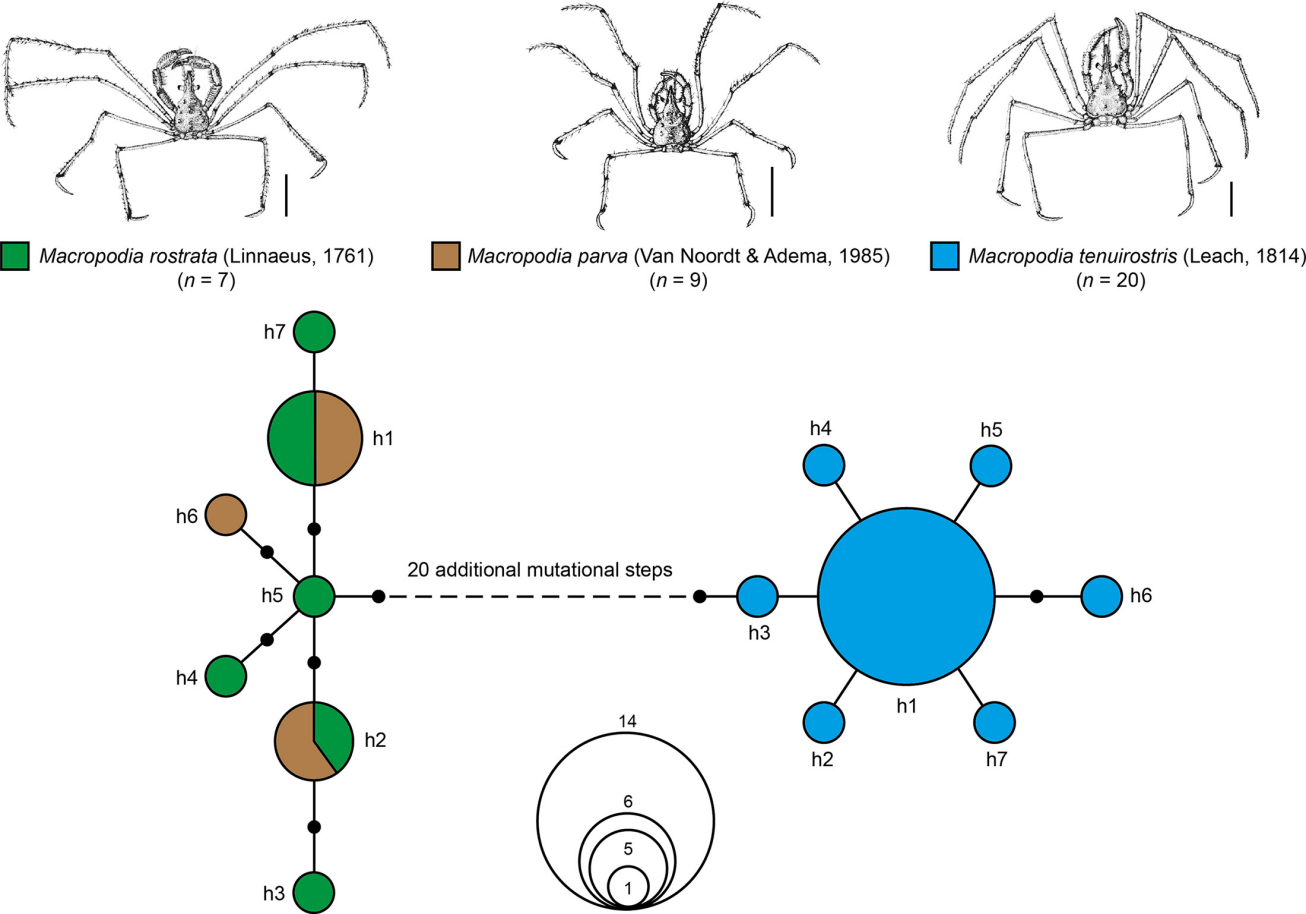
Maximum statistical parsimony network of the three analyzed *Macropodia* species. Settings included a user specified maximum of connection steps at 25 and gaps treated as fifth state. Each line in the network represents a single mutational change; small black dots indicate missing haplotypes. The numbers of analyzed specimens (*n*) are listed, while the diameter of the circles is proportional to the number of haplotypes sampled (see given Open circles with numbers). Scale bars = 1 cm. Illustrations were taken and modified from a previous publication [81].

The statistical maximum parsimony analysis also revealed multiple sharing of haplotypes for *Macropodia parva* (*n* = 9) and *Macropodia rostrata* (*n* = 7) (Fig 6). In total, seven haplotypes were identified for both species. Two haplotypes were shared by specimens of both species (h1 and h2), whereas the remaining five haplotypes were only scored in one specimen (singletons), with one haplotype found for *Macropodia parva* and four for *Macropodia rostrata*. K2P distances ranged from 0.48 to 1.12%. The nearest neighbor species was *Macropodia tenuirostris* (Leach, 1814) (*n* = 20, 7 haplotypes) with one dominant haplotype h1 (*n* = 14), separated by more than 20 additional mutational steps from the *Macropodia parva*/*rostrata* cluster. The minimum distance value between the *Macropodia parva*/*rostrata* cluster and *Macropodia tenuirostris* was 4.32%.

Using SPIDER, we obtained 1,292 correct and 40 incorrect identifications for the BM approach (Table 3). In total, 26 identifications were associated to singletons without conspecific sequences to match. After removing these singletons, incorrect identifications were reduced to 14 (*Macropodia parva* and *Macropodia rostrata*). Details of sequence comparisons are available as supporting information (S2 Table). Using a threshold of 1% as applied in the BOLD ID engine, for the BCM and ABB approaches a value of 2.1% was found for the threshold optimization method whereas a value of 3.7% was proposed by the local minima approach, respectively. For the BCM approach, correct identifications ranged from 1,249 (1% threshold) to 1,288 (3.7% threshold). Sequences with no ID ranged from 68 (1% threshold) to 29 (3.7% threshold). Incorrect and ambiguous identifications were one and 14 with all threshold values, respectively. The exclusion of singletons exclusively influenced the identifications without ID, reducing them to 42 (1% threshold), six (2.1% threshold), and three (3.7% threshold). For the ASB approach, correct identifications ranged from 1,248 (1% and 2.1% thresholds) to 1,266 (3.7% threshold), ambiguous identifications ranged from 16 (1% and 2.1% thresholds) to 37 (3.7% threshold), and identification without ID had values from 68 (1% threshold) to 29 (3.7% threshold). After removing the singletons, only the identifications without ID were reduced to 42 (1% threshold), six (2.1% threshold), and three (3.7% threshold).

**Table 3:** Results of the identification simulations using Best Match (BM), Best Close Match (BCM) and All Species Barcode (ASB) criteria based on SPIDER. Correct and incorrect identifications indicate positive and negative outcome for the respective test. An “ambiguous” outcome corresponds to the presence of both correct and incorrect identifications within the threshold (ASB) or more than one equally close match with different identification including the correct one (BCM). A “no ID” outcome corresponds to no matches found within the threshold (both BCM and ASB). Values in brackets represent the results of simulation with the exclusion of singletons.

	BM	BCM 1%	BCM 2.1%	BCM 3.7%	ASB 1%	ASB 2.1%	ASB 3.7%
**Correct**	1292 (1295)	1249 (1249)	1285 (1285)	1288 (1288)	1248 (1248)	1248 (1248)	1266 (1266)
**Incorrect**	40 (14)	1 (1)	1 (1)	1 (1)	- (-)	- (-)	- (-)
**Ambiguous**	-	14 (14)	14 (14)	14 (14)	16 (16)	16 (16)	37 (37)
**No ID**	-	68 (42)	32 (6)	29 (3)	68 (42)	32 (6)	29 (3)

## Discussion

Our sequence library represents an important step towards the application of DNA barcodes for the identification of crustacean taxa in the North Sea. For 86 taxa (42%), our sequence data represent the first published DNA barcodes. In total, unique BINs were found for 198 (96.6%) species, indicating a high coverage of unique BINs and analyzed species. Shared haplotypes were only found for two decapod species, whereas high intraspecific distances were only documented in one copepod species. This high efficiency of specimen identification was corroborated by our simulations using SPIDER, which were based on three criteria each with a different level of tolerance. The BM approach provided the highest number of correct identifications, i.e. the largest congruence between sequence-based identifications and prior taxonomic assignments based on morphology. However, this approach has an intrinsic bias, for it only considers the closest match regardless of distance. In fact, even singletons received a taxonomic assignment, and the amount of discordance with prior morphological identification was the highest (Table 3). Therefore, this method cannot be recommended as long as the reference database is not complete, as it is currently the case for most marine invertebrates. By using a distance threshold (BCM and ASB) it was possible to highlight cases of low interspecific distances (“ambiguous” entries in Table 3) and to exclude matches with high distances (“no ID” entries in Table 3). Of all used approaches, the ASB is the strictest one, providing no identification if query sequence matches were found below the proposed threshold. This criterion highlights specimens requiring further investigation, either for potential cases of cryptic diversity or misidentifications. We like to point out that the approaches used in our simulation are not to be confused with “species delimitation” [82]. Certainly, some concepts applied here overlap with those used for species delimitation, but our simulations exclusively tested the performance of a molecular dataset for identifying represented species. We believe that for species delimitation, and thus discovery of putative cryptic species, a different sampling design should be applied covering specimens from various locations of the entire known distribution of a species.

Whereas the species numbers of many terrestrial taxa in Europe are well known, e.g. the diurnal butterflies of Europe [83], ground beetles of Germany [84], or grasshoppers of East Austria [85], no comprehensive and reliable information about the total number of crustaceans or at least specific groups of the North Sea are available. For many terrestrial taxa, e.g. birds, reptiles and various insects, a large number of active amateurs continuously help updating distribution maps and check lists. Unfortunately, this is not the case for marine crustaceans of the North Sea, and until now only very rough estimates exist for many taxa. Most available lists rely on taxon-specific publications [86,87] or national red lists that focus on specific areas of the North Sea [88]. As consequence we are unable to provide valid estimates of species coverage for the crustacean taxa analyzed in our study. In the following we will discuss our results of the analyzed species of the Thecostraca, Copepoda, Decapoda, Amphipoda and Isopoda in detail.

### Maxillopoda: Thecostraca

The Thecostraca are a large assemblage of diverse crustaceans in which parasitism and the adaption to unusual habitats resulted in a wide range of unusual morphologies and life styles [89]. Within the Thecostraca, barnacles are the best known species that typically can be found in the rocky intertidal with high abundance and which pose severe problems as biofouling organisms that settle and accumulate on wetted submerged, man-made surfaces [90]. As a consequence of the growing global cargo ship traffic [91,92], numerous species have become known as invasive species, e.g. *Austrominius modestus* (Darwin, 1854) which invaded the North Sea in the 1940s from Australia and/or New Zealand [93,94]. While nine barnacle species are recorded for the German sectors of the North and Baltic Sea [95], the total number of thecostracan species inhabiting the North Sea is unknown. In our study we generated 92 barcodes of 12 species, including one currently unknown barnacle species (*Balanus* sp.) and three parasitic taxa (*Clistosaccus paguri* Lilljeborg, 1861, *Peltogaster paguri* Rathke, 1842, and *Sacculina carcini* Thompson, 1836). All analyzed species form distinct sequence clusters, supported by high bootstrap values (Fig 4A). With one exception (*Austrominius modestus*), all analyzed species possess unique BINs. For this taxon we found a somewhat higher genetic variability among the five studied specimens (maximum pairwise K2P distance: 3.81%) as well as two BINs (Table 2). However, the analyzed specimens form a monophyletic lineage with high bootstrap support (100%). Additional specimens from different localities would be useful to analyze these results more in detail. Nevertheless, our results clearly show that the application of DNA barcoding is highly successful for the identification of thecostracan specimens of the North Sea.

### Maxillopoda: Copepoda

With more than 15,000 described species to date [45], copepods are the dominant component of the holozooplankton, both numerically and in terms of biomass [96], and represent an essential element of aquatic food chains [97,98]. The number of calanoid copepods of the North Sea ranges between 15 and 25 species [99], and about 50 parasitic species of fish are known [87,100]. In contrast to this, the total number of harpacticoid copepods is still unclear. A synoptic meiobenthic survey of 171 stations in the North Sea, ranging from the Straits of Dover in the South to the 100 m isobath in the North, revealed 278 copepod species, with more than 40% of them being new to science [86]. Ten coastal harpacticoid copepod species were included in our study, representing a very first step to analyze this taxonomically difficult but vast group of tiny crustaceans using DNA barcodes. Similar to all other analyzed copepod species the harpacticoids grouped unambiguously with high bootstrap support (Fig 1). This efficacy of DNA barcoding for the identification of copepod species has been already demonstrated in various studies for other regions [40,43,101]. Nevertheless, we found five species of copepods with maximum pairwise distances >2.2%, including four species of the Calanoida (*Temora longicornis* (Müller O.F., 1785) with 2.76%, *Calanus helgolandicus* (Claus, 1863) with 2.99%, *Pseudocalanus elongatus* (Boeck, 1865) with 4.43%, and *Anomalocera patersoni* Templeton, 1837 with 6.11%) and one species of the Siphonostomatoida (*Caligus elongatus* Nordmann, 1832 with 14.87%) (Table 2). We found no distinct lineages within the four analyzed calanoid species, but our data revealed two distinct monophyletic clusters within the parasitic copepod *Caligus elongatus* with distances ranging from 6.9 to 14.87% (Table 2). These results are concordant with previous studies which also found two different genotypes within this sea louse species [102–104]. As part of these studies, the molecular analyses two mitochondrial genes (16S rDNA, CO1) and a number of selected morphological characters gave evidence of the presence of two sibling species [104]. Unfortunately, a final taxonomic revision of this abundant taxon is still missing.

### Malacostraca: Peracarida: Amphipoda

Aside from the Isopoda and Tanaidacea, the ecologically diverse order of the Amphipoda is one of the most species-rich groups within the Peracarida with more than 170 extant families and approx. 10,000 described species so far [45]. For the German sectors of the North and Baltic Sea, 186 amphipod species are recorded [88]. As part of our study we analyzed 305 specimens representing 59 species. All species formed monophyletic clusters with high bootstrap support (Fig 2). Nevertheless, maximum pairwise distances with values higher than 2.2% were revealed for three species: *Photis longicaudata* (Bate & Westwood, 1862) with 2.36% and one BIN, *Monocorophium insidiosum* (Crawford, 1937) with 3.41% and two BINs, and *Gammarus salinus* Spooner, 1947 with 4.14% and three BINs (Table 2). Due to the fact that the taxonomic status of all three species is uncontested, we assume that the observed variability may result from phylogeographic processes that have also been documented for other amphipod species inhabiting the North and Baltic Sea [105–108].

### Malacostraca: Peracarida: Isopoda

The Isopoda comprise about 10,500 described marine, freshwater and terrestrial species [45]. About 28 species are documented for the German sectors of the North and Baltic Sea [88]. Similar to almost all other analyzed crustacean taxa our barcode analysis revealed coherent monophyletic clusters with high bootstrap support for all analyzed 16 species (Fig 4B). We found maximum pairwise distances higher than 2.2% only within one species, namely *Astacilla intermedia* (Goodsir, 1841) with 4.79% and two BINs (Table 2). For this species, two of the three analyzed female specimens showed identical barcodes sequences. Given the fact that all three specimens are morphologically highly similar and valid identifications are solely based on male characteristics, we are currently unable to ascertain if the observed genetic distances simply represent a high level of intraspecific variation or reflect cryptic diversity. To answer this question, more specimens need to be collected and analyzed, using both morphological characters and nuclear sequence data [30,109].

### Malacostraca: Eucarida: Decapoda

With approx. 15,000 described species [45], the Decapoda represent one of the best-known taxa of the Crustacea. Decapod crustaceans are familiar to most people and represent a dominant group of benthic invertebrates of the continental shelf and slope, including many species of economic importance [42]. Thus, it is no surprise that many barcoding studies in the past focused on decapods [29,31,39–41,110,111]. Despite of their economic and ecological importance, the total number of decapod species that inhabit the North Sea is still unclear. For the German sectors of the North and Baltic Sea, 76 species of decapod crustaceans are documented [88]. As part of this study, 529 specimens of 66 decapod species were analyzed. With one exception (see below), all analyzed species can be characterized as monophyletic clusters with high bootstrap support (99–100%) (Fig 3). Within the analyzed taxa, three species showed intraspecific maximum pairwise distances greater than 2.2%: *Pagurus pubescens* Krøyer, 1838 (2.89%, one BIN), *Pandalus montagui* Leach, 1814 (4%, one BIN), and *Eriocheir sinensis* Milne Edwards, 1853 (4.78%, two BINs) (Table 2). Whereas the observed molecular variability of *Pagurus pubescens* and *Pandalus montagui* may result from phylogeographic effects, a previous barcoding study already highlighted problems in species identification within the crab genus *Eriocheir* De Haan, 1835 as a consequence of unresolved taxonomy [40]. In our case, the observed high distances for the notorious invasive Chinese mitten crab *Eriocheir sinensis* were caused by one specimen that was sampled downstream of the river Rhine close to Bonn (approx. 350 km from the river mouth). All other four specimens were collected at the coast, showing distance values ranging from zero to 0.5%. Interestingly, all specimens could be identified by morphological characters without any difficulties. It is obvious that a comprehensive taxonomic revision of this important genus using both morphological and molecular data is urgently needed.

Our data also revealed haplotype sharing for *Macropodia parva* van Noort and Adema, 1985 and *Macropodia rostrata* (Linnaeus, 1761) (Fig 6). Morphological differences between both species of the family Inachidae are generally very subtle, plastic and difficult to spot, e.g. the presence and size of spines on the fifth pereiopod, the curvature of the dactylus of the fifth pereiopod and the length of the rostrum [81]. For the analyzed barcode fragment, both species shared identical haplotypes, and there was no evidence for any differentiation between them. Nevertheless, when species pairs have very recent origins or hybridize, the utility of mtDNA sequences for species identification is very limited [112,113]. After the initial “split”, the new sister species will share alleles and mutations especially in slowly evolving genes [31,112,113], and faster evolving nuclear markers as SNPs or RAD tags may be more useful for species delineation [114–117]. Based on our data, however, it is also possible that the species pair represents one species and *Macropodia parva* needs to be synonymized with *Macropodia rostrata*. Additional analyses of morphological as well as molecular data have to be performed to answer this question.

### Other analyzed crustacean taxa

In addition to the already discussed data, various other crustacean taxa were analyzed, including five species of the Diplostraca, seven species of the Mysida, four species of the Cumacea, one species of the Euphausiacea and one species of the Stomatopoda. For all these taxa, the total number of species occurring in the North Sea is unknown. All analyzed species, however, formed cohesive clusters with high support, each correlating with a single BIN (Fig 4C). For all taxa, maximum pairwise distances were lower than 2.2%.

## Conclusions

Our data represent the first important step towards the establishment of a comprehensive DNA barcode library of the Crustacea of the North Sea. Despite the fact that various taxa are still missing (e.g. Tanaidacea or Ostracoda) or are currently underrepresented (e.g. harpacticoid copepods), our results clearly underline the usefulness of DNA barcodes to discriminate the vast majority of the analyzed species. It should be also kept in mind that the benefits of DNA barcoding are not restricted to taxonomic or systematic research only. The rise of modern high-throughput sequencing technologies will change biomonitoring applications and surveys in the coming years significantly [23,118,119]. As consequence, reference datasets such as ours will become essential for the correct identification of specimens sequenced as part of metabarcoding studies. This is especially true for the North Sea, a marine region that has been massively affected by cargo ship traffic, the exploitation of oil and gas resources, the rise of offshore wind parks and in particular extensive long-term fisheries.

## Supporting Information

S1 Fig Frequency histogram of the number of barcodes per species. Twenty six were represented by one barcode (12.7%), whereas 129 species (61.4%) had five or more DNA barcodes.
(TIF)

S2 Fig Neighbor joining topology of all analyzed crustacean specimens based on Kimura 2-parameter distances. Specimens are classified using ID numbers from BOLD and species name. Numbers next to nodes represent non-parametric bootstrap values (1,000 replicates, in %).
(PDF)

S3 Fig Neighbor joining topology of all analyzed crustacean species based on Kimura 2-parameter distances used for the Klee diagram. (PDF)

S1 Table Molecular distances based on the Kimura 2-parameter model of the analyzed specimens of the Crustacea. Divergence values were calculated for all studied sequences, using the Nearest Neighbor Summary implemented in the Barcode Gap Analysis tool provided by the Barcode of Life Data System (BOLD). Align sequencing option: BOLD aligner (amino acid based HMM), ambiguous base/gap handling: pairwise deletion. ISD = intraspecific distance. BINs are based on the barcode analysis from 02-06-2015. AphiaID codes were retrieved from the World Register of Marine Species (WoRMS; www.marinespecies.org) on 02-06-2015. Asterisks indicate new species to BOLD.
(DOCX)

S2 Table Results of the SPIDER simulations of sequence-based specimen identification. Query: Each sequence (excluded singletons) used as a query against the complete crustacean COI library (including singletons). Best Match: Results of query identification using the Best Match criterion. BCM: results of identifications based on Best Close Match criterion and three different thresholds (1%, 2.1% and 3.7%). Results were: "correct" or "incorrect" depending on the comparison with our prior morphological identification; "ambiguous" when the simulations returned more sequences from different species with the same distance from the query and below the given threshold; and "no ID" when no matches were found below the given threshold. ASB: results of identifications based on All Species Barcodes and three different thresholds (1%, 2.1% and 3.7%).
(XLSX)
