# Supplementary figures and images for: The Application of DNA Barcodes for the Identification of Marine Crustaceans from the North Sea and Adjacent Regions
Source: PLoS One. 2015 Sep 29;10(9):e0139421. doi: 10.1371/journal.pone.0139421 (PMC4587929; doi:10.1371/journal.pone.0139421)

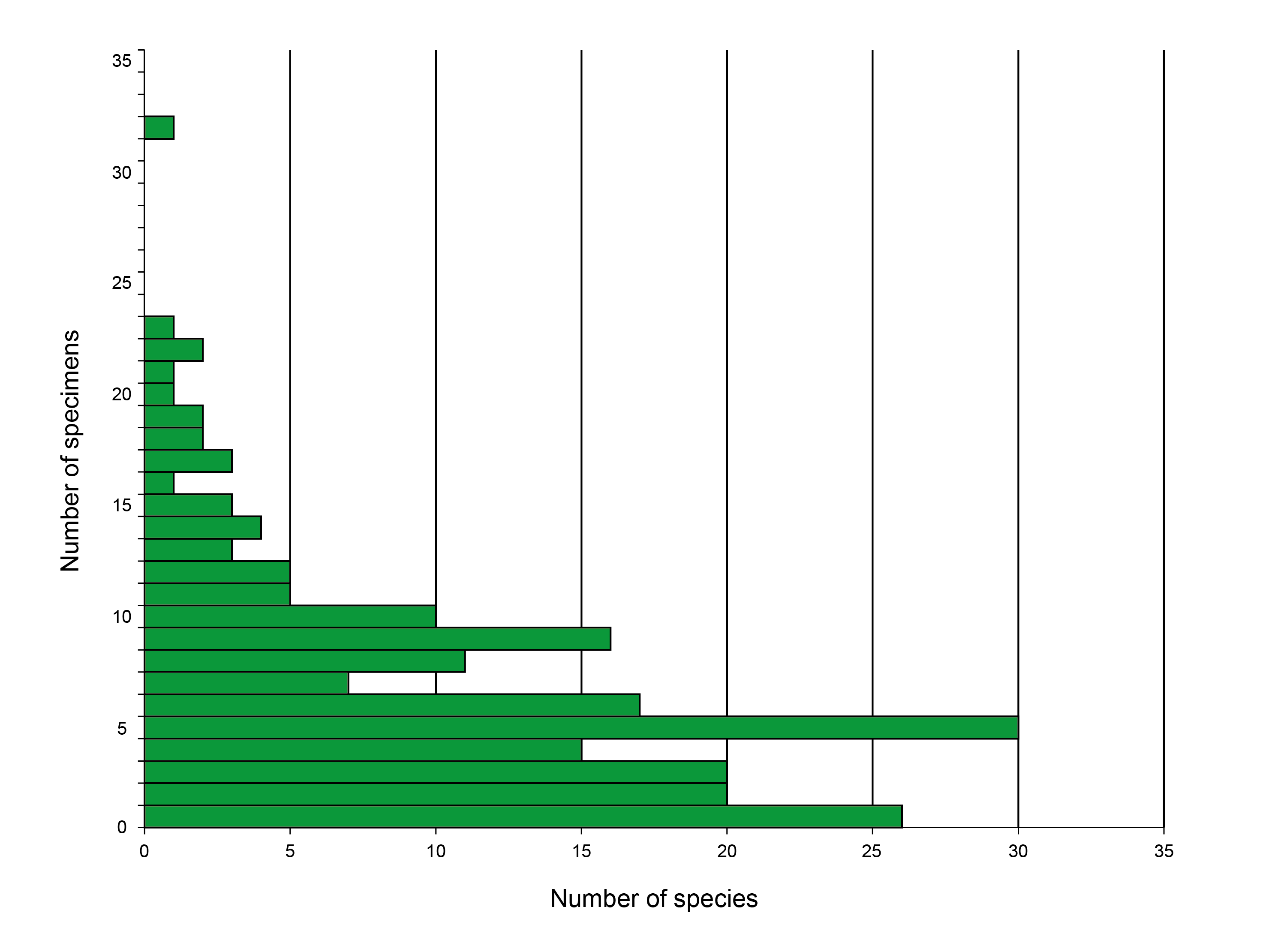

Supplement: S1 Fig — Twenty six were represented by one barcode (12.7%), whereas 129 species (61.4%) had five or more DNA barcodes. (TIF) [file pone.0139421.s001.tif]

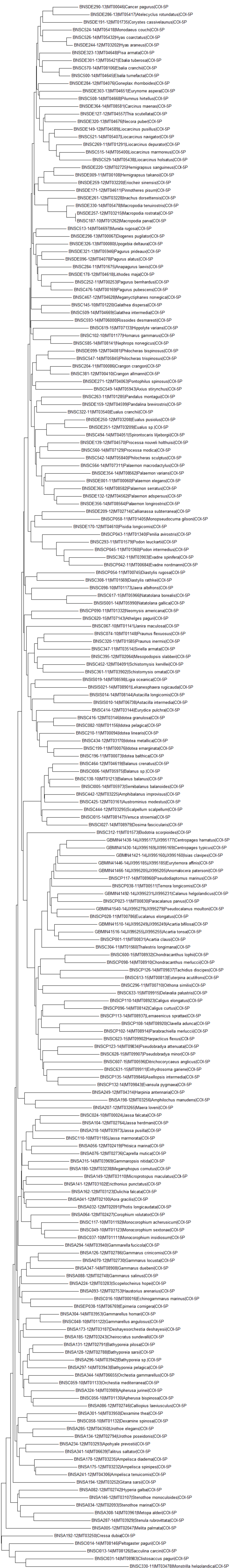

0.05

Supplement: S3 Fig — (PDF) [file pone.0139421.s003.pdf]
