# Supplementary material for: The Application of DNA Barcodes for the Identification of Marine Crustaceans from the North Sea and Adjacent Regions
Source: PLoS One. 2015 Sep 29;10(9):e0139421. doi: 10.1371/journal.pone.0139421 (PMC4587929; doi:10.1371/journal.pone.0139421)
Supplement: S2 Fig — Specimens are classified using ID numbers from BOLD and species name. Numbers next to nodes represent non-parametric bootstrap values (1,000 replicates, in %). (PDF) [file pone.0139421.s002.pdf]

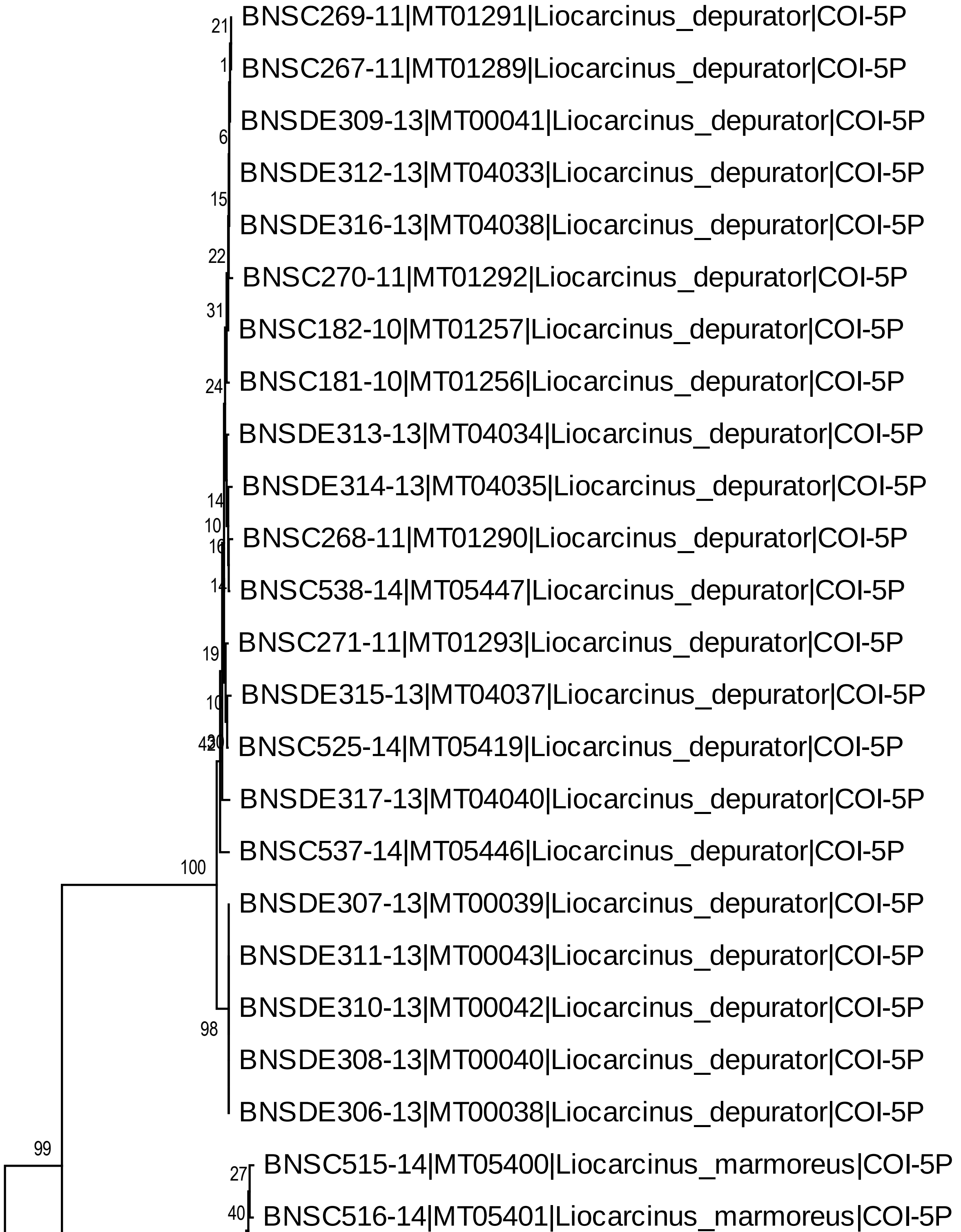
BNSC269-11|MT01291|Liocarcinus_depurator|COI-5P
BNSC267-11|MT01289|Liocarcinus_depurator|COI-5P
BNSDE309-13|MT00041|Liocarcinus_depurator|COI-5P
BNSDE312-13|MT04033|Liocarcinus_depurator|COI-5P
BNSDE316-13|MT04038|Liocarcinus_depurator|COI-5P
BNSC270-11|MT01292|Liocarcinus_depurator|COI-5P
BNSC182-10|MT01257|Liocarcinus_depurator|COI-5P
BNSC181-10|MT01256|Liocarcinus_depurator|COI-5P
BNSDE313-13|MT04034|Liocarcinus_depurator|COI-5P
BNSDE314-13|MT04035|Liocarcinus_depurator|COI-5P
BNSC268-11|MT01290|Liocarcinus_depurator|COI-5P
BNSC538-14|MT05447|Liocarcinus_depurator|COI-5P
BNSC271-11|MT01293|Liocarcinus_depurator|COI-5P
BNSDE315-13|MT04037|Liocarcinus_depurator|COI-5P
BNSC525-14|MT05419|Liocarcinus_depurator|COI-5P
BNSDE317-13|MT04040|Liocarcinus_depurator|COI-5P
BNSC537-14|MT05446|Liocarcinus_depurator|COI-5P
100
BNSDE307-13|MT00039|Liocarcinus_depurator|COI-5P
BNSDE311-13|MT00043|Liocarcinus_depurator|COI-5P
BNSDE310-13|MT00042|Liocarcinus_depurator|COI-5P
98
BNSDE308-13|MT00040|Liocarcinus_depurator|COI-5P
BNSDE306-13|MT00038|Liocarcinus_depurator|COI-5P
99
BNSC515-14|MT05400|Liocarcinus_marmoreus|COI-5P
BNSC516-14|MT05401|Liocarcinus_marmoreus|COI-5P

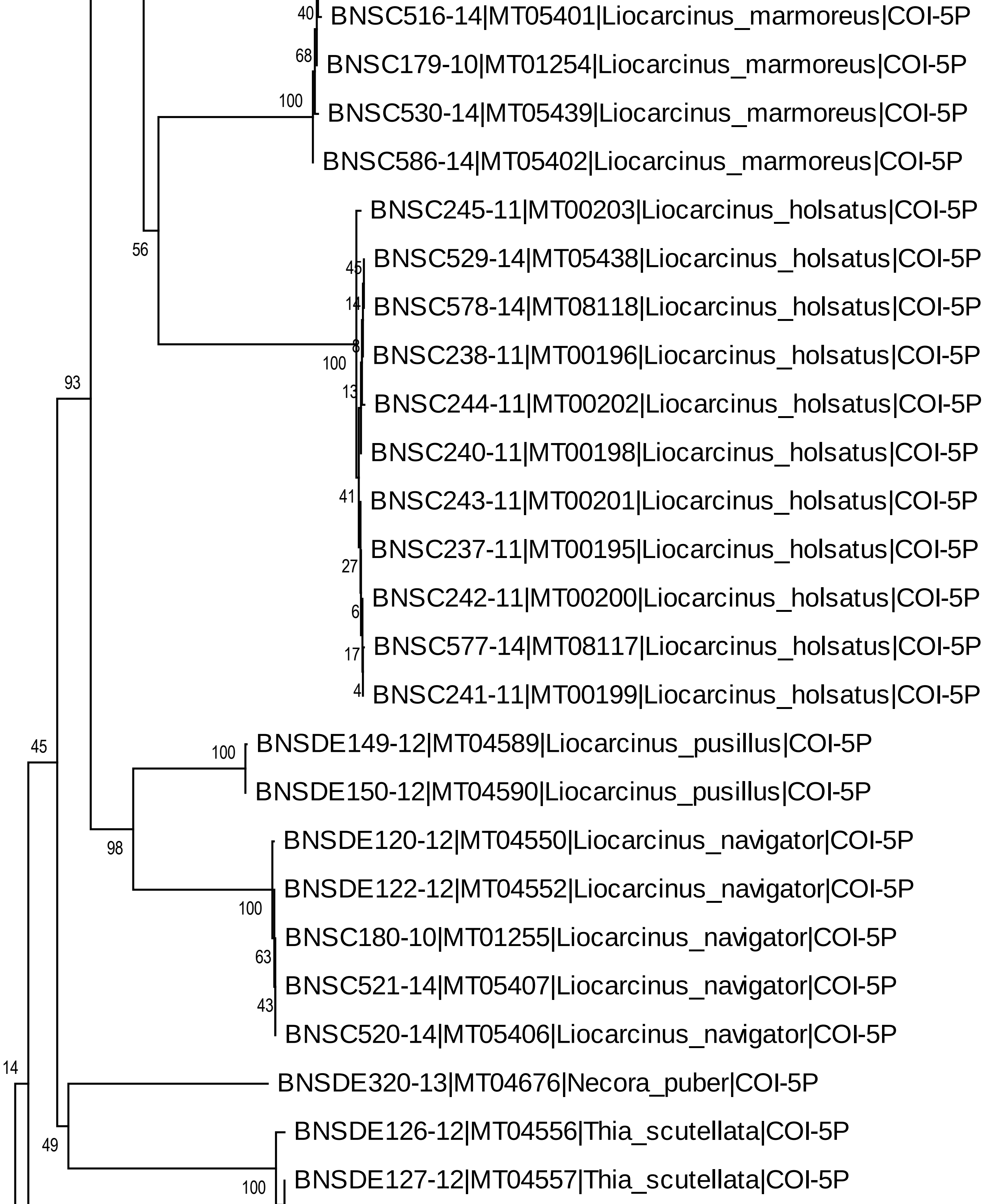

40
BNSC516-14|MT05401|Liocarcinus_marmoreus|COI-5P
68
BNSC179-10|MT01254|Liocarcinus_marmoreus|COI-5P
100
BNSC530-14|MT05439|Liocarcinus_marmoreus|COI-5P
BNSC586-14|MT05402|Liocarcinus_marmoreus|COI-5P
BNSC245-11|MT00203|Liocarcinus_holsatus|COI-5P
56
45
BNSC529-14|MT05438|Liocarcinus_holsatus|COI-5P
14
BNSC578-14|MT08118|Liocarcinus_holsatus|COI-5P
8
BNSC238-11|MT00196|Liocarcinus_holsatus|COI-5P
100
93
13
BNSC244-11|MT00202|Liocarcinus_holsatus|COI-5P
BNSC240-11|MT00198|Liocarcinus_holsatus|COI-5P
41
BNSC243-11|MT00201|Liocarcinus_holsatus|COI-5P
BNSC237-11|MT00195|Liocarcinus_holsatus|COI-5P
27
BNSC242-11|MT00200|Liocarcinus_holsatus|COI-5P
6
17
BNSC577-14|MT08117|Liocarcinus_holsatus|COI-5P
4
BNSC241-11|MT00199|Liocarcinus_holsatus|COI-5P
45
100
BNSDE149-12|MT04589|Liocarcinus_pusillus|COI-5P
BNSDE150-12|MT04590|Liocarcinus_pusillus|COI-5P
BNSDE120-12|MT04550|Liocarcinus_navigator|COI-5P
98
BNSDE122-12|MT04552|Liocarcinus_navigator|COI-5P
100
BNSC180-10|MT01255|Liocarcinus_navigator|COI-5P
63
BNSC521-14|MT05407|Liocarcinus_navigator|COI-5P
43
BNSC520-14|MT05406|Liocarcinus_navigator|COI-5P
14
BNSDE320-13|MT04676|Necora_puber|COI-5P
BNSDE126-12|MT04556|Thia_scutellata|COI-5P
49
BNSDE127-12|MT04557|Thia_scutellata|COI-5P
100

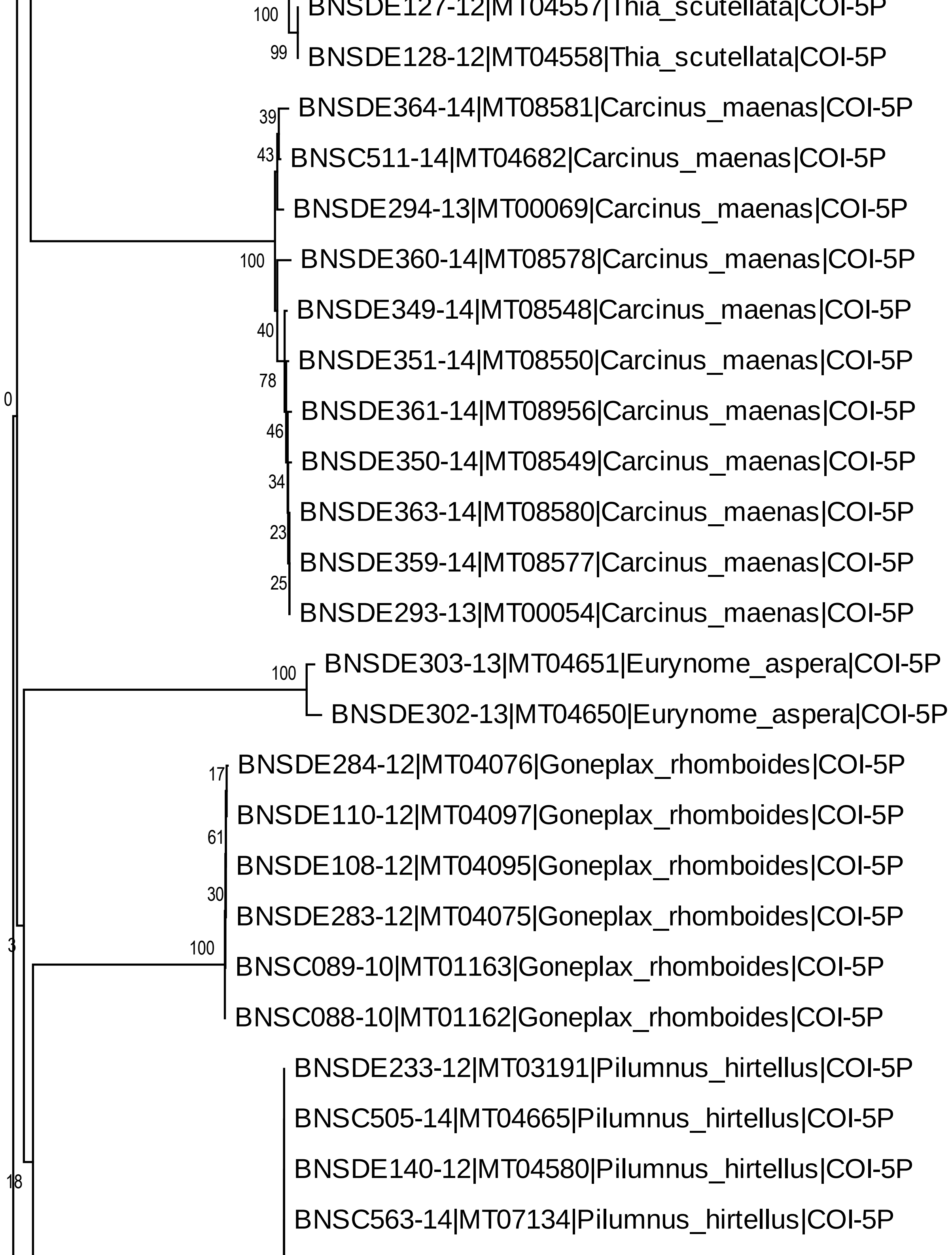
BNSDE127-12|MT04557|Thia_scutellata|COI-5P
BNSDE128-12|MT04558|Thia_scutellata|COI-5P
BNSDE364-14|MT08581|Carcinus_maenas|COI-5P
BNSC511-14|MT04682|Carcinus_maenas|COI-5P
BNSDE294-13|MT00069|Carcinus_maenas|COI-5P
BNSDE360-14|MT08578|Carcinus_maenas|COI-5P
BNSDE349-14|MT08548|Carcinus_maenas|COI-5P
BNSDE351-14|MT08550|Carcinus_maenas|COI-5P
BNSDE361-14|MT08956|Carcinus_maenas|COI-5P
BNSDE350-14|MT08549|Carcinus_maenas|COI-5P
BNSDE363-14|MT08580|Carcinus_maenas|COI-5P
BNSDE359-14|MT08577|Carcinus_maenas|COI-5P
BNSDE293-13|MT00054|Carcinus_maenas|COI-5P
BNSDE303-13|MT04651|Eurynome_aspera|COI-5P
BNSDE302-13|MT04650|Eurynome_aspera|COI-5P
BNSDE284-12|MT04076|Goneplax_rhomboides|COI-5P
BNSDE110-12|MT04097|Goneplax_rhomboides|COI-5P
BNSDE108-12|MT04095|Goneplax_rhomboides|COI-5P
BNSDE283-12|MT04075|Goneplax_rhomboides|COI-5P
BNSC089-10|MT01163|Goneplax_rhomboides|COI-5P
BNSC088-10|MT01162|Goneplax_rhomboides|COI-5P
BNSDE233-12|MT03191|Pilumnus_hirtellus|COI-5P
BNSC505-14|MT04665|Pilumnus_hirtellus|COI-5P
BNSDE140-12|MT04580|Pilumnus_hirtellus|COI-5P
BNSC563-14|MT07134|Pilumnus_hirtellus|COI-5P

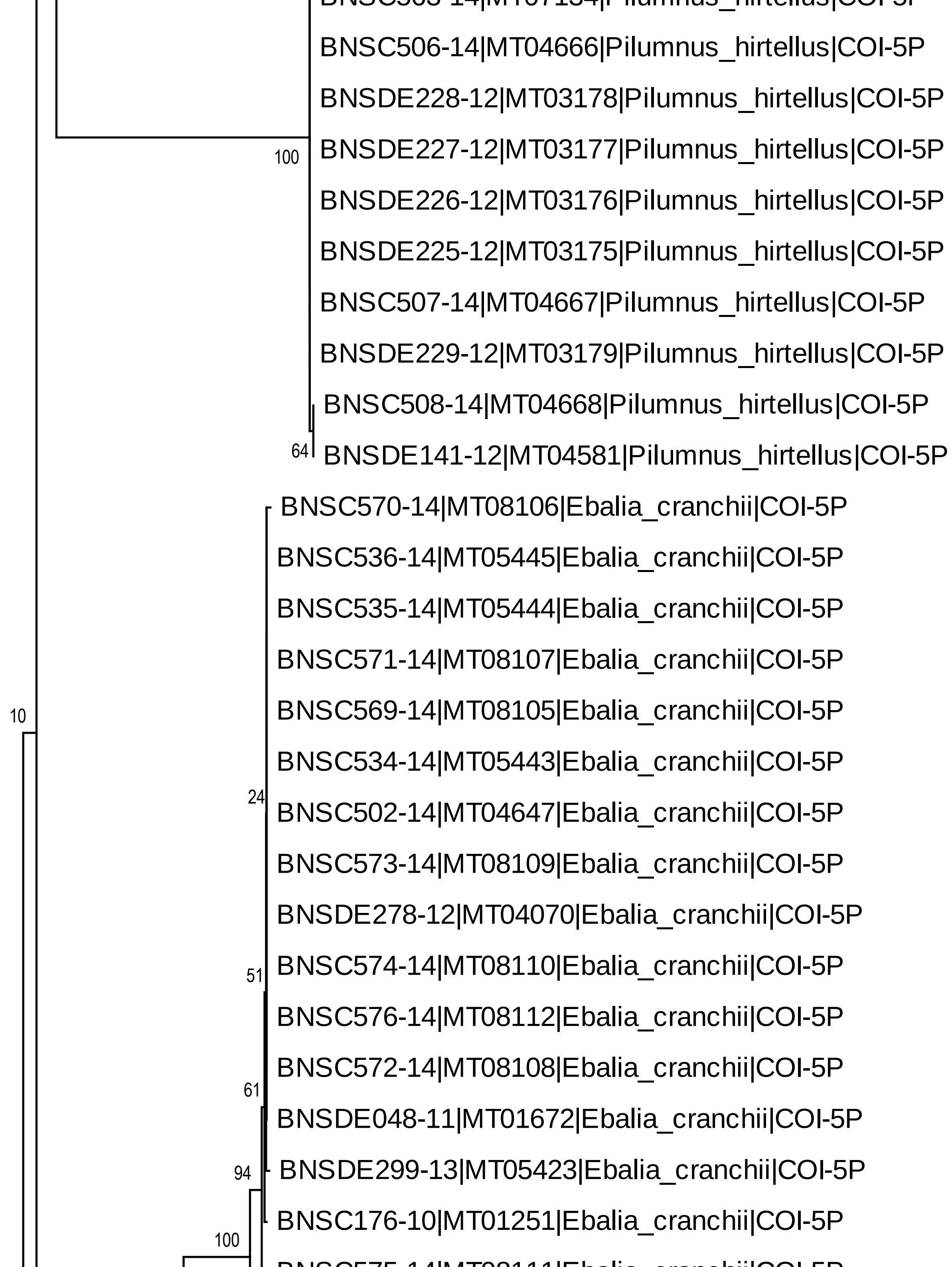

BNSC506-14|MT04666|Pilumnus_hirtellus|COI-5P
BNSDE228-12|MT03178|Pilumnus_hirtellus|COI-5P
100
BNSDE227-12|MT03177|Pilumnus_hirtellus|COI-5P
BNSDE226-12|MT03176|Pilumnus_hirtellus|COI-5P
BNSDE225-12|MT03175|Pilumnus_hirtellus|COI-5P
BNSC507-14|MT04667|Pilumnus_hirtellus|COI-5P
BNSDE229-12|MT03179|Pilumnus_hirtellus|COI-5P
BNSC508-14|MT04668|Pilumnus_hirtellus|COI-5P
64
BNSDE141-12|MT04581|Pilumnus_hirtellus|COI-5P
BNSC570-14|MT08106|Ebalia_cranchii|COI-5P
BNSC536-14|MT05445|Ebalia_cranchii|COI-5P
BNSC535-14|MT05444|Ebalia_cranchii|COI-5P
BNSC571-14|MT08107|Ebalia_cranchii|COI-5P
10
BNSC569-14|MT08105|Ebalia_cranchii|COI-5P
BNSC534-14|MT05443|Ebalia_cranchii|COI-5P
24
BNSC502-14|MT04647|Ebalia_cranchii|COI-5P
BNSC573-14|MT08109|Ebalia_cranchii|COI-5P
BNSDE278-12|MT04070|Ebalia_cranchii|COI-5P
51
BNSC574-14|MT08110|Ebalia_cranchii|COI-5P
BNSC576-14|MT08112|Ebalia_cranchii|COI-5P
BNSC572-14|MT08108|Ebalia_cranchii|COI-5P
61
BNSDE048-11|MT01672|Ebalia_cranchii|COI-5P
94
BNSDE299-13|MT05423|Ebalia_cranchii|COI-5P
BNSC176-10|MT01251|Ebalia_cranchii|COI-5P
100

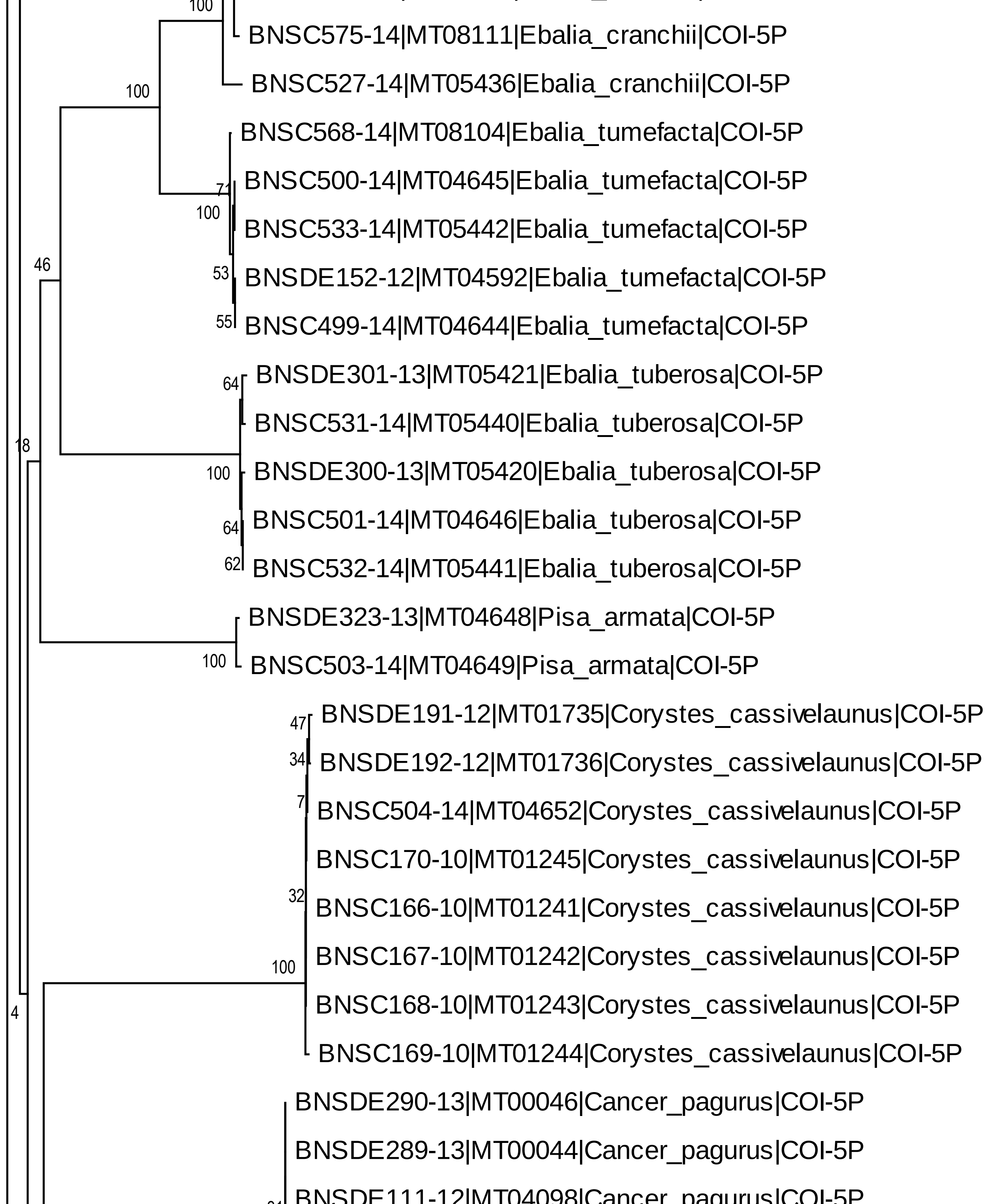
BNSC575-14|MT08111|Ebalia_cranchii|COI-5P
BNSC527-14|MT05436|Ebalia_cranchii|COI-5P
BNSC568-14|MT08104|Ebalia_tumefacta|COI-5P
BNSC500-14|MT04645|Ebalia_tumefacta|COI-5P
BNSC533-14|MT05442|Ebalia_tumefacta|COI-5P
BNSDE152-12|MT04592|Ebalia_tumefacta|COI-5P
BNSC499-14|MT04644|Ebalia_tumefacta|COI-5P
BNSDE301-13|MT05421|Ebalia_tuberosa|COI-5P
BNSC531-14|MT05440|Ebalia_tuberosa|COI-5P
BNSDE300-13|MT05420|Ebalia_tuberosa|COI-5P
BNSC501-14|MT04646|Ebalia_tuberosa|COI-5P
BNSC532-14|MT05441|Ebalia_tuberosa|COI-5P
BNSDE323-13|MT04648|Pisa_armata|COI-5P
BNSC503-14|MT04649|Pisa_armata|COI-5P
BNSDE191-12|MT01735|Corystes_cassivelaunus|COI-5P
BNSDE192-12|MT01736|Corystes_cassivelaunus|COI-5P
BNSC504-14|MT04652|Corystes_cassivelaunus|COI-5P
BNSC170-10|MT01245|Corystes_cassivelaunus|COI-5P
BNSC166-10|MT01241|Corystes_cassivelaunus|COI-5P
BNSC167-10|MT01242|Corystes_cassivelaunus|COI-5P
BNSC168-10|MT01243|Corystes_cassivelaunus|COI-5P
BNSC169-10|MT01244|Corystes_cassivelaunus|COI-5P
BNSDE290-13|MT00046|Cancer_pagurus|COI-5P
BNSDE289-13|MT00044|Cancer_pagurus|COI-5P
BNSDE111-12|MT04098|Cancer_pagurus|COI-5P

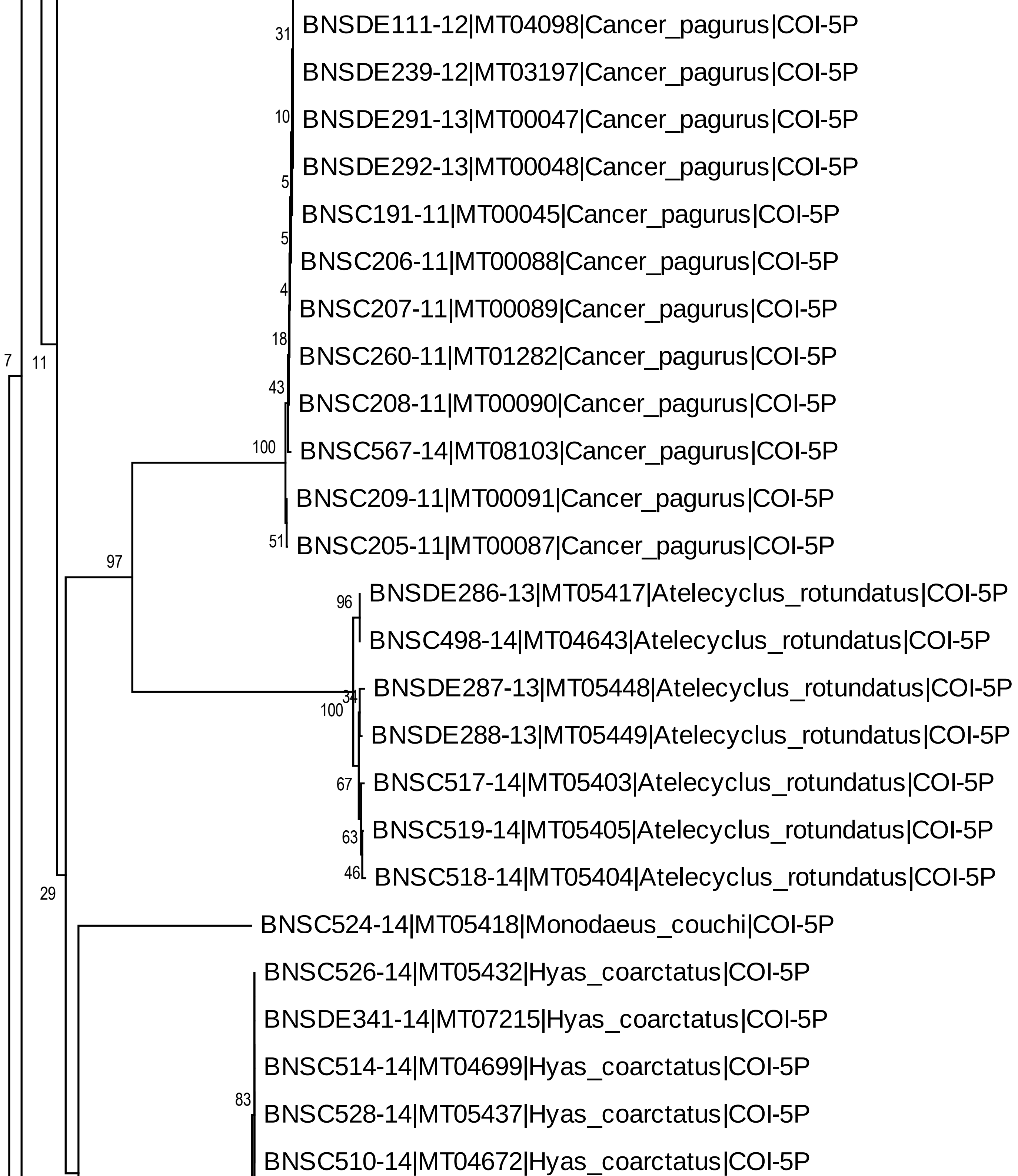

BNSDE111-12|MT04098|Cancer_pagurus|COI-5P
BNSDE239-12|MT03197|Cancer_pagurus|COI-5P
BNSDE291-13|MT00047|Cancer_pagurus|COI-5P
BNSDE292-13|MT00048|Cancer_pagurus|COI-5P
BNSC191-11|MT00045|Cancer_pagurus|COI-5P
BNSC206-11|MT00088|Cancer_pagurus|COI-5P
BNSC207-11|MT00089|Cancer_pagurus|COI-5P
BNSC260-11|MT01282|Cancer_pagurus|COI-5P
BNSC208-11|MT00090|Cancer_pagurus|COI-5P
BNSC567-14|MT08103|Cancer_pagurus|COI-5P
BNSC209-11|MT00091|Cancer_pagurus|COI-5P
BNSC205-11|MT00087|Cancer_pagurus|COI-5P
BNSDE286-13|MT05417|Atelecyclus_rotundatus|COI-5P
BNSC498-14|MT04643|Atelecyclus_rotundatus|COI-5P
BNSDE287-13|MT05448|Atelecyclus_rotundatus|COI-5P
BNSDE288-13|MT05449|Atelecyclus_rotundatus|COI-5P
BNSC517-14|MT05403|Atelecyclus_rotundatus|COI-5P
BNSC519-14|MT05405|Atelecyclus_rotundatus|COI-5P
BNSC518-14|MT05404|Atelecyclus_rotundatus|COI-5P
BNSC524-14|MT05418|Monodaeus_couchi|COI-5P
BNSC526-14|MT05432|Hyas_coarctatus|COI-5P
BNSDE341-14|MT07215|Hyas_coarctatus|COI-5P
BNSC514-14|MT04699|Hyas_coarctatus|COI-5P
BNSC528-14|MT05437|Hyas_coarctatus|COI-5P
BNSC510-14|MT04672|Hyas_coarctatus|COI-5P

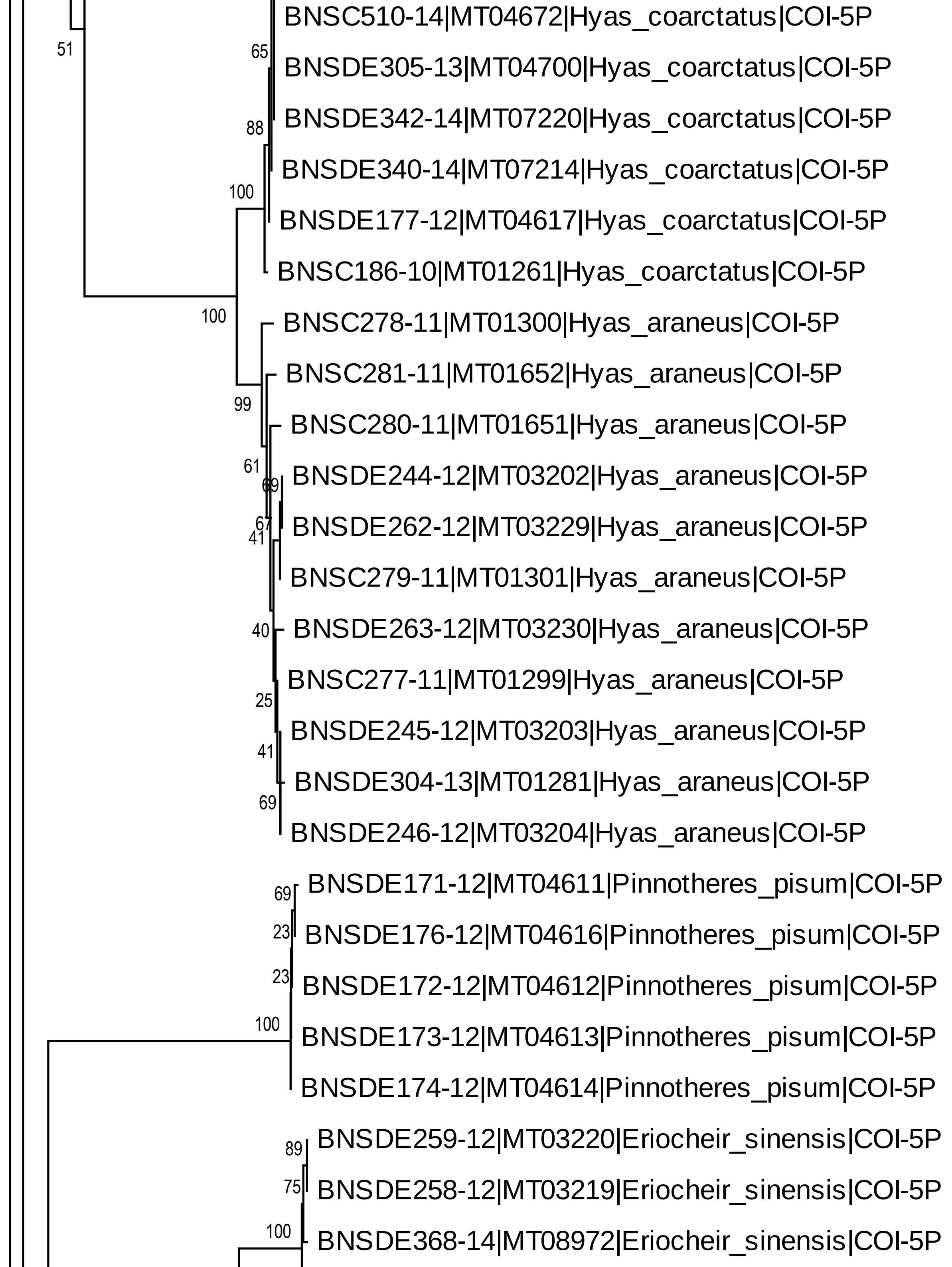
BNSC510-14|MT04672|Hyas_coarctatus|COI-5P
BNSDE305-13|MT04700|Hyas_coarctatus|COI-5P
BNSDE342-14|MT07220|Hyas_coarctatus|COI-5P
BNSDE340-14|MT07214|Hyas_coarctatus|COI-5P
BNSDE177-12|MT04617|Hyas_coarctatus|COI-5P
BNSC186-10|MT01261|Hyas_coarctatus|COI-5P
BNSC278-11|MT01300|Hyas_araneus|COI-5P
BNSC281-11|MT01652|Hyas_araneus|COI-5P
BNSC280-11|MT01651|Hyas_araneus|COI-5P
BNSDE244-12|MT03202|Hyas_araneus|COI-5P
BNSDE262-12|MT03229|Hyas_araneus|COI-5P
BNSC279-11|MT01301|Hyas_araneus|COI-5P
BNSDE263-12|MT03230|Hyas_araneus|COI-5P
BNSC277-11|MT01299|Hyas_araneus|COI-5P
BNSDE245-12|MT03203|Hyas_araneus|COI-5P
BNSDE304-13|MT01281|Hyas_araneus|COI-5P
BNSDE246-12|MT03204|Hyas_araneus|COI-5P
BNSDE171-12|MT04611|Pinnotheres_pisum|COI-5P
BNSDE176-12|MT04616|Pinnotheres_pisum|COI-5P
BNSDE172-12|MT04612|Pinnotheres_pisum|COI-5P
BNSDE173-12|MT04613|Pinnotheres_pisum|COI-5P
BNSDE174-12|MT04614|Pinnotheres_pisum|COI-5P
BNSDE259-12|MT03220|Eriocheir_sinensis|COI-5P
BNSDE258-12|MT03219|Eriocheir_sinensis|COI-5P
BNSDE368-14|MT08972|Eriocheir_sinensis|COI-5P

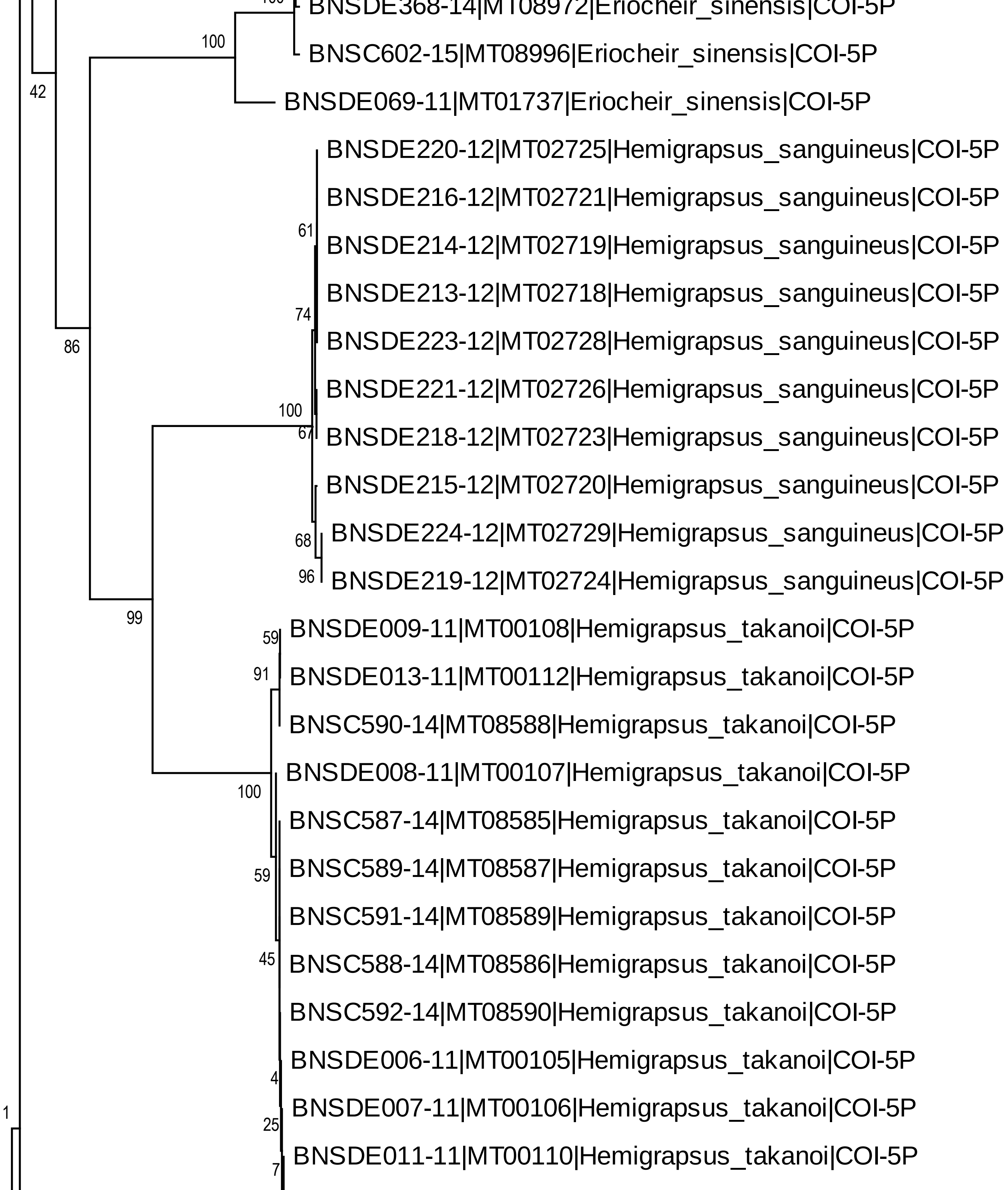
BNSDE368-14|MT08972|Eriocheir_sinensis|COI-5P
BNSC602-15|MT08996|Eriocheir_sinensis|COI-5P
BNSDE069-11|MT01737|Eriocheir_sinensis|COI-5P
BNSDE220-12|MT02725|Hemigrapsus_sanguineus|COI-5P
BNSDE216-12|MT02721|Hemigrapsus_sanguineus|COI-5P
BNSDE214-12|MT02719|Hemigrapsus_sanguineus|COI-5P
BNSDE213-12|MT02718|Hemigrapsus_sanguineus|COI-5P
BNSDE223-12|MT02728|Hemigrapsus_sanguineus|COI-5P
BNSDE221-12|MT02726|Hemigrapsus_sanguineus|COI-5P
BNSDE218-12|MT02723|Hemigrapsus_sanguineus|COI-5P
BNSDE215-12|MT02720|Hemigrapsus_sanguineus|COI-5P
BNSDE224-12|MT02729|Hemigrapsus_sanguineus|COI-5P
BNSDE219-12|MT02724|Hemigrapsus_sanguineus|COI-5P
BNSDE009-11|MT00108|Hemigrapsus_takanoi|COI-5P
BNSDE013-11|MT00112|Hemigrapsus_takanoi|COI-5P
BNSC590-14|MT08588|Hemigrapsus_takanoi|COI-5P
BNSDE008-11|MT00107|Hemigrapsus_takanoi|COI-5P
BNSC587-14|MT08585|Hemigrapsus_takanoi|COI-5P
BNSC589-14|MT08587|Hemigrapsus_takanoi|COI-5P
BNSC591-14|MT08589|Hemigrapsus_takanoi|COI-5P
BNSC588-14|MT08586|Hemigrapsus_takanoi|COI-5P
BNSC592-14|MT08590|Hemigrapsus_takanoi|COI-5P
BNSDE006-11|MT00105|Hemigrapsus_takanoi|COI-5P
BNSDE007-11|MT00106|Hemigrapsus_takanoi|COI-5P
BNSDE011-11|MT00110|Hemigrapsus_takanoi|COI-5P

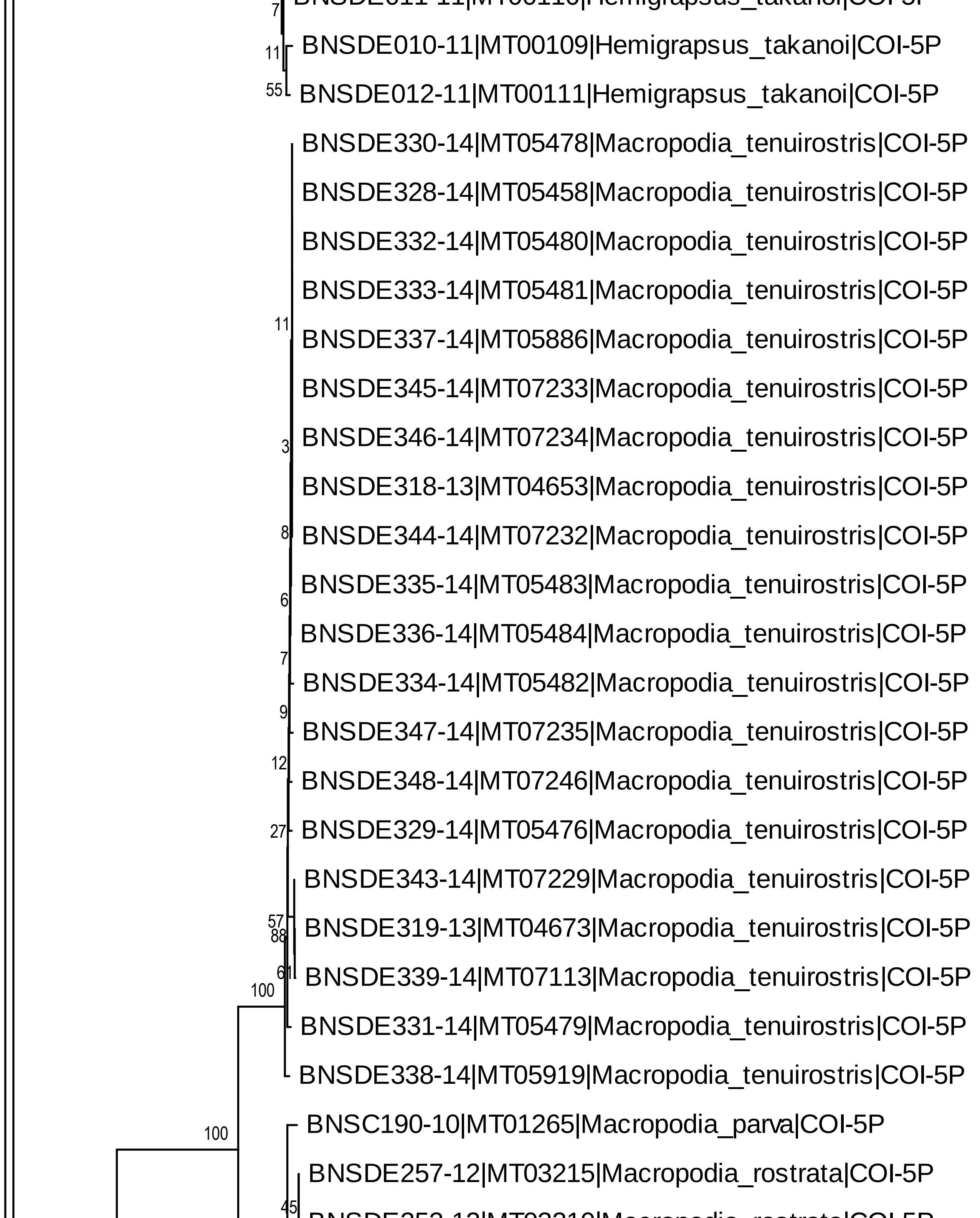

7
11
BNSDE010-11|MT00109|Hemigrapsus_takanoi|COI-5P
55
BNSDE012-11|MT00111|Hemigrapsus_takanoi|COI-5P
BNSDE330-14|MT05478|Macropodia_tenuirostris|COI-5P
BNSDE328-14|MT05458|Macropodia_tenuirostris|COI-5P
BNSDE332-14|MT05480|Macropodia_tenuirostris|COI-5P
BNSDE333-14|MT05481|Macropodia_tenuirostris|COI-5P
11
BNSDE337-14|MT05886|Macropodia_tenuirostris|COI-5P
BNSDE345-14|MT07233|Macropodia_tenuirostris|COI-5P
BNSDE346-14|MT07234|Macropodia_tenuirostris|COI-5P
3
BNSDE318-13|MT04653|Macropodia_tenuirostris|COI-5P
8
BNSDE344-14|MT07232|Macropodia_tenuirostris|COI-5P
BNSDE335-14|MT05483|Macropodia_tenuirostris|COI-5P
6
BNSDE336-14|MT05484|Macropodia_tenuirostris|COI-5P
7
BNSDE334-14|MT05482|Macropodia_tenuirostris|COI-5P
9
BNSDE347-14|MT07235|Macropodia_tenuirostris|COI-5P
12
BNSDE348-14|MT07246|Macropodia_tenuirostris|COI-5P
27
BNSDE329-14|MT05476|Macropodia_tenuirostris|COI-5P
BNSDE343-14|MT07229|Macropodia_tenuirostris|COI-5P
57
88
BNSDE319-13|MT04673|Macropodia_tenuirostris|COI-5P
61
BNSDE339-14|MT07113|Macropodia_tenuirostris|COI-5P
100
BNSDE331-14|MT05479|Macropodia_tenuirostris|COI-5P
BNSDE338-14|MT05919|Macropodia_tenuirostris|COI-5P
100
BNSC190-10|MT01265|Macropodia_parva|COI-5P
BNSDE257-12|MT03215|Macropodia_rostrata|COI-5P
45

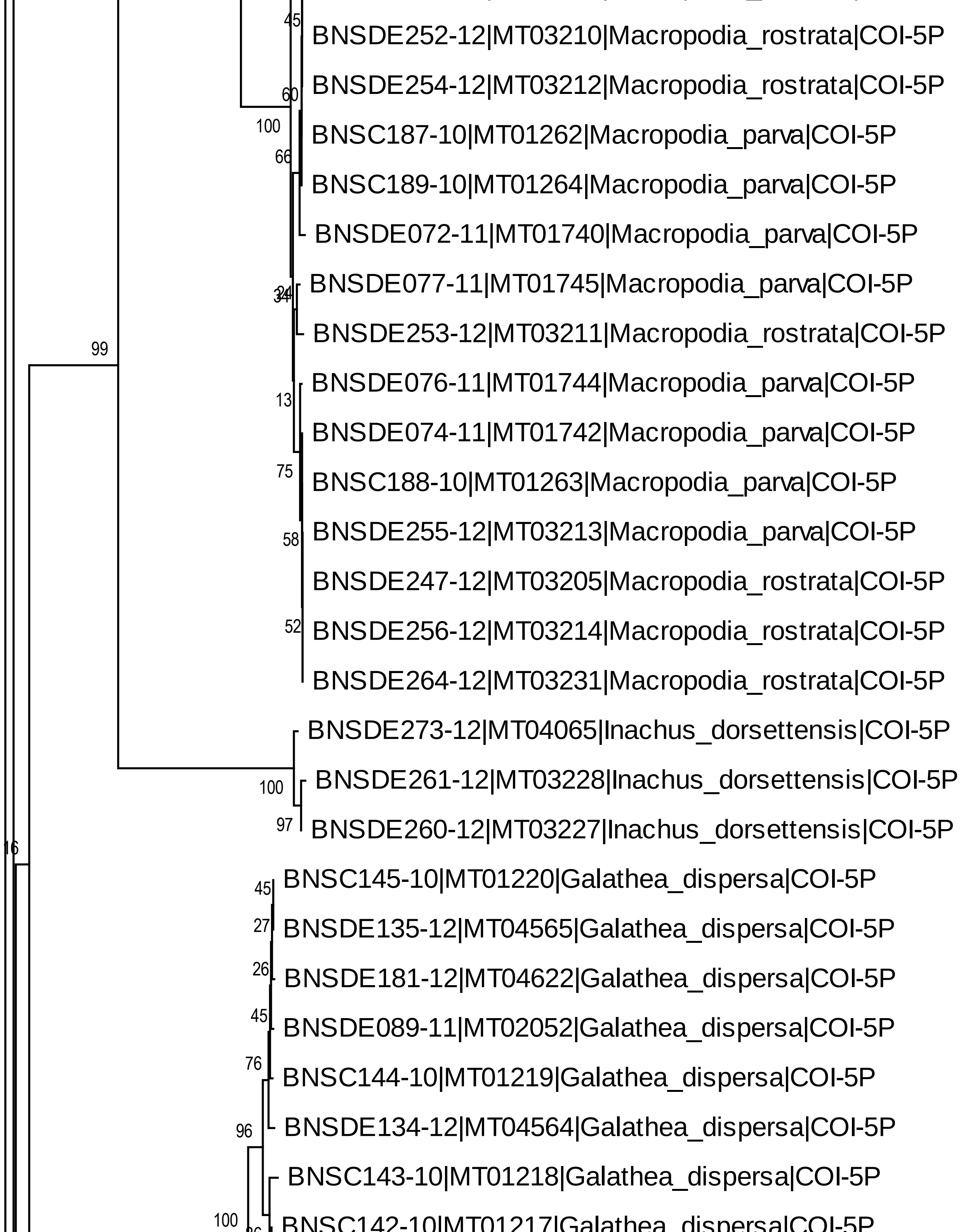
BNSDE252-12|MT03210|Macropodia_rostrata|COI-5P
BNSDE254-12|MT03212|Macropodia_rostrata|COI-5P
BNSC187-10|MT01262|Macropodia_parva|COI-5P
BNSC189-10|MT01264|Macropodia_parva|COI-5P
BNSDE072-11|MT01740|Macropodia_parva|COI-5P
BNSDE077-11|MT01745|Macropodia_parva|COI-5P
BNSDE253-12|MT03211|Macropodia_rostrata|COI-5P
BNSDE076-11|MT01744|Macropodia_parva|COI-5P
BNSDE074-11|MT01742|Macropodia_parva|COI-5P
BNSC188-10|MT01263|Macropodia_parva|COI-5P
BNSDE255-12|MT03213|Macropodia_parva|COI-5P
BNSDE247-12|MT03205|Macropodia_rostrata|COI-5P
BNSDE256-12|MT03214|Macropodia_rostrata|COI-5P
BNSDE264-12|MT03231|Macropodia_rostrata|COI-5P
BNSDE273-12|MT04065|Inachus_dorsettensis|COI-5P
BNSDE261-12|MT03228|Inachus_dorsettensis|COI-5P
BNSDE260-12|MT03227|Inachus_dorsettensis|COI-5P
BNSC145-10|MT01220|Galathea_dispersa|COI-5P
BNSDE135-12|MT04565|Galathea_dispersa|COI-5P
BNSDE181-12|MT04622|Galathea_dispersa|COI-5P
BNSDE089-11|MT02052|Galathea_dispersa|COI-5P
BNSC144-10|MT01219|Galathea_dispersa|COI-5P
BNSDE134-12|MT04564|Galathea_dispersa|COI-5P
BNSC143-10|MT01218|Galathea_dispersa|COI-5P

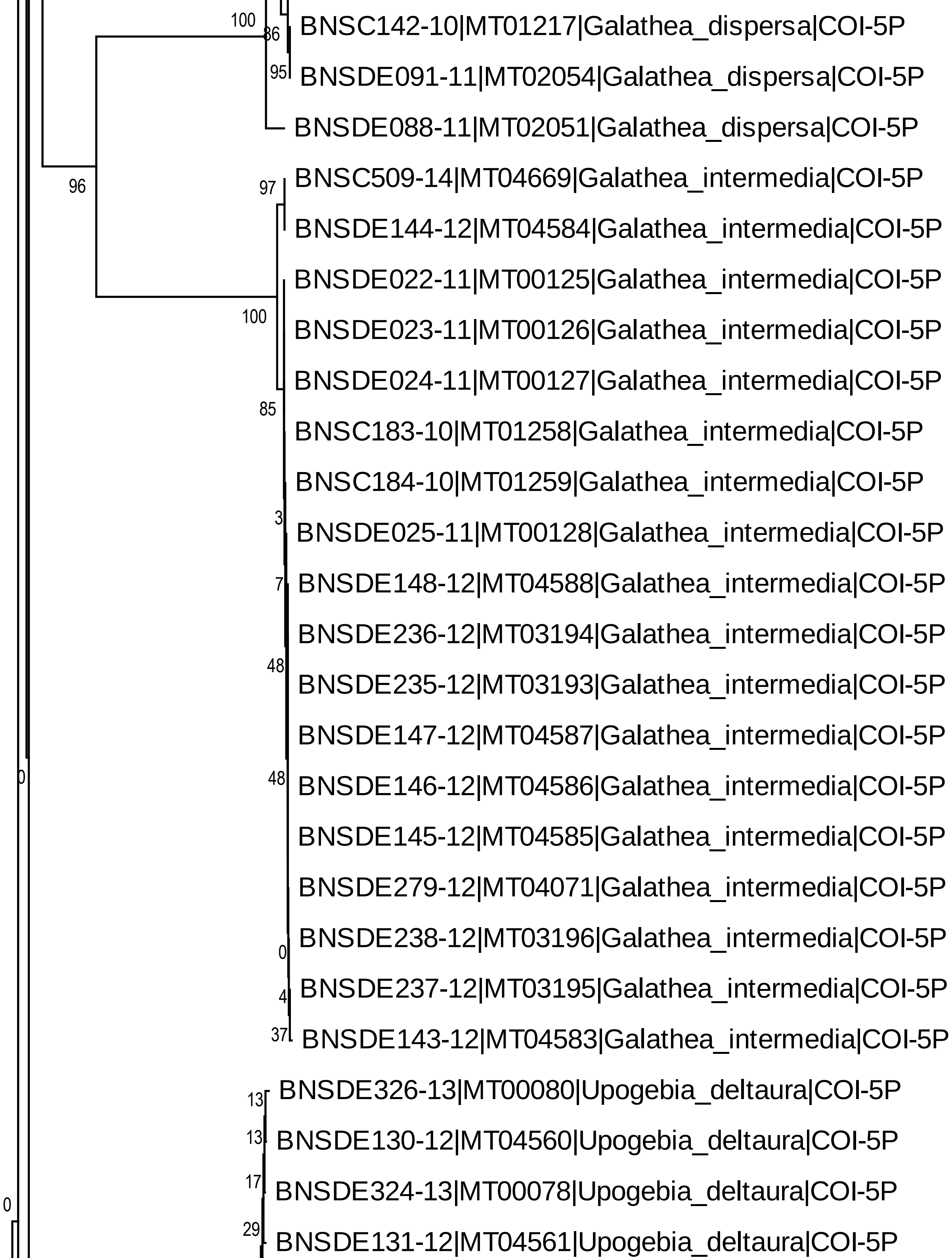
BNSC142-10|MT01217|Galathea_dispersa|COI-5P
BNSDE091-11|MT02054|Galathea_dispersa|COI-5P
BNSDE088-11|MT02051|Galathea_dispersa|COI-5P
BNSC509-14|MT04669|Galathea_intermedia|COI-5P
BNSDE144-12|MT04584|Galathea_intermedia|COI-5P
BNSDE022-11|MT00125|Galathea_intermedia|COI-5P
BNSDE023-11|MT00126|Galathea_intermedia|COI-5P
BNSDE024-11|MT00127|Galathea_intermedia|COI-5P
BNSC183-10|MT01258|Galathea_intermedia|COI-5P
BNSC184-10|MT01259|Galathea_intermedia|COI-5P
BNSDE025-11|MT00128|Galathea_intermedia|COI-5P
BNSDE148-12|MT04588|Galathea_intermedia|COI-5P
BNSDE236-12|MT03194|Galathea_intermedia|COI-5P
BNSDE235-12|MT03193|Galathea_intermedia|COI-5P
BNSDE147-12|MT04587|Galathea_intermedia|COI-5P
BNSDE146-12|MT04586|Galathea_intermedia|COI-5P
BNSDE145-12|MT04585|Galathea_intermedia|COI-5P
BNSDE279-12|MT04071|Galathea_intermedia|COI-5P
BNSDE238-12|MT03196|Galathea_intermedia|COI-5P
BNSDE237-12|MT03195|Galathea_intermedia|COI-5P
BNSDE143-12|MT04583|Galathea_intermedia|COI-5P
BNSDE326-13|MT00080|Upogebia_deltaura|COI-5P
BNSDE130-12|MT04560|Upogebia_deltaura|COI-5P
BNSDE324-13|MT00078|Upogebia_deltaura|COI-5P
BNSDE131-12|MT04561|Upogebia_deltaura|COI-5P

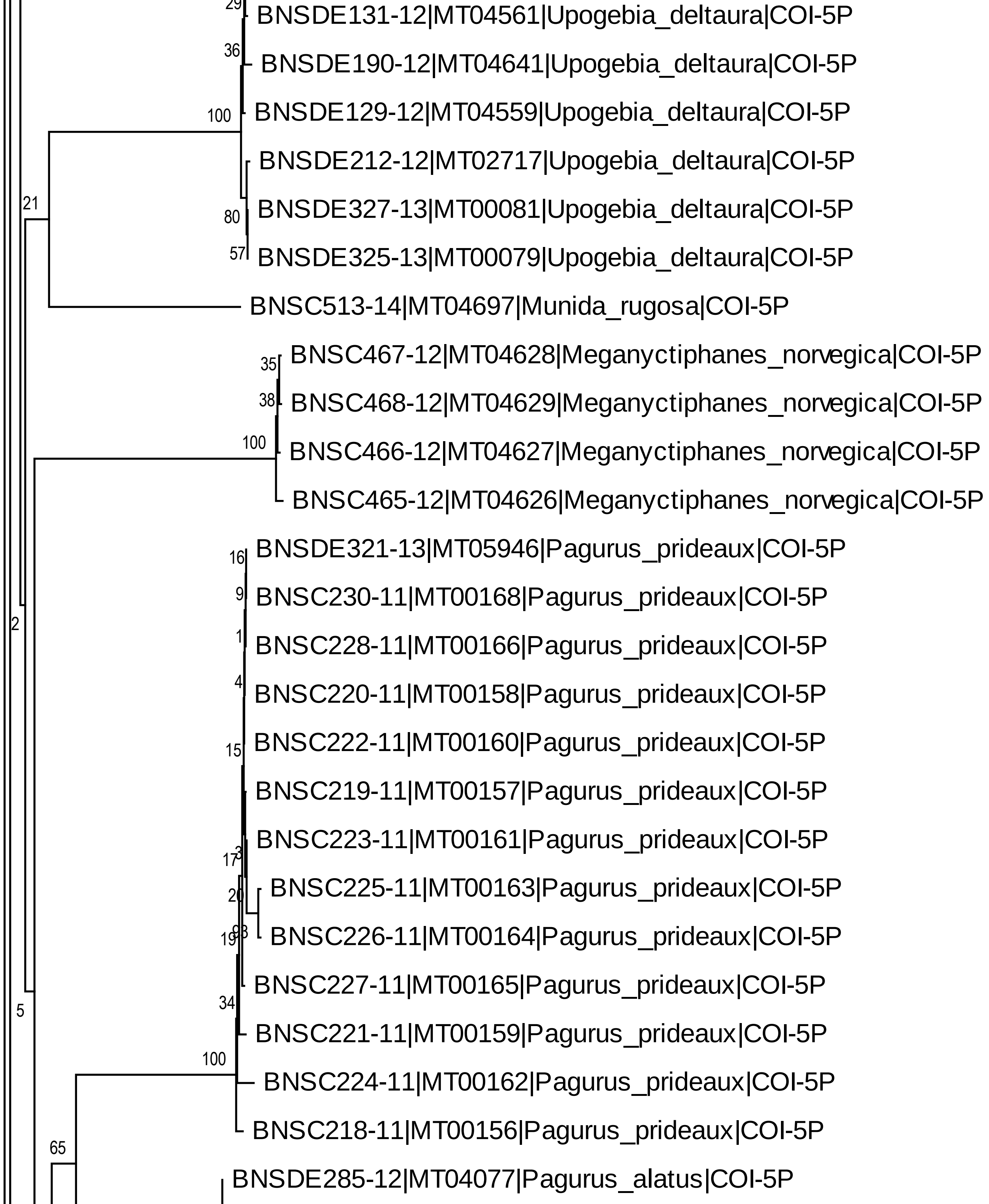
BNSDE131-12|MT04561|Upogebia_deltaura|COI-5P
BNSDE190-12|MT04641|Upogebia_deltaura|COI-5P
BNSDE129-12|MT04559|Upogebia_deltaura|COI-5P
BNSDE212-12|MT02717|Upogebia_deltaura|COI-5P
BNSDE327-13|MT00081|Upogebia_deltaura|COI-5P
BNSDE325-13|MT00079|Upogebia_deltaura|COI-5P
BNSC513-14|MT04697|Munida_rugosa|COI-5P
BNSC467-12|MT04628|Meganyctiphanes_norvegica|COI-5P
BNSC468-12|MT04629|Meganyctiphanes_norvegica|COI-5P
BNSC466-12|MT04627|Meganyctiphanes_norvegica|COI-5P
BNSC465-12|MT04626|Meganyctiphanes_norvegica|COI-5P
BNSDE321-13|MT05946|Pagurus_prideaux|COI-5P
BNSC230-11|MT00168|Pagurus_prideaux|COI-5P
BNSC228-11|MT00166|Pagurus_prideaux|COI-5P
BNSC220-11|MT00158|Pagurus_prideaux|COI-5P
BNSC222-11|MT00160|Pagurus_prideaux|COI-5P
BNSC219-11|MT00157|Pagurus_prideaux|COI-5P
BNSC223-11|MT00161|Pagurus_prideaux|COI-5P
BNSC225-11|MT00163|Pagurus_prideaux|COI-5P
BNSC226-11|MT00164|Pagurus_prideaux|COI-5P
BNSC227-11|MT00165|Pagurus_prideaux|COI-5P
BNSC221-11|MT00159|Pagurus_prideaux|COI-5P
BNSC224-11|MT00162|Pagurus_prideaux|COI-5P
BNSC218-11|MT00156|Pagurus_prideaux|COI-5P
BNSDE285-12|MT04077|Pagurus_alatus|COI-5P

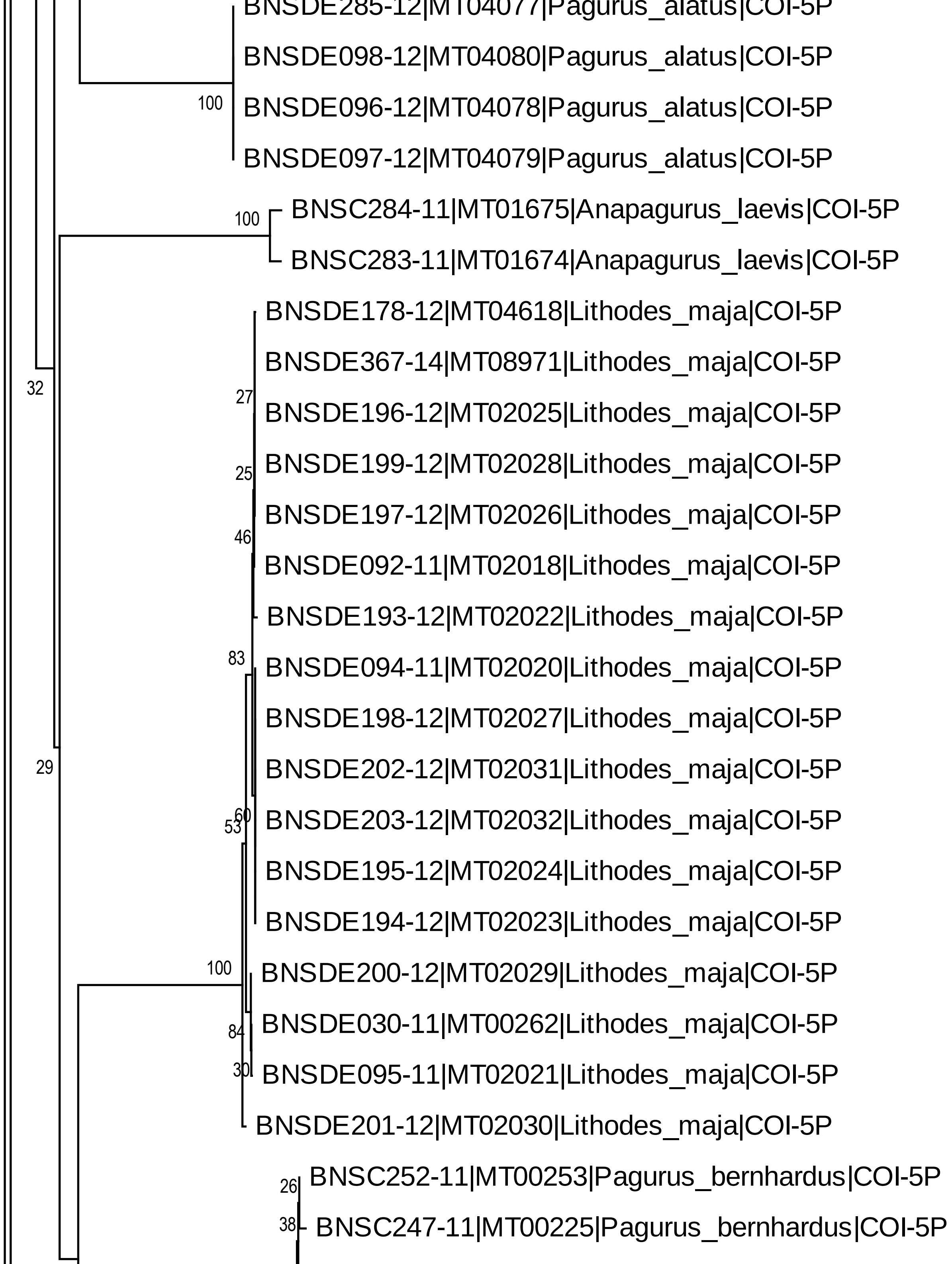
BNSDE285-12|MT04077|Pagurus_alatus|COI-5P
BNSDE098-12|MT04080|Pagurus_alatus|COI-5P
100
BNSDE096-12|MT04078|Pagurus_alatus|COI-5P
BNSDE097-12|MT04079|Pagurus_alatus|COI-5P
100
BNSC284-11|MT01675|Anapagurus_laevis|COI-5P
BNSC283-11|MT01674|Anapagurus_laevis|COI-5P
BNSDE178-12|MT04618|Lithodes_maja|COI-5P
BNSDE367-14|MT08971|Lithodes_maja|COI-5P
32
27
BNSDE196-12|MT02025|Lithodes_maja|COI-5P
BNSDE199-12|MT02028|Lithodes_maja|COI-5P
25
BNSDE197-12|MT02026|Lithodes_maja|COI-5P
46
BNSDE092-11|MT02018|Lithodes_maja|COI-5P
BNSDE193-12|MT02022|Lithodes_maja|COI-5P
83
BNSDE094-11|MT02020|Lithodes_maja|COI-5P
BNSDE198-12|MT02027|Lithodes_maja|COI-5P
29
BNSDE202-12|MT02031|Lithodes_maja|COI-5P
60
53
BNSDE203-12|MT02032|Lithodes_maja|COI-5P
BNSDE195-12|MT02024|Lithodes_maja|COI-5P
BNSDE194-12|MT02023|Lithodes_maja|COI-5P
100
BNSDE200-12|MT02029|Lithodes_maja|COI-5P
BNSDE030-11|MT00262|Lithodes_maja|COI-5P
84
30
BNSDE095-11|MT02021|Lithodes_maja|COI-5P
BNSDE201-12|MT02030|Lithodes_maja|COI-5P
26
BNSC252-11|MT00253|Pagurus_bernhardus|COI-5P
38
BNSC247-11|MT00225|Pagurus_bernhardus|COI-5P

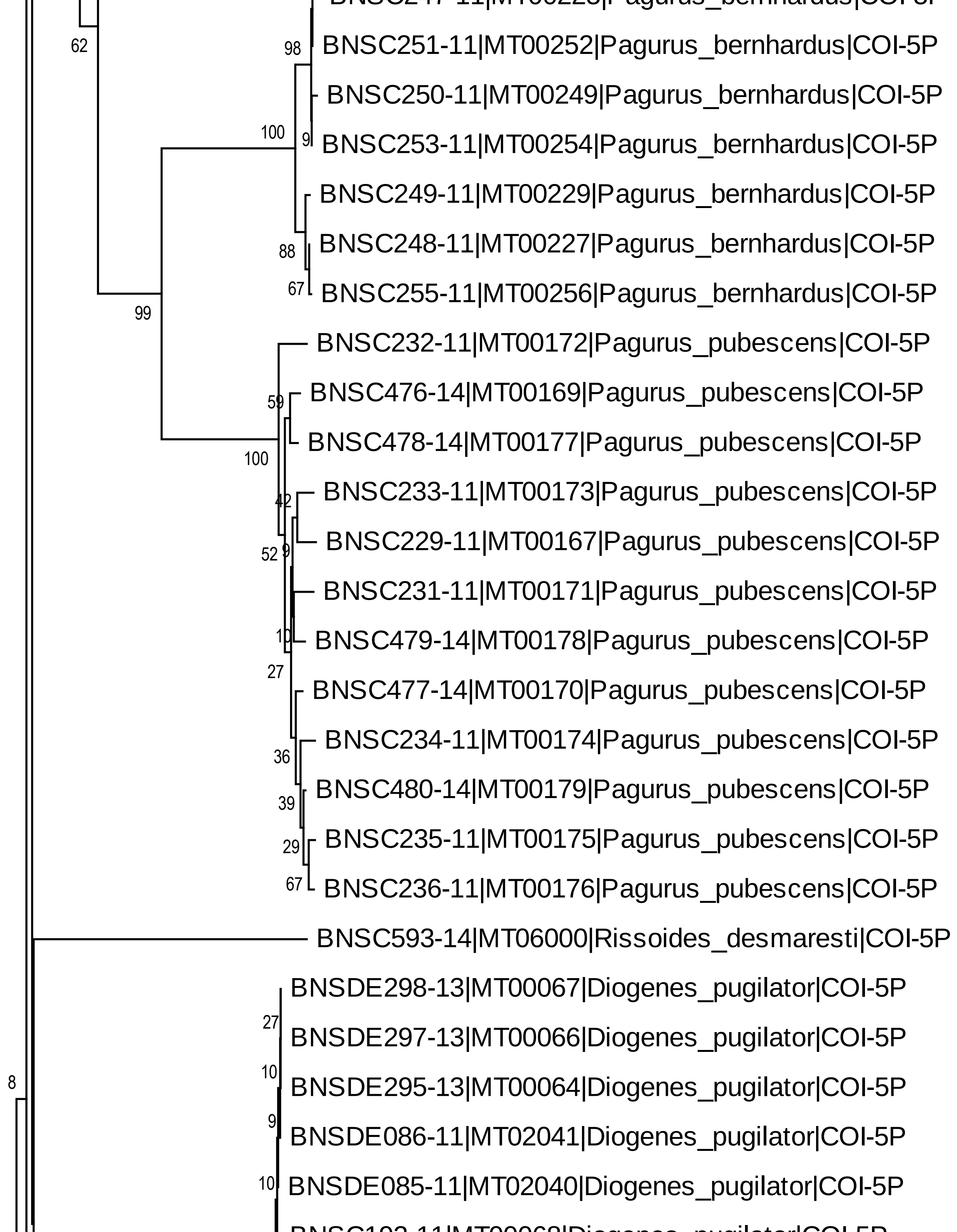

62
98
BNSC251-11|MT00252|Pagurus_bernhardus|COI-5P
BNSC250-11|MT00249|Pagurus_bernhardus|COI-5P
100
9
BNSC253-11|MT00254|Pagurus_bernhardus|COI-5P
BNSC249-11|MT00229|Pagurus_bernhardus|COI-5P
88
BNSC248-11|MT00227|Pagurus_bernhardus|COI-5P
67
BNSC255-11|MT00256|Pagurus_bernhardus|COI-5P
99
BNSC232-11|MT00172|Pagurus_pubescens|COI-5P
59
BNSC476-14|MT00169|Pagurus_pubescens|COI-5P
BNSC478-14|MT00177|Pagurus_pubescens|COI-5P
100
42
BNSC233-11|MT00173|Pagurus_pubescens|COI-5P
52
9
BNSC229-11|MT00167|Pagurus_pubescens|COI-5P
BNSC231-11|MT00171|Pagurus_pubescens|COI-5P
10
BNSC479-14|MT00178|Pagurus_pubescens|COI-5P
27
BNSC477-14|MT00170|Pagurus_pubescens|COI-5P
BNSC234-11|MT00174|Pagurus_pubescens|COI-5P
36
BNSC480-14|MT00179|Pagurus_pubescens|COI-5P
39
29
BNSC235-11|MT00175|Pagurus_pubescens|COI-5P
67
BNSC236-11|MT00176|Pagurus_pubescens|COI-5P
BNSC593-14|MT06000|Rissoides_desmaresti|COI-5P
BNSDE298-13|MT00067|Diogenes_pugilator|COI-5P
27
BNSDE297-13|MT00066|Diogenes_pugilator|COI-5P
10
BNSDE295-13|MT00064|Diogenes_pugilator|COI-5P
8
9
BNSDE086-11|MT02041|Diogenes_pugilator|COI-5P
10
BNSDE085-11|MT02040|Diogenes_pugilator|COI-5P

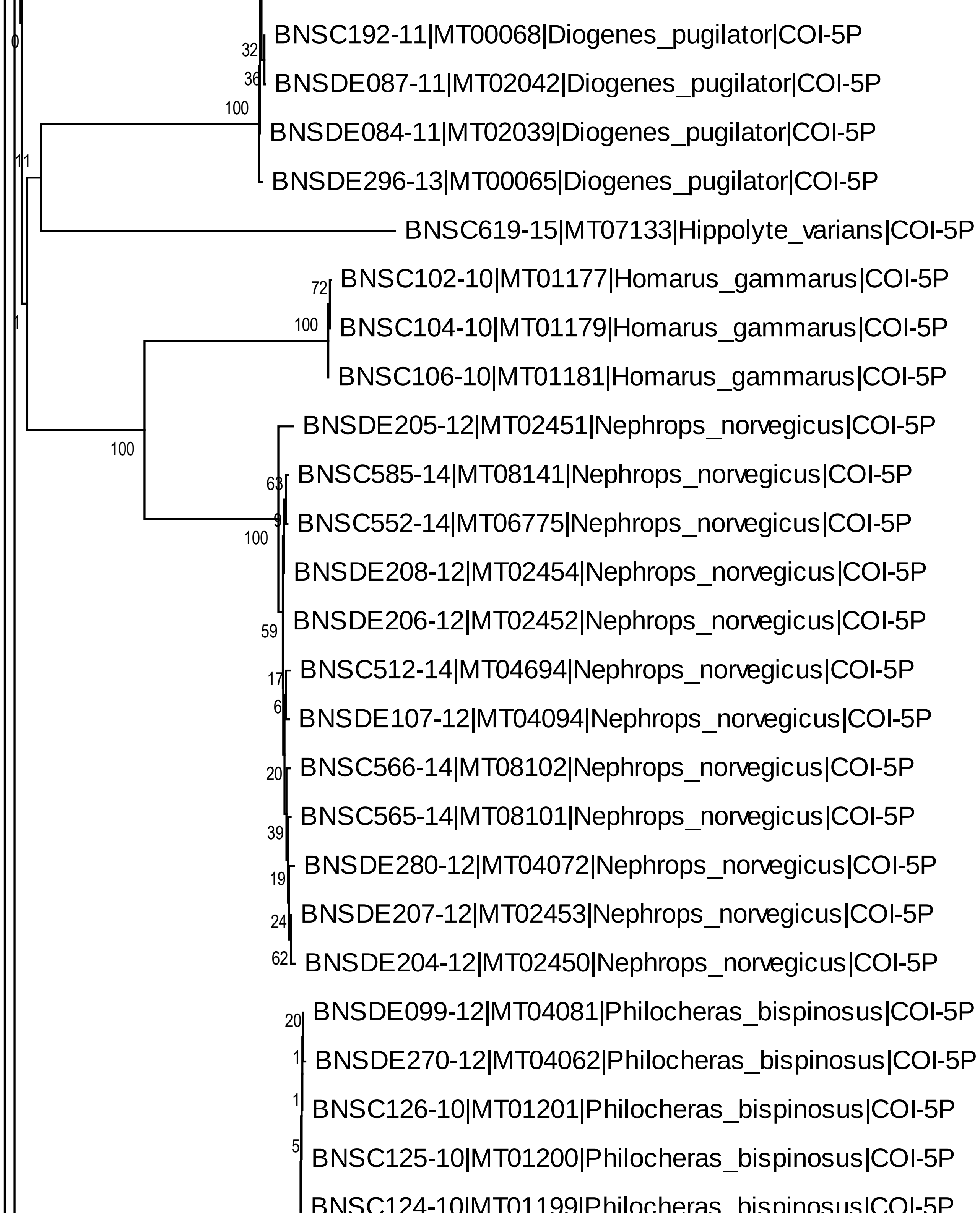
BNSC192-11|MT00068|Diogenes_pugilator|COI-5P
BNSDE087-11|MT02042|Diogenes_pugilator|COI-5P
BNSDE084-11|MT02039|Diogenes_pugilator|COI-5P
BNSDE296-13|MT00065|Diogenes_pugilator|COI-5P
BNSC619-15|MT07133|Hippolyte_varians|COI-5P
BNSC102-10|MT01177|Homarus_gammarus|COI-5P
BNSC104-10|MT01179|Homarus_gammarus|COI-5P
BNSC106-10|MT01181|Homarus_gammarus|COI-5P
BNSDE205-12|MT02451|Nephrops_norvegicus|COI-5P
BNSC585-14|MT08141|Nephrops_norvegicus|COI-5P
BNSC552-14|MT06775|Nephrops_norvegicus|COI-5P
BNSDE208-12|MT02454|Nephrops_norvegicus|COI-5P
BNSDE206-12|MT02452|Nephrops_norvegicus|COI-5P
BNSC512-14|MT04694|Nephrops_norvegicus|COI-5P
BNSDE107-12|MT04094|Nephrops_norvegicus|COI-5P
BNSC566-14|MT08102|Nephrops_norvegicus|COI-5P
BNSC565-14|MT08101|Nephrops_norvegicus|COI-5P
BNSDE280-12|MT04072|Nephrops_norvegicus|COI-5P
BNSDE207-12|MT02453|Nephrops_norvegicus|COI-5P
BNSDE204-12|MT02450|Nephrops_norvegicus|COI-5P
BNSDE099-12|MT04081|Philocheras_bispinosus|COI-5P
BNSDE270-12|MT04062|Philocheras_bispinosus|COI-5P
BNSC126-10|MT01201|Philocheras_bispinosus|COI-5P
BNSC125-10|MT01200|Philocheras_bispinosus|COI-5P

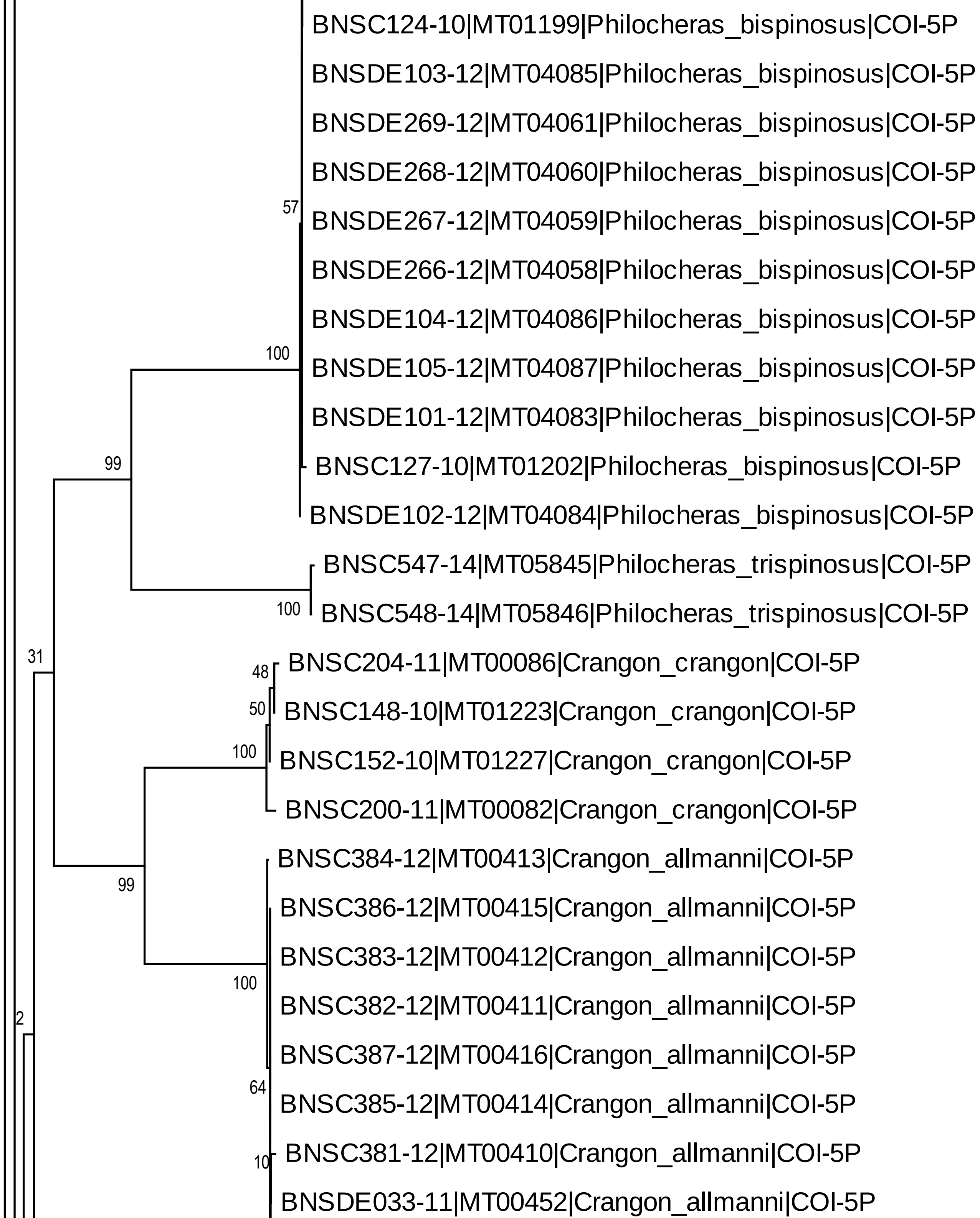
BNSC124-10|MT01199|Philocheras_bispinosus|COI-5P
BNSDE103-12|MT04085|Philocheras_bispinosus|COI-5P
BNSDE269-12|MT04061|Philocheras_bispinosus|COI-5P
BNSDE268-12|MT04060|Philocheras_bispinosus|COI-5P
57
BNSDE267-12|MT04059|Philocheras_bispinosus|COI-5P
BNSDE266-12|MT04058|Philocheras_bispinosus|COI-5P
BNSDE104-12|MT04086|Philocheras_bispinosus|COI-5P
100
BNSDE105-12|MT04087|Philocheras_bispinosus|COI-5P
BNSDE101-12|MT04083|Philocheras_bispinosus|COI-5P
99
BNSC127-10|MT01202|Philocheras_bispinosus|COI-5P
BNSDE102-12|MT04084|Philocheras_bispinosus|COI-5P
BNSC547-14|MT05845|Philocheras_trispinosus|COI-5P
100
BNSC548-14|MT05846|Philocheras_trispinosus|COI-5P
31
48
BNSC204-11|MT00086|Crangon_crangon|COI-5P
50
BNSC148-10|MT01223|Crangon_crangon|COI-5P
100
BNSC152-10|MT01227|Crangon_crangon|COI-5P
BNSC200-11|MT00082|Crangon_crangon|COI-5P
BNSC384-12|MT00413|Crangon_allmanni|COI-5P
99
BNSC386-12|MT00415|Crangon_allmanni|COI-5P
BNSC383-12|MT00412|Crangon_allmanni|COI-5P
100
BNSC382-12|MT00411|Crangon_allmanni|COI-5P
2
BNSC387-12|MT00416|Crangon_allmanni|COI-5P
64
BNSC385-12|MT00414|Crangon_allmanni|COI-5P
BNSC381-12|MT00410|Crangon_allmanni|COI-5P
10
BNSDE033-11|MT00452|Crangon_allmanni|COI-5P

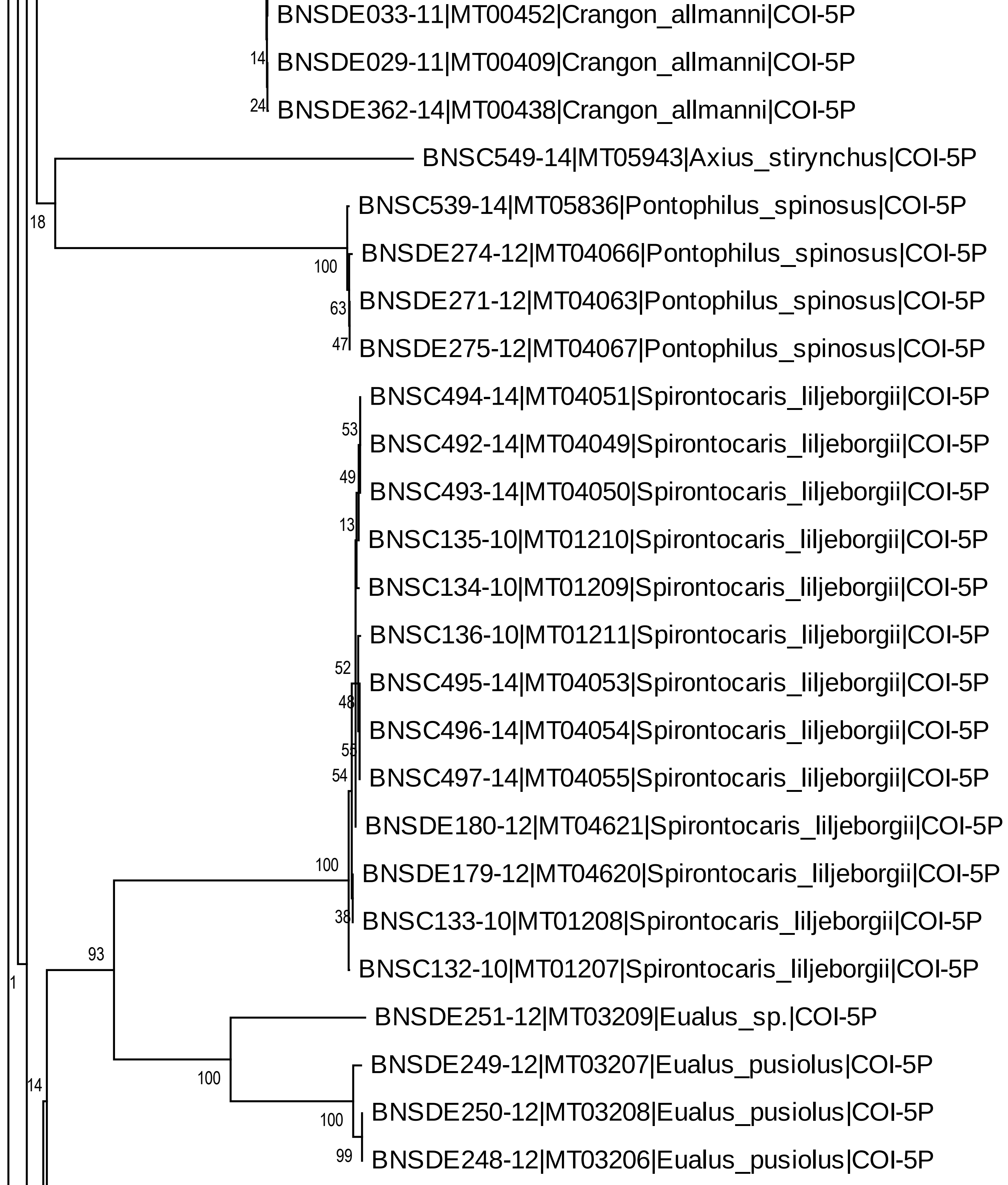
BNSDE033-11|MT00452|Crangon_allmanni|COI-5P
BNSDE029-11|MT00409|Crangon_allmanni|COI-5P
BNSDE362-14|MT00438|Crangon_allmanni|COI-5P
BNSC549-14|MT05943|Axius_stirynchus|COI-5P
BNSC539-14|MT05836|Pontophilus_spinosus|COI-5P
BNSDE274-12|MT04066|Pontophilus_spinosus|COI-5P
BNSDE271-12|MT04063|Pontophilus_spinosus|COI-5P
BNSDE275-12|MT04067|Pontophilus_spinosus|COI-5P
BNSC494-14|MT04051|Spirontocaris_liljeborgii|COI-5P
BNSC492-14|MT04049|Spirontocaris_liljeborgii|COI-5P
BNSC493-14|MT04050|Spirontocaris_liljeborgii|COI-5P
BNSC135-10|MT01210|Spirontocaris_liljeborgii|COI-5P
BNSC134-10|MT01209|Spirontocaris_liljeborgii|COI-5P
BNSC136-10|MT01211|Spirontocaris_liljeborgii|COI-5P
BNSC495-14|MT04053|Spirontocaris_liljeborgii|COI-5P
BNSC496-14|MT04054|Spirontocaris_liljeborgii|COI-5P
BNSC497-14|MT04055|Spirontocaris_liljeborgii|COI-5P
BNSDE180-12|MT04621|Spirontocaris_liljeborgii|COI-5P
BNSDE179-12|MT04620|Spirontocaris_liljeborgii|COI-5P
BNSC133-10|MT01208|Spirontocaris_liljeborgii|COI-5P
BNSC132-10|MT01207|Spirontocaris_liljeborgii|COI-5P
BNSDE251-12|MT03209|Eualus_sp.|COI-5P
BNSDE249-12|MT03207|Eualus_pusiolus|COI-5P
BNSDE250-12|MT03208|Eualus_pusiolus|COI-5P
BNSDE248-12|MT03206|Eualus_pusiolus|COI-5P

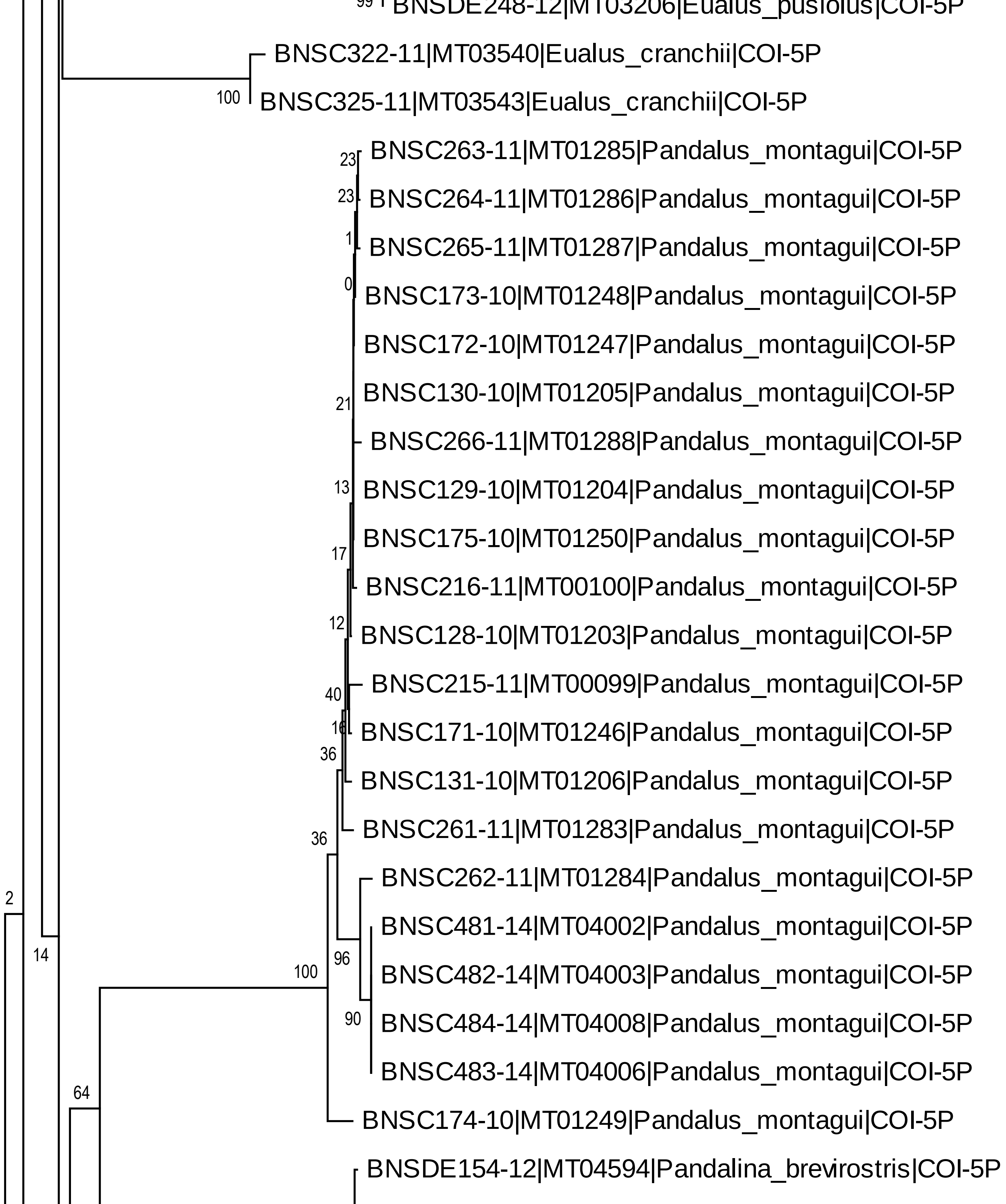
BNSC322-11|MT03540|Eualus_cranchii|COI-5P
BNSC325-11|MT03543|Eualus_cranchii|COI-5P
BNSC263-11|MT01285|Pandalus_montagui|COI-5P
BNSC264-11|MT01286|Pandalus_montagui|COI-5P
BNSC265-11|MT01287|Pandalus_montagui|COI-5P
BNSC173-10|MT01248|Pandalus_montagui|COI-5P
BNSC172-10|MT01247|Pandalus_montagui|COI-5P
BNSC130-10|MT01205|Pandalus_montagui|COI-5P
BNSC266-11|MT01288|Pandalus_montagui|COI-5P
BNSC129-10|MT01204|Pandalus_montagui|COI-5P
BNSC175-10|MT01250|Pandalus_montagui|COI-5P
BNSC216-11|MT00100|Pandalus_montagui|COI-5P
BNSC128-10|MT01203|Pandalus_montagui|COI-5P
BNSC215-11|MT00099|Pandalus_montagui|COI-5P
BNSC171-10|MT01246|Pandalus_montagui|COI-5P
BNSC131-10|MT01206|Pandalus_montagui|COI-5P
BNSC261-11|MT01283|Pandalus_montagui|COI-5P
BNSC262-11|MT01284|Pandalus_montagui|COI-5P
BNSC481-14|MT04002|Pandalus_montagui|COI-5P
BNSC482-14|MT04003|Pandalus_montagui|COI-5P
BNSC484-14|MT04008|Pandalus_montagui|COI-5P
BNSC483-14|MT04006|Pandalus_montagui|COI-5P
BNSC174-10|MT01249|Pandalus_montagui|COI-5P
BNSDE154-12|MT04594|Pandalina_brevirostris|COI-5P

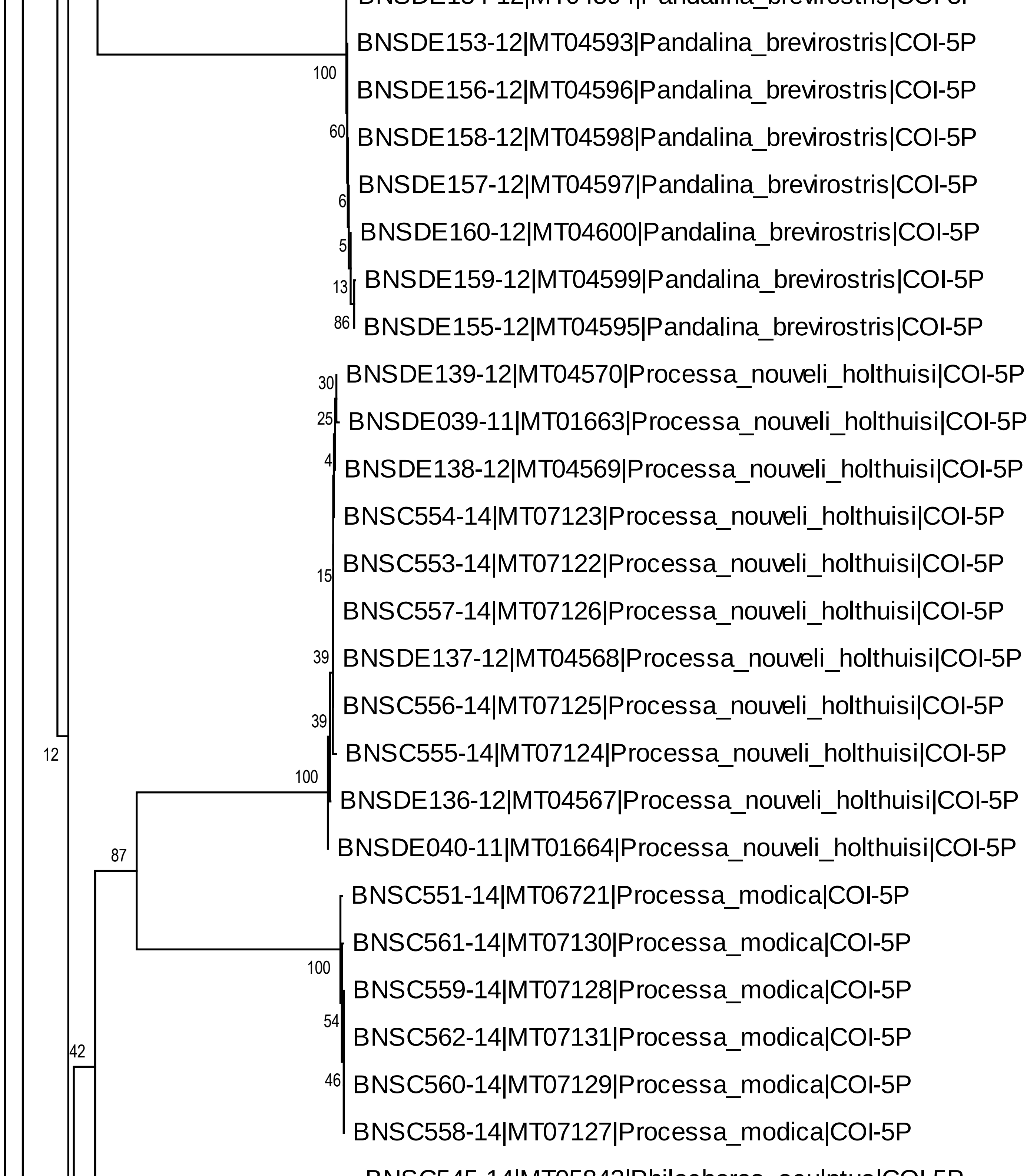
BNSDE153-12|MT04593|Pandalina_brevirostris|COI-5P
BNSDE156-12|MT04596|Pandalina_brevirostris|COI-5P
BNSDE158-12|MT04598|Pandalina_brevirostris|COI-5P
BNSDE157-12|MT04597|Pandalina_brevirostris|COI-5P
BNSDE160-12|MT04600|Pandalina_brevirostris|COI-5P
BNSDE159-12|MT04599|Pandalina_brevirostris|COI-5P
BNSDE155-12|MT04595|Pandalina_brevirostris|COI-5P
BNSDE139-12|MT04570|Processa_nouveli_holthuisi|COI-5P
BNSDE039-11|MT01663|Processa_nouveli_holthuisi|COI-5P
BNSDE138-12|MT04569|Processa_nouveli_holthuisi|COI-5P
BNSC554-14|MT07123|Processa_nouveli_holthuisi|COI-5P
BNSC553-14|MT07122|Processa_nouveli_holthuisi|COI-5P
BNSC557-14|MT07126|Processa_nouveli_holthuisi|COI-5P
BNSDE137-12|MT04568|Processa_nouveli_holthuisi|COI-5P
BNSC556-14|MT07125|Processa_nouveli_holthuisi|COI-5P
BNSC555-14|MT07124|Processa_nouveli_holthuisi|COI-5P
BNSDE136-12|MT04567|Processa_nouveli_holthuisi|COI-5P
BNSDE040-11|MT01664|Processa_nouveli_holthuisi|COI-5P
BNSC551-14|MT06721|Processa_modica|COI-5P
BNSC561-14|MT07130|Processa_modica|COI-5P
BNSC559-14|MT07128|Processa_modica|COI-5P
BNSC562-14|MT07131|Processa_modica|COI-5P
BNSC560-14|MT07129|Processa_modica|COI-5P
BNSC558-14|MT07127|Processa_modica|COI-5P
100
60
6
5
13
86
30
25
4
15
39
39
100
12
87
100
54
42
46

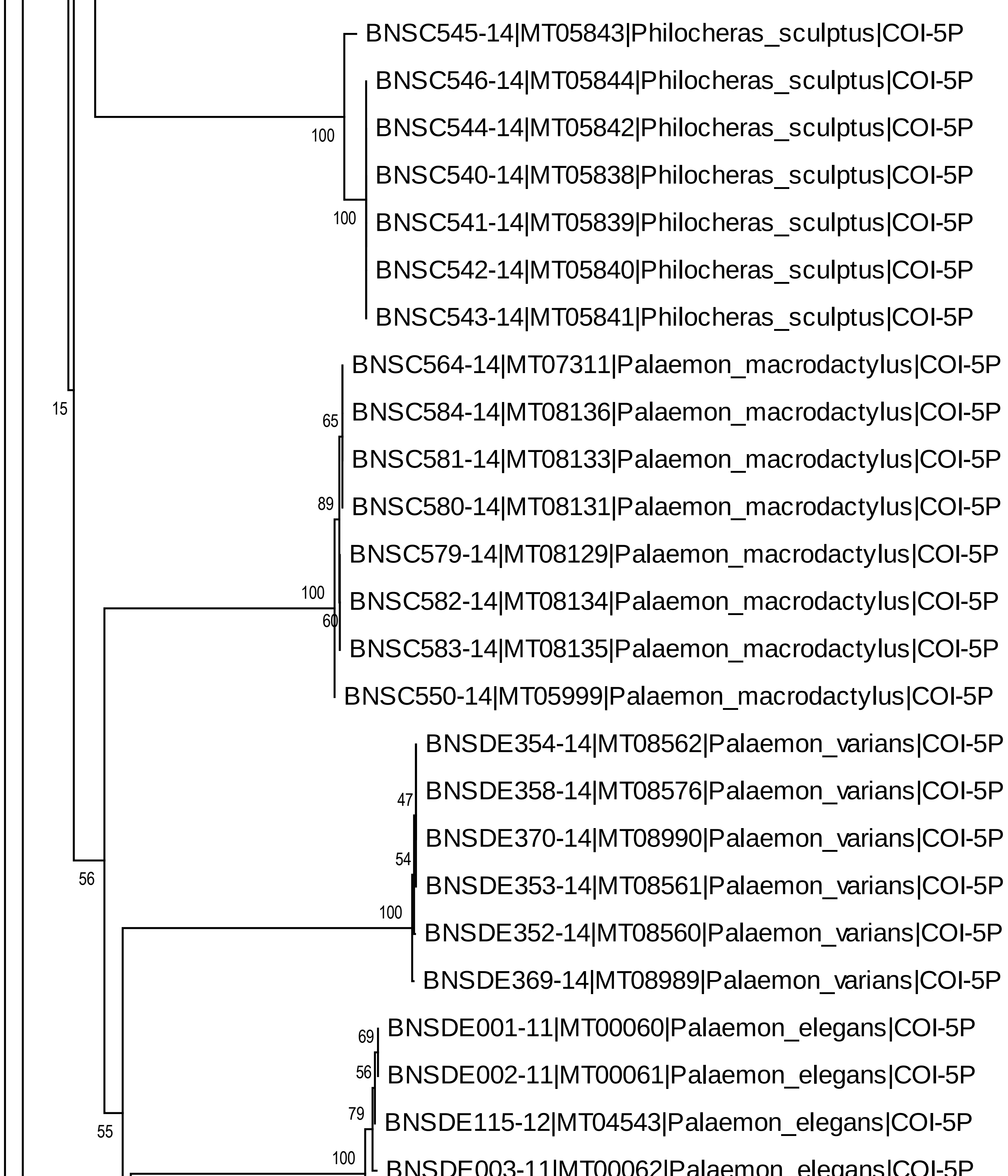
BNSC545-14|MT05843|Philocheras_sculptus|COI-5P
BNSC546-14|MT05844|Philocheras_sculptus|COI-5P
BNSC544-14|MT05842|Philocheras_sculptus|COI-5P
100
BNSC540-14|MT05838|Philocheras_sculptus|COI-5P
100
BNSC541-14|MT05839|Philocheras_sculptus|COI-5P
BNSC542-14|MT05840|Philocheras_sculptus|COI-5P
BNSC543-14|MT05841|Philocheras_sculptus|COI-5P
BNSC564-14|MT07311|Palaemon_macrodactylus|COI-5P
15
BNSC584-14|MT08136|Palaemon_macrodactylus|COI-5P
65
BNSC581-14|MT08133|Palaemon_macrodactylus|COI-5P
89
BNSC580-14|MT08131|Palaemon_macrodactylus|COI-5P
BNSC579-14|MT08129|Palaemon_macrodactylus|COI-5P
100
BNSC582-14|MT08134|Palaemon_macrodactylus|COI-5P
60
BNSC583-14|MT08135|Palaemon_macrodactylus|COI-5P
BNSC550-14|MT05999|Palaemon_macrodactylus|COI-5P
BNSDE354-14|MT08562|Palaemon_varians|COI-5P
BNSDE358-14|MT08576|Palaemon_varians|COI-5P
47
BNSDE370-14|MT08990|Palaemon_varians|COI-5P
54
56
BNSDE353-14|MT08561|Palaemon_varians|COI-5P
100
BNSDE352-14|MT08560|Palaemon_varians|COI-5P
BNSDE369-14|MT08989|Palaemon_varians|COI-5P
BNSDE001-11|MT00060|Palaemon_elegans|COI-5P
69
BNSDE002-11|MT00061|Palaemon_elegans|COI-5P
56
79
BNSDE115-12|MT04543|Palaemon_elegans|COI-5P
55
100
BNSDE003-11|MT00062|Palaemon_elegans|COI-5P

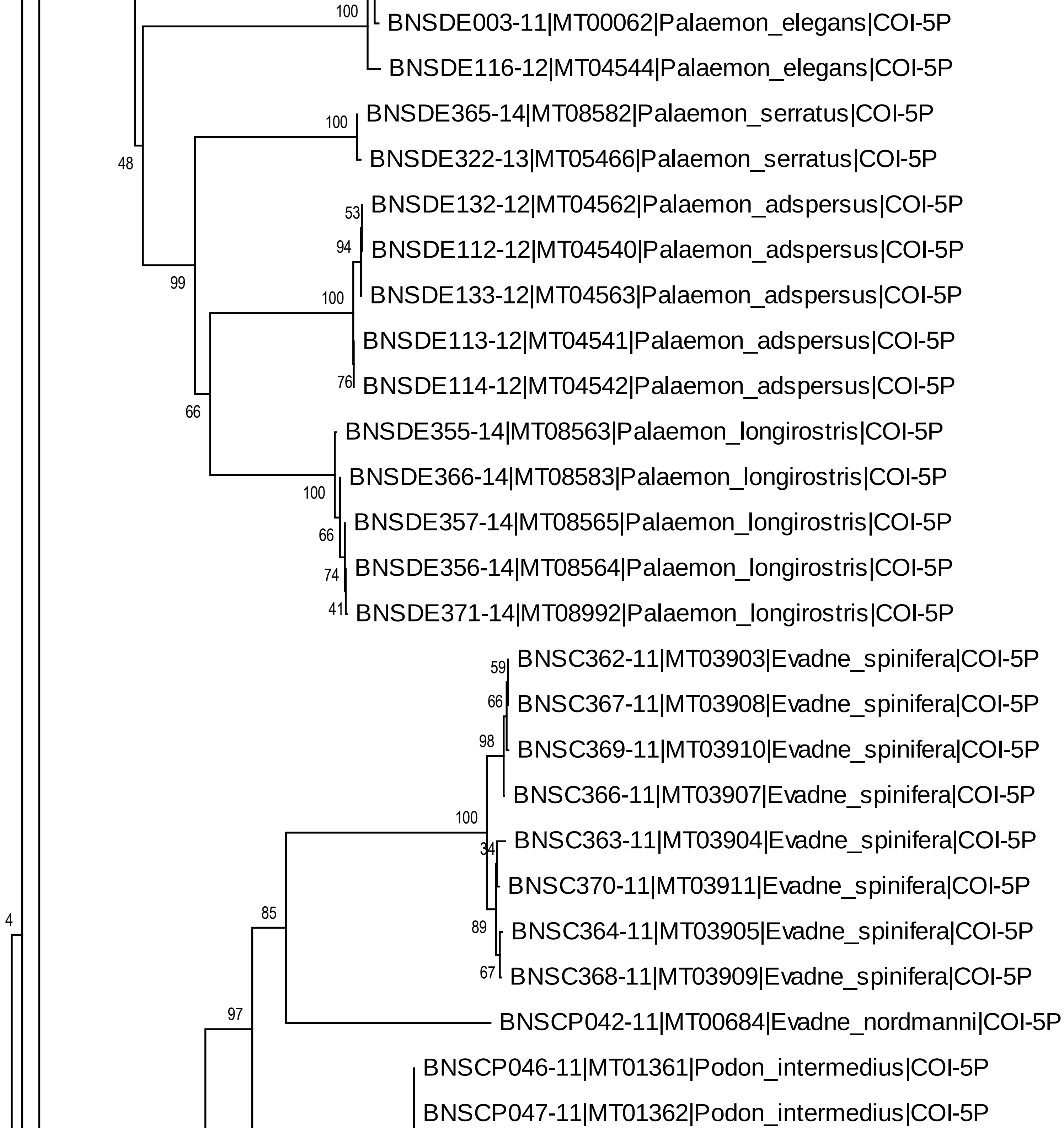
100
BNSDE003-11|MT00062|Palaemon_elegans|COI-5P
BNSDE116-12|MT04544|Palaemon_elegans|COI-5P
100
BNSDE365-14|MT08582|Palaemon_serratus|COI-5P
48
BNSDE322-13|MT05466|Palaemon_serratus|COI-5P
53
BNSDE132-12|MT04562|Palaemon_adspersus|COI-5P
94
BNSDE112-12|MT04540|Palaemon_adspersus|COI-5P
99
100
BNSDE133-12|MT04563|Palaemon_adspersus|COI-5P
BNSDE113-12|MT04541|Palaemon_adspersus|COI-5P
76
BNSDE114-12|MT04542|Palaemon_adspersus|COI-5P
66
BNSDE355-14|MT08563|Palaemon_longirostris|COI-5P
BNSDE366-14|MT08583|Palaemon_longirostris|COI-5P
100
66
BNSDE357-14|MT08565|Palaemon_longirostris|COI-5P
74
BNSDE356-14|MT08564|Palaemon_longirostris|COI-5P
41
BNSDE371-14|MT08992|Palaemon_longirostris|COI-5P
59
BNSC362-11|MT03903|Evadne_spinifera|COI-5P
66
BNSC367-11|MT03908|Evadne_spinifera|COI-5P
98
BNSC369-11|MT03910|Evadne_spinifera|COI-5P
BNSC366-11|MT03907|Evadne_spinifera|COI-5P
100
BNSC363-11|MT03904|Evadne_spinifera|COI-5P
34
BNSC370-11|MT03911|Evadne_spinifera|COI-5P
85
4
89
BNSC364-11|MT03905|Evadne_spinifera|COI-5P
67
BNSC368-11|MT03909|Evadne_spinifera|COI-5P
97
BNSCP042-11|MT00684|Evadne_nordmanni|COI-5P
BNSCP046-11|MT01361|Podon_intermedius|COI-5P
BNSCP047-11|MT01362|Podon_intermedius|COI-5P

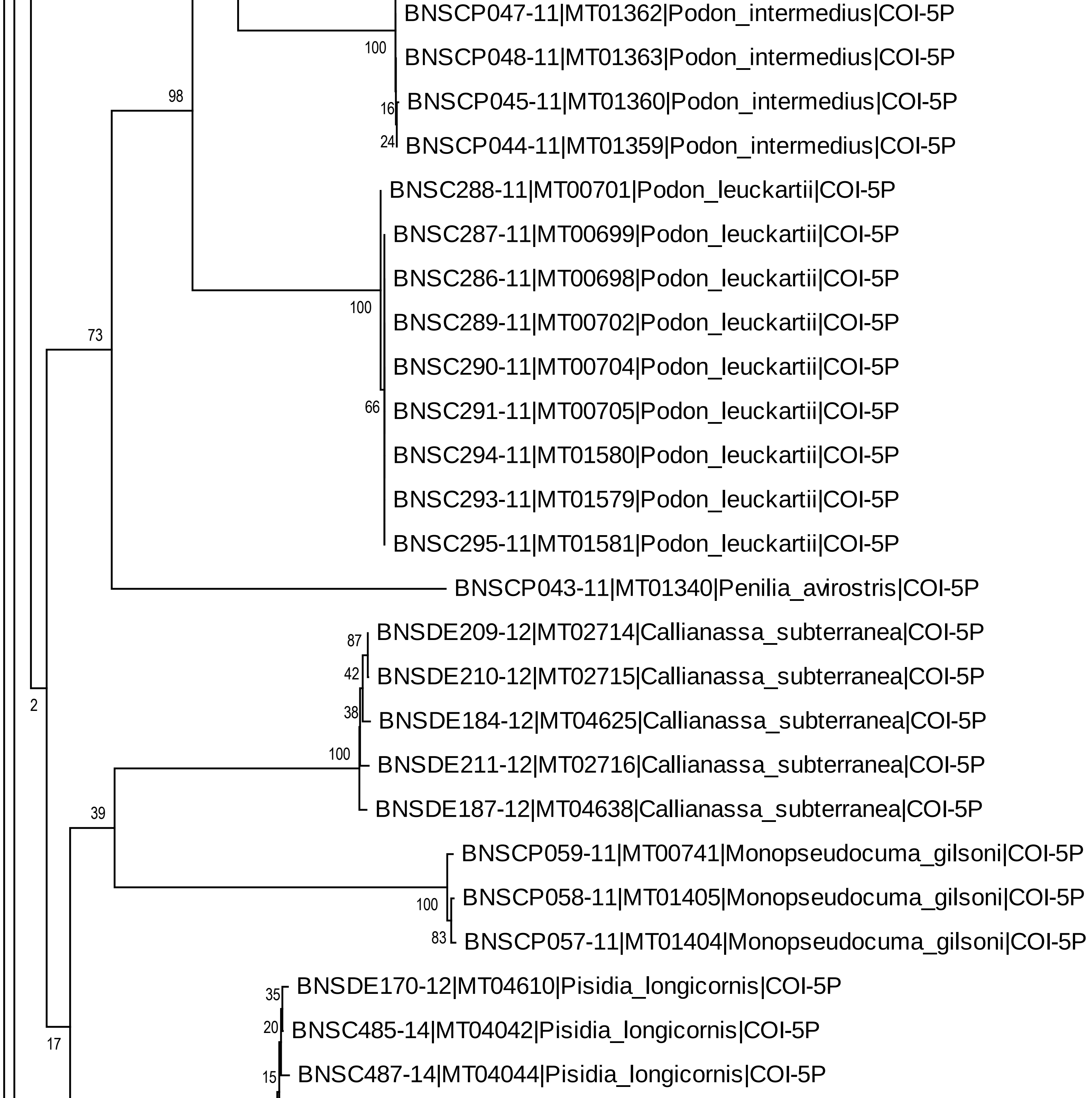
BNSCP047-11|MT01362|Podon_intermedius|COI-5P
100
BNSCP048-11|MT01363|Podon_intermedius|COI-5P
98
16
BNSCP045-11|MT01360|Podon_intermedius|COI-5P
24
BNSCP044-11|MT01359|Podon_intermedius|COI-5P
BNSC288-11|MT00701|Podon_leuckartii|COI-5P
BNSC287-11|MT00699|Podon_leuckartii|COI-5P
BNSC286-11|MT00698|Podon_leuckartii|COI-5P
100
BNSC289-11|MT00702|Podon_leuckartii|COI-5P
73
BNSC290-11|MT00704|Podon_leuckartii|COI-5P
66
BNSC291-11|MT00705|Podon_leuckartii|COI-5P
BNSC294-11|MT01580|Podon_leuckartii|COI-5P
BNSC293-11|MT01579|Podon_leuckartii|COI-5P
BNSC295-11|MT01581|Podon_leuckartii|COI-5P
BNSCP043-11|MT01340|Penilia_avirostris|COI-5P
87
BNSDE209-12|MT02714|Callianassa_subterranea|COI-5P
42
BNSDE210-12|MT02715|Callianassa_subterranea|COI-5P
2
38
BNSDE184-12|MT04625|Callianassa_subterranea|COI-5P
100
BNSDE211-12|MT02716|Callianassa_subterranea|COI-5P
39
BNSDE187-12|MT04638|Callianassa_subterranea|COI-5P
BNSCP059-11|MT00741|Monopseudocuma_gilsoni|COI-5P
100
BNSCP058-11|MT01405|Monopseudocuma_gilsoni|COI-5P
83
BNSCP057-11|MT01404|Monopseudocuma_gilsoni|COI-5P
35
BNSDE170-12|MT04610|Pisidia_longicornis|COI-5P
20
BNSC485-14|MT04042|Pisidia_longicornis|COI-5P
17
15
BNSC487-14|MT04044|Pisidia_longicornis|COI-5P

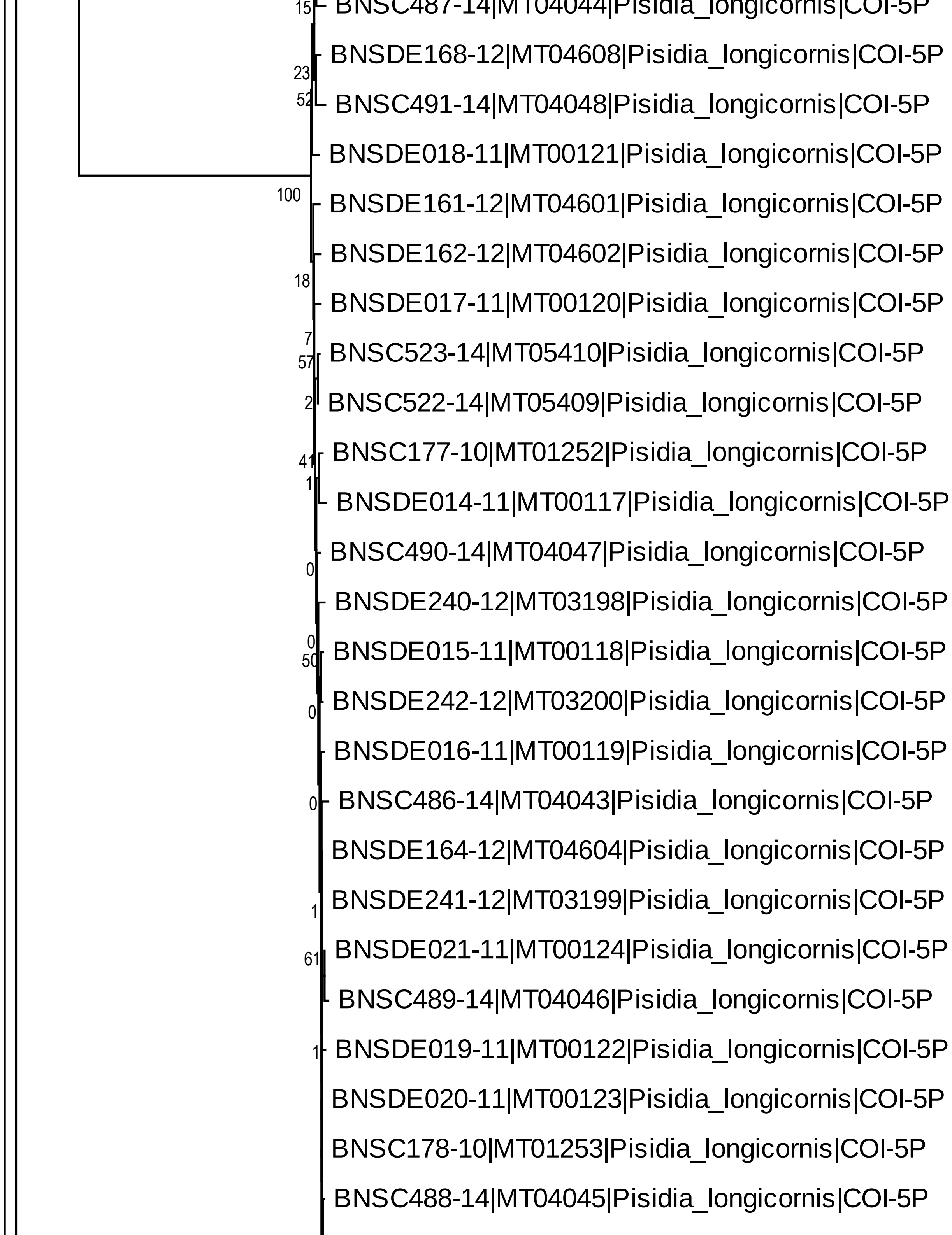
BNSC487-14|MT04044|Pisidia_longicornis|COI-5P
BNSDE168-12|MT04608|Pisidia_longicornis|COI-5P
BNSC491-14|MT04048|Pisidia_longicornis|COI-5P
BNSDE018-11|MT00121|Pisidia_longicornis|COI-5P
BNSDE161-12|MT04601|Pisidia_longicornis|COI-5P
BNSDE162-12|MT04602|Pisidia_longicornis|COI-5P
BNSDE017-11|MT00120|Pisidia_longicornis|COI-5P
BNSC523-14|MT05410|Pisidia_longicornis|COI-5P
BNSC522-14|MT05409|Pisidia_longicornis|COI-5P
BNSC177-10|MT01252|Pisidia_longicornis|COI-5P
BNSDE014-11|MT00117|Pisidia_longicornis|COI-5P
BNSC490-14|MT04047|Pisidia_longicornis|COI-5P
BNSDE240-12|MT03198|Pisidia_longicornis|COI-5P
BNSDE015-11|MT00118|Pisidia_longicornis|COI-5P
BNSDE242-12|MT03200|Pisidia_longicornis|COI-5P
BNSDE016-11|MT00119|Pisidia_longicornis|COI-5P
BNSC486-14|MT04043|Pisidia_longicornis|COI-5P
BNSDE164-12|MT04604|Pisidia_longicornis|COI-5P
BNSDE241-12|MT03199|Pisidia_longicornis|COI-5P
BNSDE021-11|MT00124|Pisidia_longicornis|COI-5P
BNSC489-14|MT04046|Pisidia_longicornis|COI-5P
BNSDE019-11|MT00122|Pisidia_longicornis|COI-5P
BNSDE020-11|MT00123|Pisidia_longicornis|COI-5P
BNSC178-10|MT01253|Pisidia_longicornis|COI-5P
BNSC488-14|MT04045|Pisidia_longicornis|COI-5P

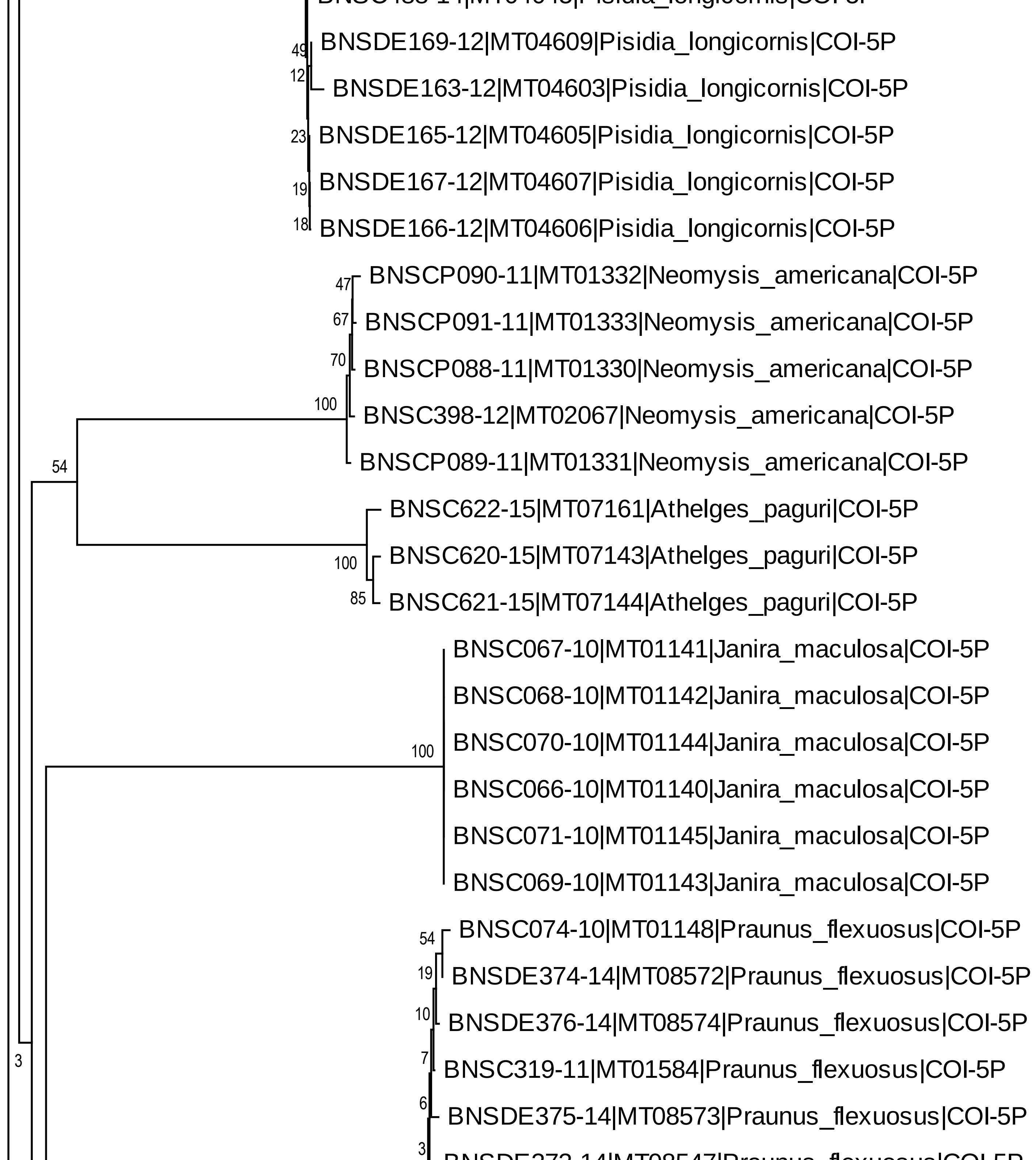
BNSDE169-12|MT04609|Pisidia_longicornis|COI-5P
BNSDE163-12|MT04603|Pisidia_longicornis|COI-5P
BNSDE165-12|MT04605|Pisidia_longicornis|COI-5P
BNSDE167-12|MT04607|Pisidia_longicornis|COI-5P
BNSDE166-12|MT04606|Pisidia_longicornis|COI-5P
BNSCP090-11|MT01332|Neomysis_americana|COI-5P
BNSCP091-11|MT01333|Neomysis_americana|COI-5P
BNSCP088-11|MT01330|Neomysis_americana|COI-5P
BNSC398-12|MT02067|Neomysis_americana|COI-5P
BNSCP089-11|MT01331|Neomysis_americana|COI-5P
BNSC622-15|MT07161|Athelges_paguri|COI-5P
BNSC620-15|MT07143|Athelges_paguri|COI-5P
BNSC621-15|MT07144|Athelges_paguri|COI-5P
BNSC067-10|MT01141|Janira_maculosa|COI-5P
BNSC068-10|MT01142|Janira_maculosa|COI-5P
BNSC070-10|MT01144|Janira_maculosa|COI-5P
BNSC066-10|MT01140|Janira_maculosa|COI-5P
BNSC071-10|MT01145|Janira_maculosa|COI-5P
BNSC069-10|MT01143|Janira_maculosa|COI-5P
BNSC074-10|MT01148|Praunus_flexuosus|COI-5P
BNSDE374-14|MT08572|Praunus_flexuosus|COI-5P
BNSDE376-14|MT08574|Praunus_flexuosus|COI-5P
BNSC319-11|MT01584|Praunus_flexuosus|COI-5P
BNSDE375-14|MT08573|Praunus_flexuosus|COI-5P

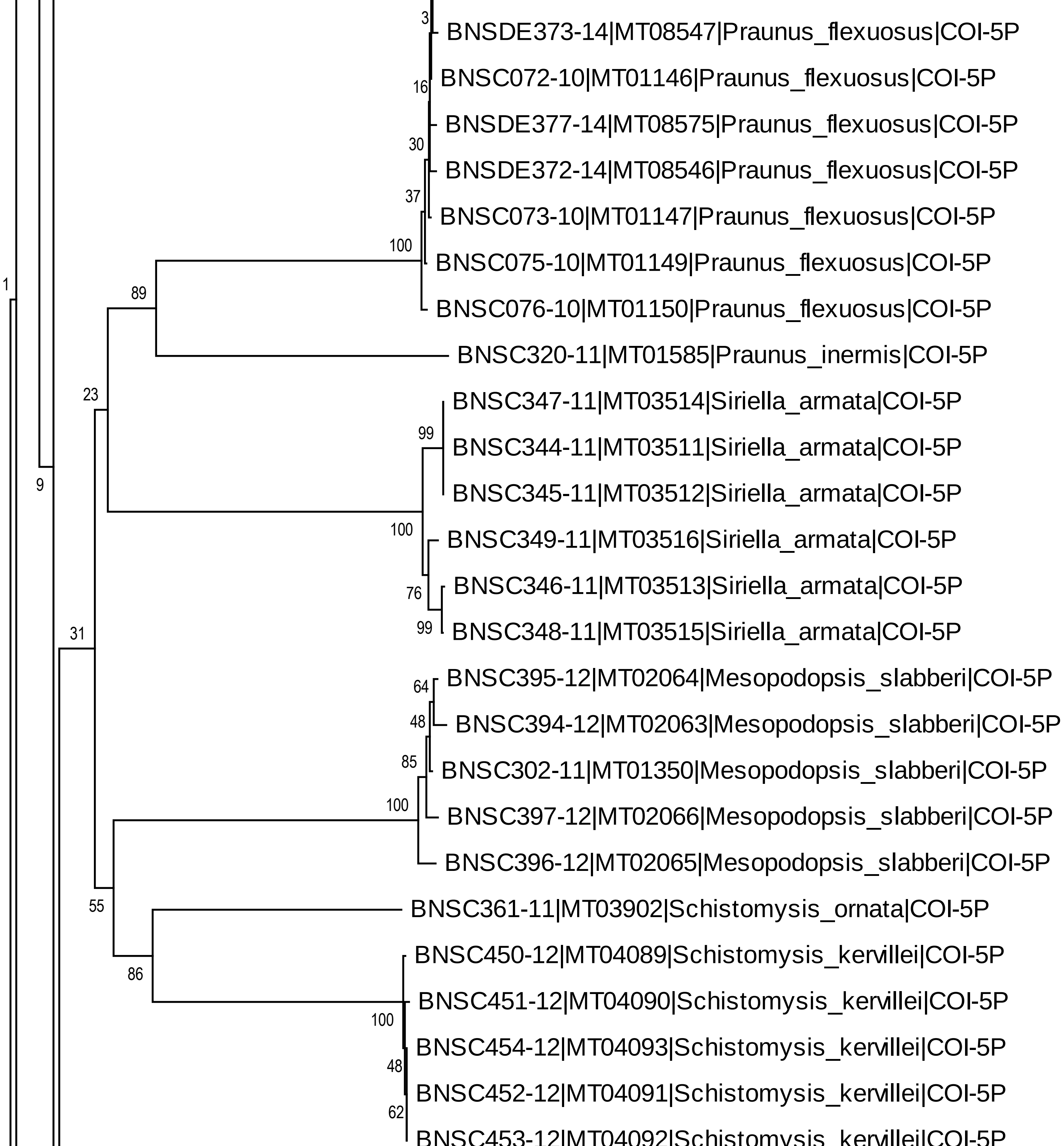

3
BNSDE373-14|MT08547|Praunus_flexuosus|COI-5P
BNSC072-10|MT01146|Praunus_flexuosus|COI-5P
16
BNSDE377-14|MT08575|Praunus_flexuosus|COI-5P
30
BNSDE372-14|MT08546|Praunus_flexuosus|COI-5P
37
BNSC073-10|MT01147|Praunus_flexuosus|COI-5P
100
BNSC075-10|MT01149|Praunus_flexuosus|COI-5P
1
89
BNSC076-10|MT01150|Praunus_flexuosus|COI-5P
BNSC320-11|MT01585|Praunus_inermis|COI-5P
23
BNSC347-11|MT03514|Siriella_armata|COI-5P
99
BNSC344-11|MT03511|Siriella_armata|COI-5P
9
BNSC345-11|MT03512|Siriella_armata|COI-5P
100
BNSC349-11|MT03516|Siriella_armata|COI-5P
BNSC346-11|MT03513|Siriella_armata|COI-5P
76
99
BNSC348-11|MT03515|Siriella_armata|COI-5P
31
BNSC395-12|MT02064|Mesopodopsis_slabberi|COI-5P
64
BNSC394-12|MT02063|Mesopodopsis_slabberi|COI-5P
48
85
BNSC302-11|MT01350|Mesopodopsis_slabberi|COI-5P
100
BNSC397-12|MT02066|Mesopodopsis_slabberi|COI-5P
BNSC396-12|MT02065|Mesopodopsis_slabberi|COI-5P
BNSC361-11|MT03902|Schistomysis_ornata|COI-5P
55
BNSC450-12|MT04089|Schistomysis_kervillei|COI-5P
86
BNSC451-12|MT04090|Schistomysis_kervillei|COI-5P
100
BNSC454-12|MT04093|Schistomysis_kervillei|COI-5P
48
BNSC452-12|MT04091|Schistomysis_kervillei|COI-5P
62
BNSC453-12|MT04092|Schistomysis_kervillei|COI-5P

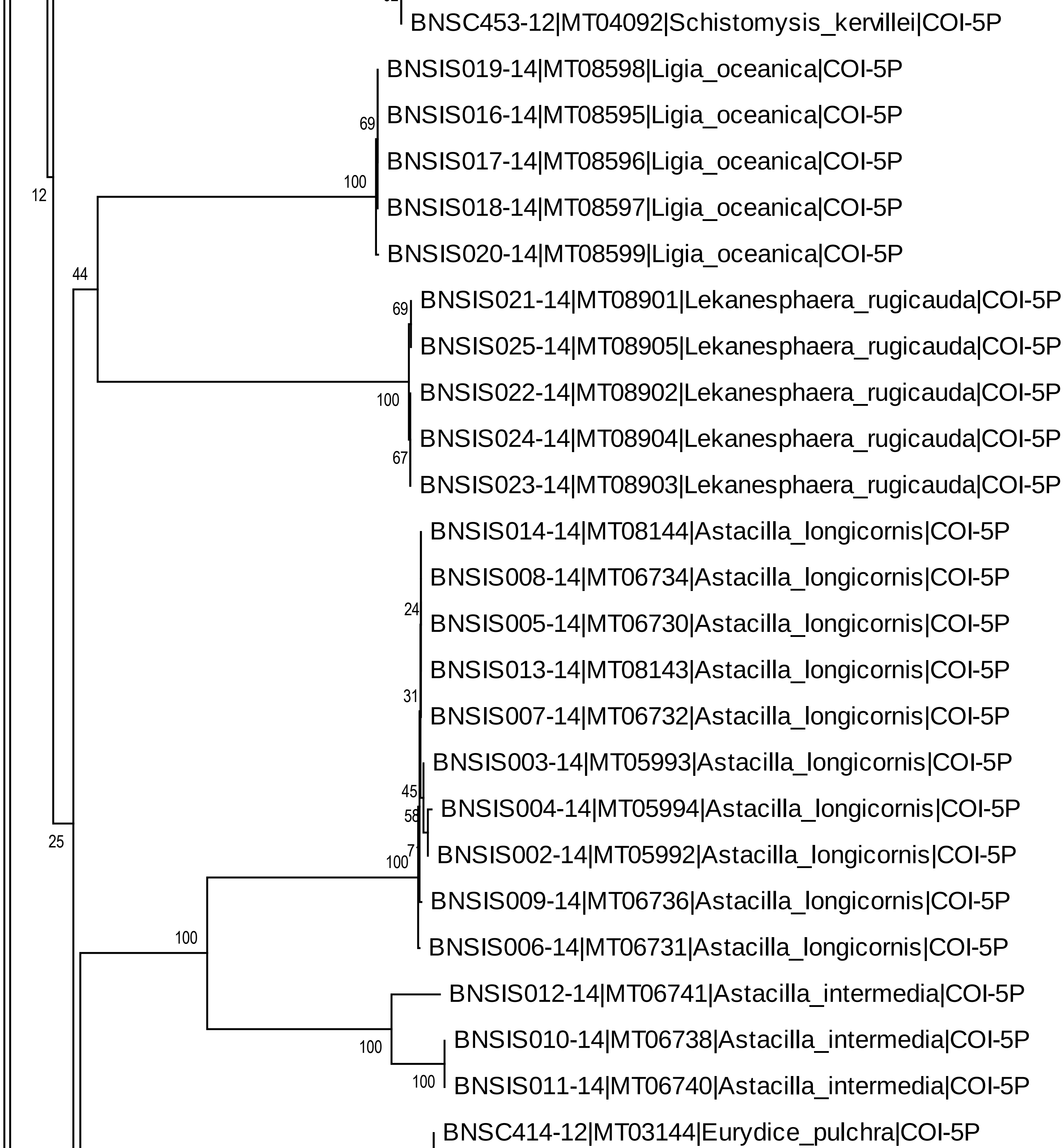
BNSC453-12|MT04092|Schistomysis_kervillei|COI-5P
BNSIS019-14|MT08598|Ligia_oceanica|COI-5P
BNSIS016-14|MT08595|Ligia_oceanica|COI-5P
BNSIS017-14|MT08596|Ligia_oceanica|COI-5P
BNSIS018-14|MT08597|Ligia_oceanica|COI-5P
BNSIS020-14|MT08599|Ligia_oceanica|COI-5P
BNSIS021-14|MT08901|Lekanesphaera_rugicauda|COI-5P
BNSIS025-14|MT08905|Lekanesphaera_rugicauda|COI-5P
BNSIS022-14|MT08902|Lekanesphaera_rugicauda|COI-5P
BNSIS024-14|MT08904|Lekanesphaera_rugicauda|COI-5P
BNSIS023-14|MT08903|Lekanesphaera_rugicauda|COI-5P
BNSIS014-14|MT08144|Astacilla_longicornis|COI-5P
BNSIS008-14|MT06734|Astacilla_longicornis|COI-5P
BNSIS005-14|MT06730|Astacilla_longicornis|COI-5P
BNSIS013-14|MT08143|Astacilla_longicornis|COI-5P
BNSIS007-14|MT06732|Astacilla_longicornis|COI-5P
BNSIS003-14|MT05993|Astacilla_longicornis|COI-5P
BNSIS004-14|MT05994|Astacilla_longicornis|COI-5P
BNSIS002-14|MT05992|Astacilla_longicornis|COI-5P
BNSIS009-14|MT06736|Astacilla_longicornis|COI-5P
BNSIS006-14|MT06731|Astacilla_longicornis|COI-5P
BNSIS012-14|MT06741|Astacilla_intermedia|COI-5P
BNSIS010-14|MT06738|Astacilla_intermedia|COI-5P
BNSIS011-14|MT06740|Astacilla_intermedia|COI-5P
BNSC414-12|MT03144|Eurydice_pulchra|COI-5P
12
44
25
69
100
69
100
67
24
31
45
58
100
100
100
100
100

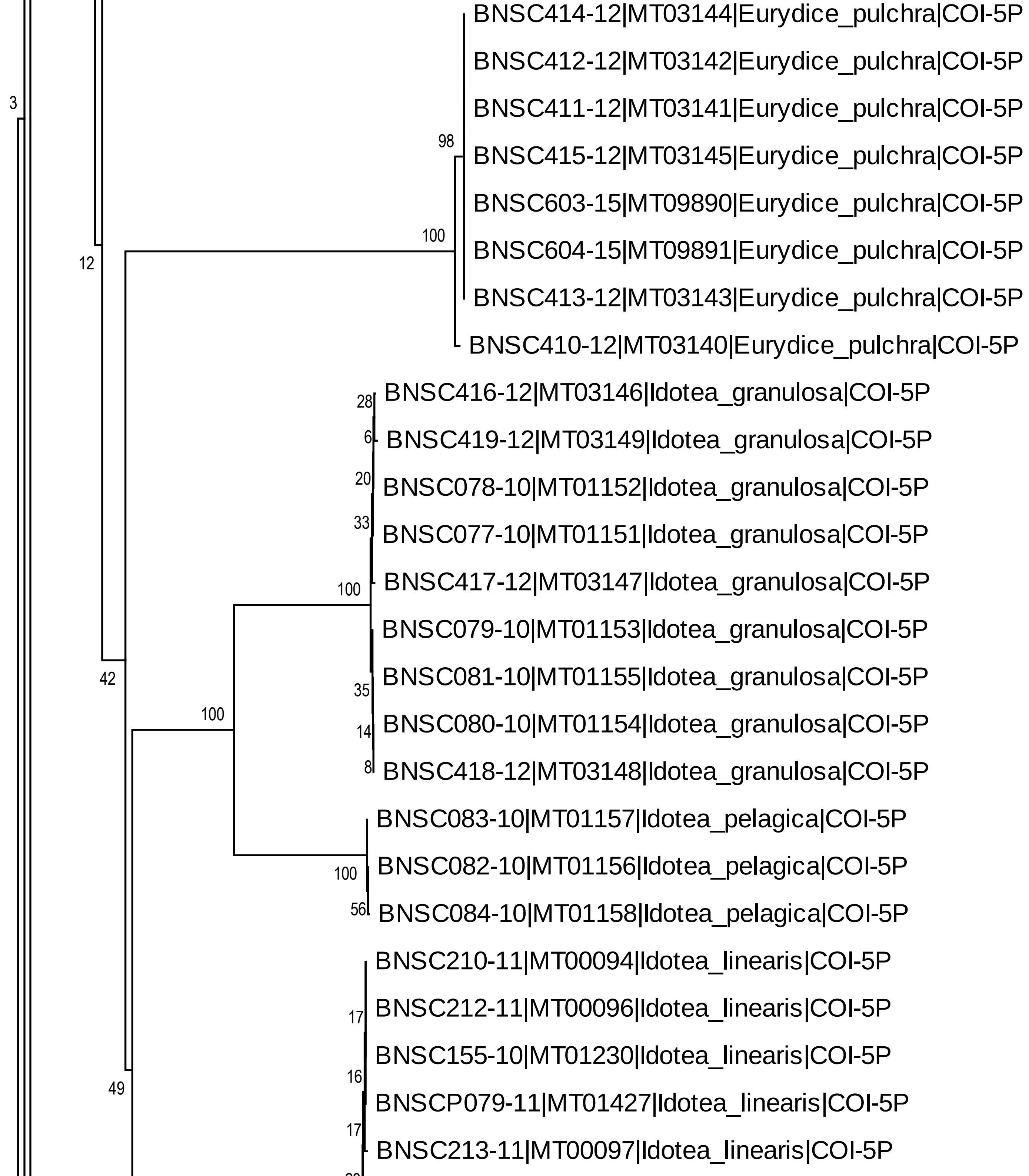

3
12
42
49
BNSC414-12|MT03144|Eurydice_pulchra|COI-5P
BNSC412-12|MT03142|Eurydice_pulchra|COI-5P
BNSC411-12|MT03141|Eurydice_pulchra|COI-5P
98
BNSC415-12|MT03145|Eurydice_pulchra|COI-5P
BNSC603-15|MT09890|Eurydice_pulchra|COI-5P
100
BNSC604-15|MT09891|Eurydice_pulchra|COI-5P
BNSC413-12|MT03143|Eurydice_pulchra|COI-5P
BNSC410-12|MT03140|Eurydice_pulchra|COI-5P
28
BNSC416-12|MT03146|Idotea_granulosa|COI-5P
6
BNSC419-12|MT03149|Idotea_granulosa|COI-5P
20
BNSC078-10|MT01152|Idotea_granulosa|COI-5P
33
BNSC077-10|MT01151|Idotea_granulosa|COI-5P
100
BNSC417-12|MT03147|Idotea_granulosa|COI-5P
BNSC079-10|MT01153|Idotea_granulosa|COI-5P
BNSC081-10|MT01155|Idotea_granulosa|COI-5P
35
14
BNSC080-10|MT01154|Idotea_granulosa|COI-5P
8
BNSC418-12|MT03148|Idotea_granulosa|COI-5P
100
BNSC083-10|MT01157|Idotea_pelagica|COI-5P
BNSC082-10|MT01156|Idotea_pelagica|COI-5P
100
56
BNSC084-10|MT01158|Idotea_pelagica|COI-5P
BNSC210-11|MT00094|Idotea_linearis|COI-5P
17
BNSC212-11|MT00096|Idotea_linearis|COI-5P
BNSC155-10|MT01230|Idotea_linearis|COI-5P
16
BNSCP079-11|MT01427|Idotea_linearis|COI-5P
17
BNSC213-11|MT00097|Idotea_linearis|COI-5P

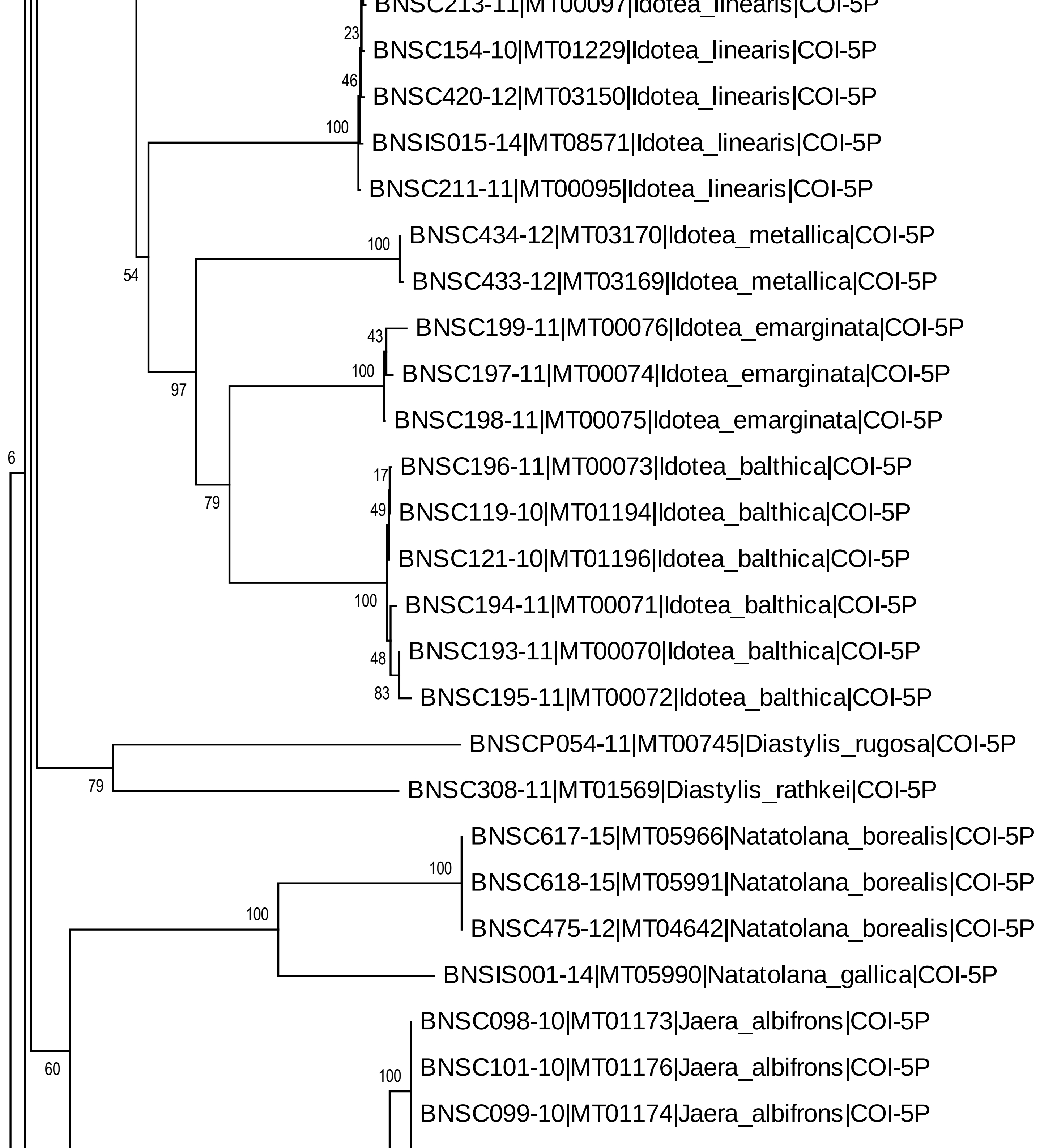
BNSC213-11|MT00097|Idotea_linearis|COI-5P
BNSC154-10|MT01229|Idotea_linearis|COI-5P
BNSC420-12|MT03150|Idotea_linearis|COI-5P
BNSIS015-14|MT08571|Idotea_linearis|COI-5P
BNSC211-11|MT00095|Idotea_linearis|COI-5P
BNSC434-12|MT03170|Idotea_metallica|COI-5P
BNSC433-12|MT03169|Idotea_metallica|COI-5P
BNSC199-11|MT00076|Idotea_emarginata|COI-5P
BNSC197-11|MT00074|Idotea_emarginata|COI-5P
BNSC198-11|MT00075|Idotea_emarginata|COI-5P
BNSC196-11|MT00073|Idotea_balthica|COI-5P
BNSC119-10|MT01194|Idotea_balthica|COI-5P
BNSC121-10|MT01196|Idotea_balthica|COI-5P
BNSC194-11|MT00071|Idotea_balthica|COI-5P
BNSC193-11|MT00070|Idotea_balthica|COI-5P
BNSC195-11|MT00072|Idotea_balthica|COI-5P
BNSCP054-11|MT00745|Diastylis_rugosa|COI-5P
BNSC308-11|MT01569|Diastylis_rathkei|COI-5P
BNSC617-15|MT05966|Natatolana_borealis|COI-5P
BNSC618-15|MT05991|Natatolana_borealis|COI-5P
BNSC475-12|MT04642|Natatolana_borealis|COI-5P
BNSIS001-14|MT05990|Natatolana_gallica|COI-5P
BNSC098-10|MT01173|Jaera_albifrons|COI-5P
BNSC101-10|MT01176|Jaera_albifrons|COI-5P
BNSC099-10|MT01174|Jaera_albifrons|COI-5P

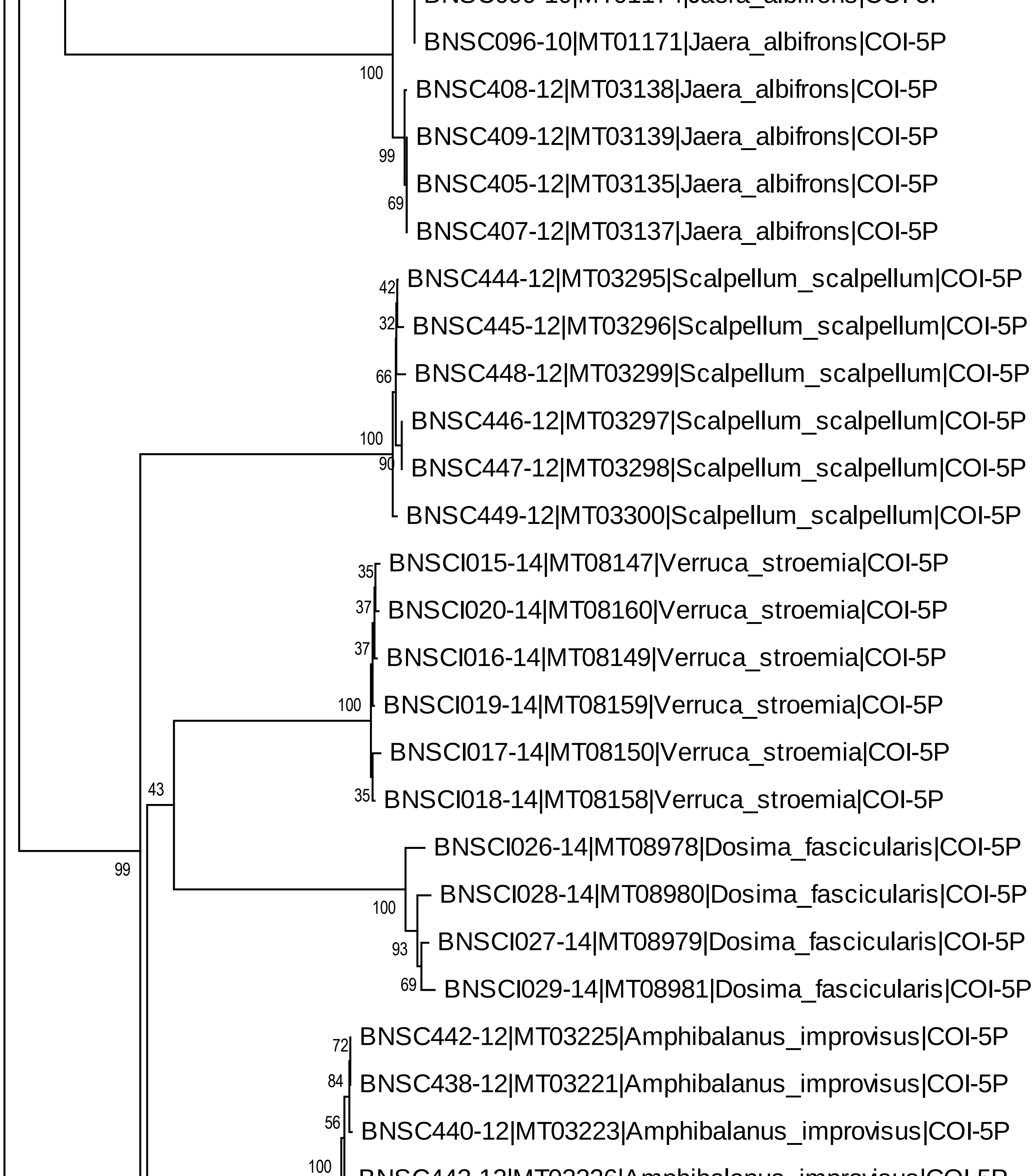
BNSC096-10|MT01171|Jaera_albifrons|COI-5P
100
BNSC408-12|MT03138|Jaera_albifrons|COI-5P
BNSC409-12|MT03139|Jaera_albifrons|COI-5P
99
BNSC405-12|MT03135|Jaera_albifrons|COI-5P
69
BNSC407-12|MT03137|Jaera_albifrons|COI-5P
42
BNSC444-12|MT03295|Scalpellum_scalpellum|COI-5P
32
BNSC445-12|MT03296|Scalpellum_scalpellum|COI-5P
66
BNSC448-12|MT03299|Scalpellum_scalpellum|COI-5P
BNSC446-12|MT03297|Scalpellum_scalpellum|COI-5P
100
90
BNSC447-12|MT03298|Scalpellum_scalpellum|COI-5P
BNSC449-12|MT03300|Scalpellum_scalpellum|COI-5P
35
BNSCI015-14|MT08147|Verruca_stroemia|COI-5P
37
BNSCI020-14|MT08160|Verruca_stroemia|COI-5P
37
BNSCI016-14|MT08149|Verruca_stroemia|COI-5P
100
BNSCI019-14|MT08159|Verruca_stroemia|COI-5P
BNSCI017-14|MT08150|Verruca_stroemia|COI-5P
43
35
BNSCI018-14|MT08158|Verruca_stroemia|COI-5P
BNSCI026-14|MT08978|Dosima_fascicularis|COI-5P
99
100
BNSCI028-14|MT08980|Dosima_fascicularis|COI-5P
93
BNSCI027-14|MT08979|Dosima_fascicularis|COI-5P
69
BNSCI029-14|MT08981|Dosima_fascicularis|COI-5P
72
BNSC442-12|MT03225|Amphibalanus_improvisus|COI-5P
84
BNSC438-12|MT03221|Amphibalanus_improvisus|COI-5P
56
BNSC440-12|MT03223|Amphibalanus_improvisus|COI-5P
100

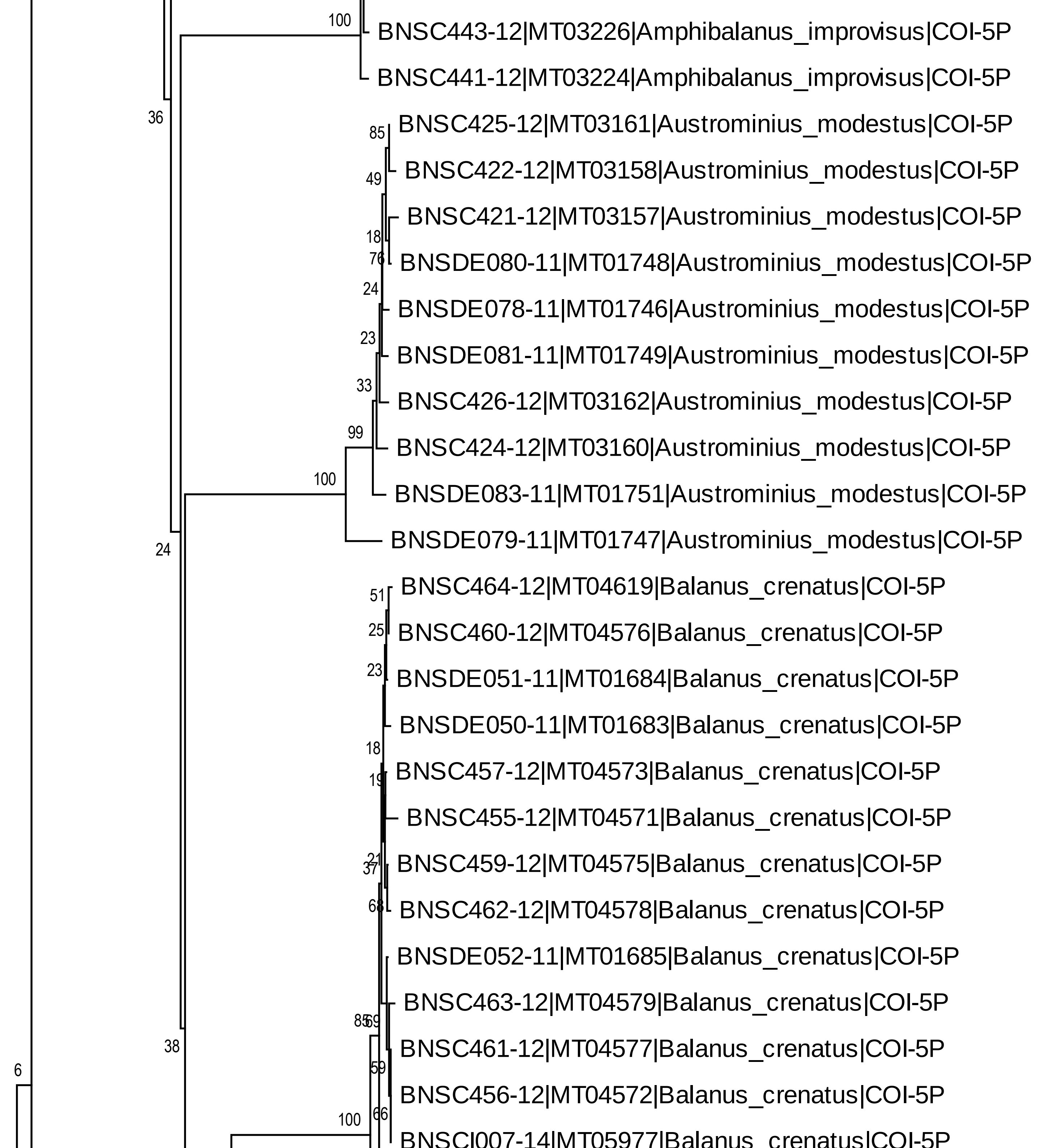

100
BNSC443-12|MT03226|Amphibalanus_improvisus|COI-5P
BNSC441-12|MT03224|Amphibalanus_improvisus|COI-5P
36
85
BNSC425-12|MT03161|Austrominius_modestus|COI-5P
49
BNSC422-12|MT03158|Austrominius_modestus|COI-5P
BNSC421-12|MT03157|Austrominius_modestus|COI-5P
18
76
BNSDE080-11|MT01748|Austrominius_modestus|COI-5P
24
BNSDE078-11|MT01746|Austrominius_modestus|COI-5P
23
BNSDE081-11|MT01749|Austrominius_modestus|COI-5P
33
BNSC426-12|MT03162|Austrominius_modestus|COI-5P
99
BNSC424-12|MT03160|Austrominius_modestus|COI-5P
100
BNSDE083-11|MT01751|Austrominius_modestus|COI-5P
BNSDE079-11|MT01747|Austrominius_modestus|COI-5P
24
BNSC464-12|MT04619|Balanus_crenatus|COI-5P
51
25
BNSC460-12|MT04576|Balanus_crenatus|COI-5P
23
BNSDE051-11|MT01684|Balanus_crenatus|COI-5P
BNSDE050-11|MT01683|Balanus_crenatus|COI-5P
18
BNSC457-12|MT04573|Balanus_crenatus|COI-5P
19
BNSC455-12|MT04571|Balanus_crenatus|COI-5P
21
37
BNSC459-12|MT04575|Balanus_crenatus|COI-5P
68
BNSC462-12|MT04578|Balanus_crenatus|COI-5P
BNSDE052-11|MT01685|Balanus_crenatus|COI-5P
BNSC463-12|MT04579|Balanus_crenatus|COI-5P
85
69
38
BNSC461-12|MT04577|Balanus_crenatus|COI-5P
6
59
BNSC456-12|MT04572|Balanus_crenatus|COI-5P
100
66

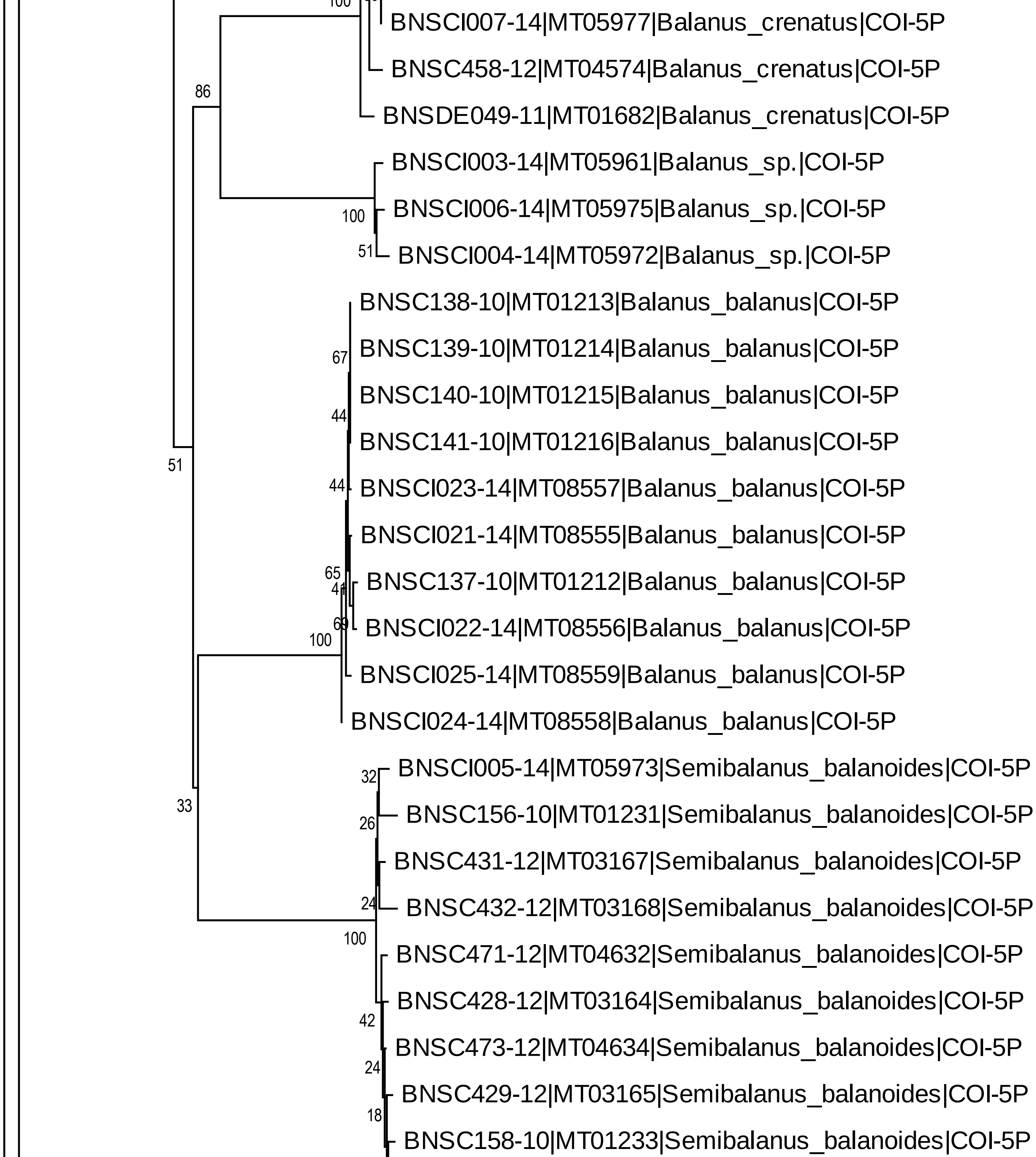
100
BNSCI007-14|MT05977|Balanus_crenatus|COI-5P
BNSC458-12|MT04574|Balanus_crenatus|COI-5P
86
BNSDE049-11|MT01682|Balanus_crenatus|COI-5P
BNSCI003-14|MT05961|Balanus_sp.|COI-5P
100
BNSCI006-14|MT05975|Balanus_sp.|COI-5P
51
BNSCI004-14|MT05972|Balanus_sp.|COI-5P
BNSC138-10|MT01213|Balanus_balanus|COI-5P
67
BNSC139-10|MT01214|Balanus_balanus|COI-5P
BNSC140-10|MT01215|Balanus_balanus|COI-5P
44
BNSC141-10|MT01216|Balanus_balanus|COI-5P
51
44
BNSCI023-14|MT08557|Balanus_balanus|COI-5P
BNSCI021-14|MT08555|Balanus_balanus|COI-5P
65
BNSC137-10|MT01212|Balanus_balanus|COI-5P
69
BNSCI022-14|MT08556|Balanus_balanus|COI-5P
100
BNSCI025-14|MT08559|Balanus_balanus|COI-5P
BNSCI024-14|MT08558|Balanus_balanus|COI-5P
32
BNSCI005-14|MT05973|Semibalanus_balanoides|COI-5P
33
26
BNSC156-10|MT01231|Semibalanus_balanoides|COI-5P
BNSC431-12|MT03167|Semibalanus_balanoides|COI-5P
24
BNSC432-12|MT03168|Semibalanus_balanoides|COI-5P
100
BNSC471-12|MT04632|Semibalanus_balanoides|COI-5P
BNSC428-12|MT03164|Semibalanus_balanoides|COI-5P
42
BNSC473-12|MT04634|Semibalanus_balanoides|COI-5P
24
BNSC429-12|MT03165|Semibalanus_balanoides|COI-5P
18
BNSC158-10|MT01233|Semibalanus_balanoides|COI-5P

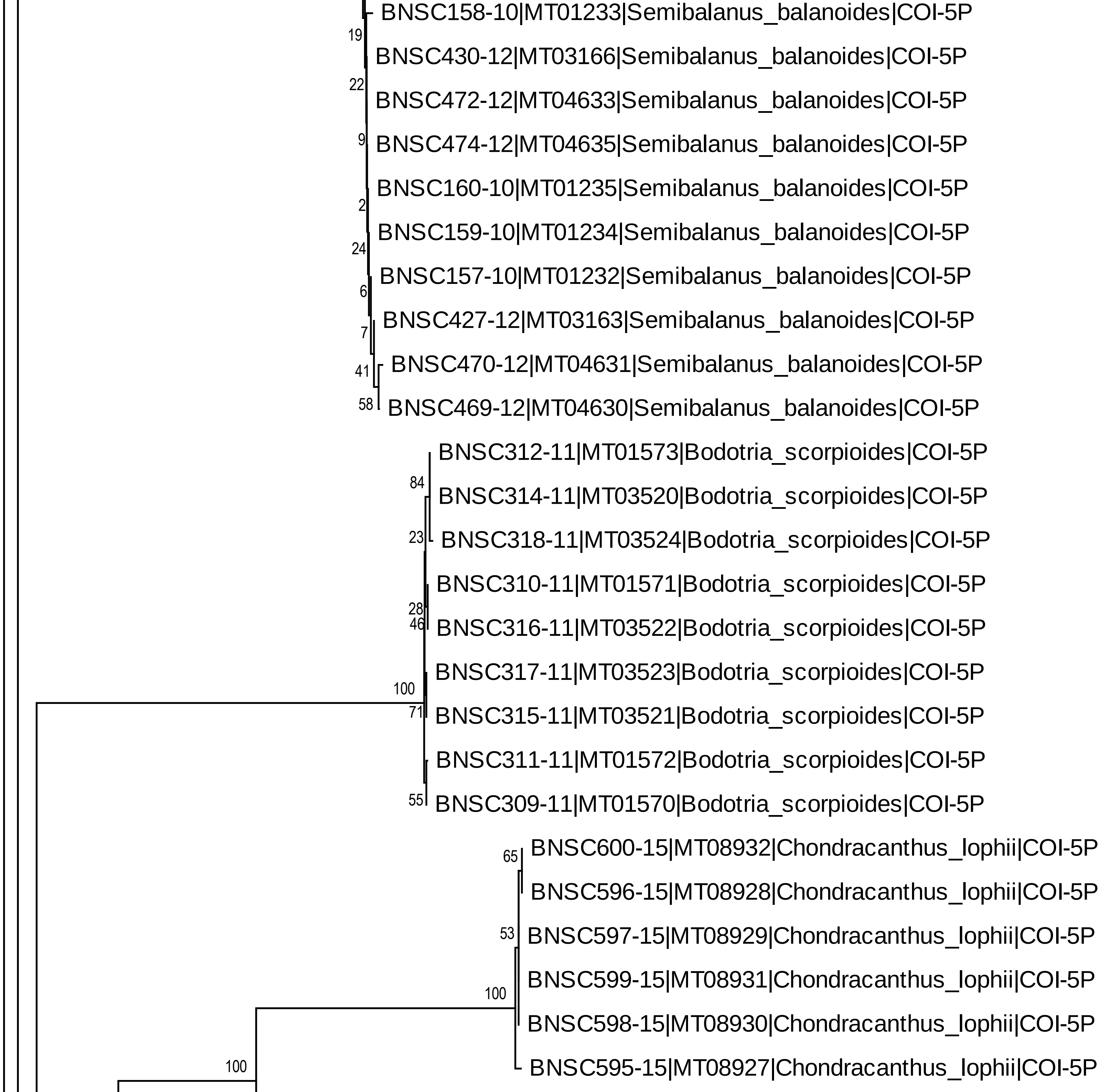

BNSC158-10|MT01233|Semibalanus_balanoides|COI-5P
19
BNSC430-12|MT03166|Semibalanus_balanoides|COI-5P
22
BNSC472-12|MT04633|Semibalanus_balanoides|COI-5P
9
BNSC474-12|MT04635|Semibalanus_balanoides|COI-5P
BNSC160-10|MT01235|Semibalanus_balanoides|COI-5P
2
BNSC159-10|MT01234|Semibalanus_balanoides|COI-5P
24
BNSC157-10|MT01232|Semibalanus_balanoides|COI-5P
6
BNSC427-12|MT03163|Semibalanus_balanoides|COI-5P
7
BNSC470-12|MT04631|Semibalanus_balanoides|COI-5P
41
58
BNSC469-12|MT04630|Semibalanus_balanoides|COI-5P
BNSC312-11|MT01573|Bodotria_scorpioides|COI-5P
84
BNSC314-11|MT03520|Bodotria_scorpioides|COI-5P
23
BNSC318-11|MT03524|Bodotria_scorpioides|COI-5P
BNSC310-11|MT01571|Bodotria_scorpioides|COI-5P
28
46
BNSC316-11|MT03522|Bodotria_scorpioides|COI-5P
BNSC317-11|MT03523|Bodotria_scorpioides|COI-5P
100
71
BNSC315-11|MT03521|Bodotria_scorpioides|COI-5P
BNSC311-11|MT01572|Bodotria_scorpioides|COI-5P
55
BNSC309-11|MT01570|Bodotria_scorpioides|COI-5P
BNSC600-15|MT08932|Chondracanthus_lophii|COI-5P
65
BNSC596-15|MT08928|Chondracanthus_lophii|COI-5P
53
BNSC597-15|MT08929|Chondracanthus_lophii|COI-5P
BNSC599-15|MT08931|Chondracanthus_lophii|COI-5P
100
BNSC598-15|MT08930|Chondracanthus_lophii|COI-5P
100
BNSC595-15|MT08927|Chondracanthus_lophii|COI-5P

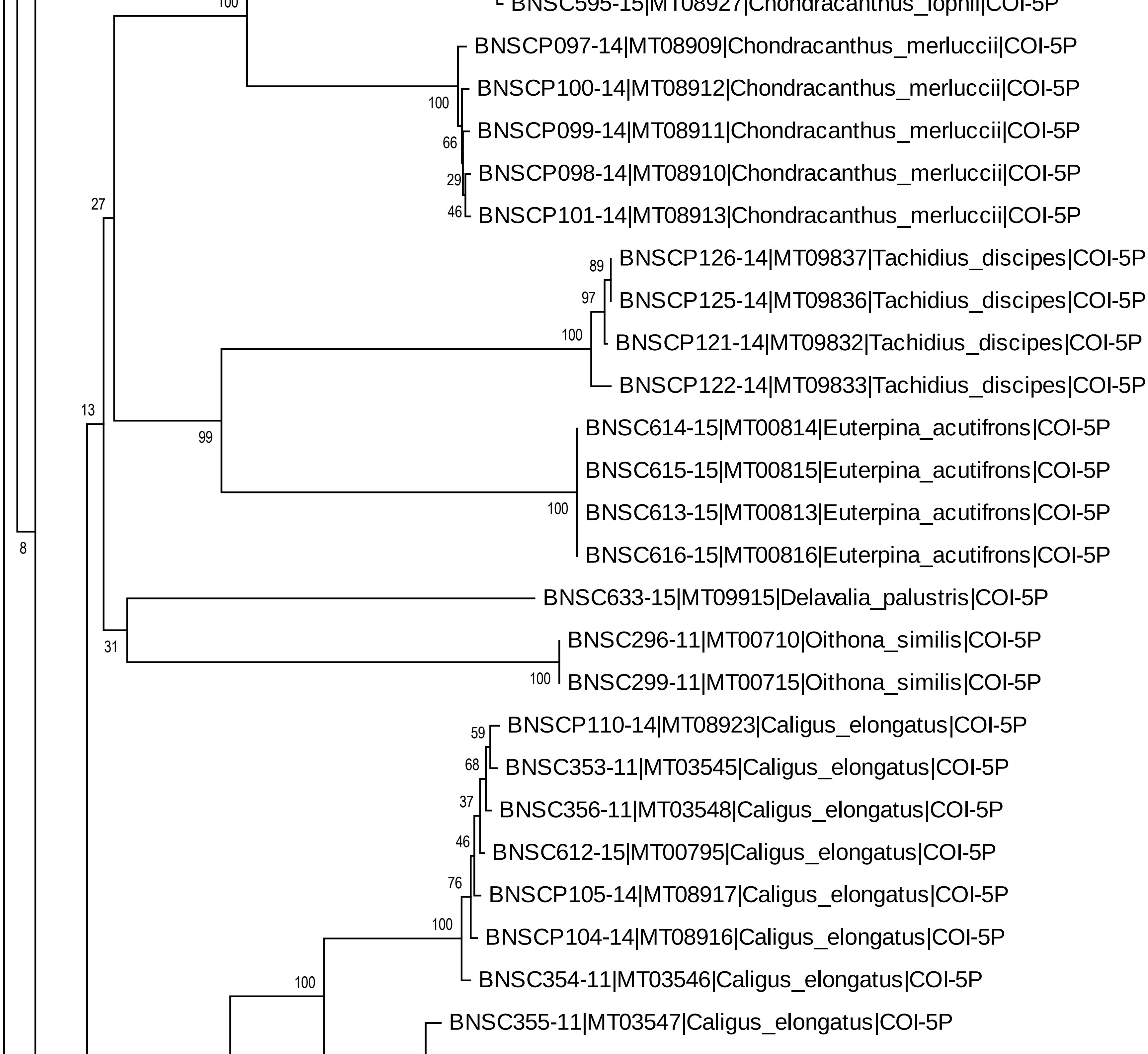

BNSC595-15|MT08927|Chondracanthus_lophii|COI-5P
BNSCP097-14|MT08909|Chondracanthus_merluccii|COI-5P
BNSCP100-14|MT08912|Chondracanthus_merluccii|COI-5P
BNSCP099-14|MT08911|Chondracanthus_merluccii|COI-5P
BNSCP098-14|MT08910|Chondracanthus_merluccii|COI-5P
BNSCP101-14|MT08913|Chondracanthus_merluccii|COI-5P
BNSCP126-14|MT09837|Tachidius_discipes|COI-5P
BNSCP125-14|MT09836|Tachidius_discipes|COI-5P
BNSCP121-14|MT09832|Tachidius_discipes|COI-5P
BNSCP122-14|MT09833|Tachidius_discipes|COI-5P
BNSC614-15|MT00814|Euterpina_acutifrons|COI-5P
BNSC615-15|MT00815|Euterpina_acutifrons|COI-5P
BNSC613-15|MT00813|Euterpina_acutifrons|COI-5P
BNSC616-15|MT00816|Euterpina_acutifrons|COI-5P
BNSC633-15|MT09915|Delavalia_palustris|COI-5P
BNSC296-11|MT00710|Oithona_similis|COI-5P
BNSC299-11|MT00715|Oithona_similis|COI-5P
BNSCP110-14|MT08923|Caligus_elongatus|COI-5P
BNSC353-11|MT03545|Caligus_elongatus|COI-5P
BNSC356-11|MT03548|Caligus_elongatus|COI-5P
BNSC612-15|MT00795|Caligus_elongatus|COI-5P
BNSCP105-14|MT08917|Caligus_elongatus|COI-5P
BNSCP104-14|MT08916|Caligus_elongatus|COI-5P
BNSC354-11|MT03546|Caligus_elongatus|COI-5P
BNSC355-11|MT03547|Caligus_elongatus|COI-5P

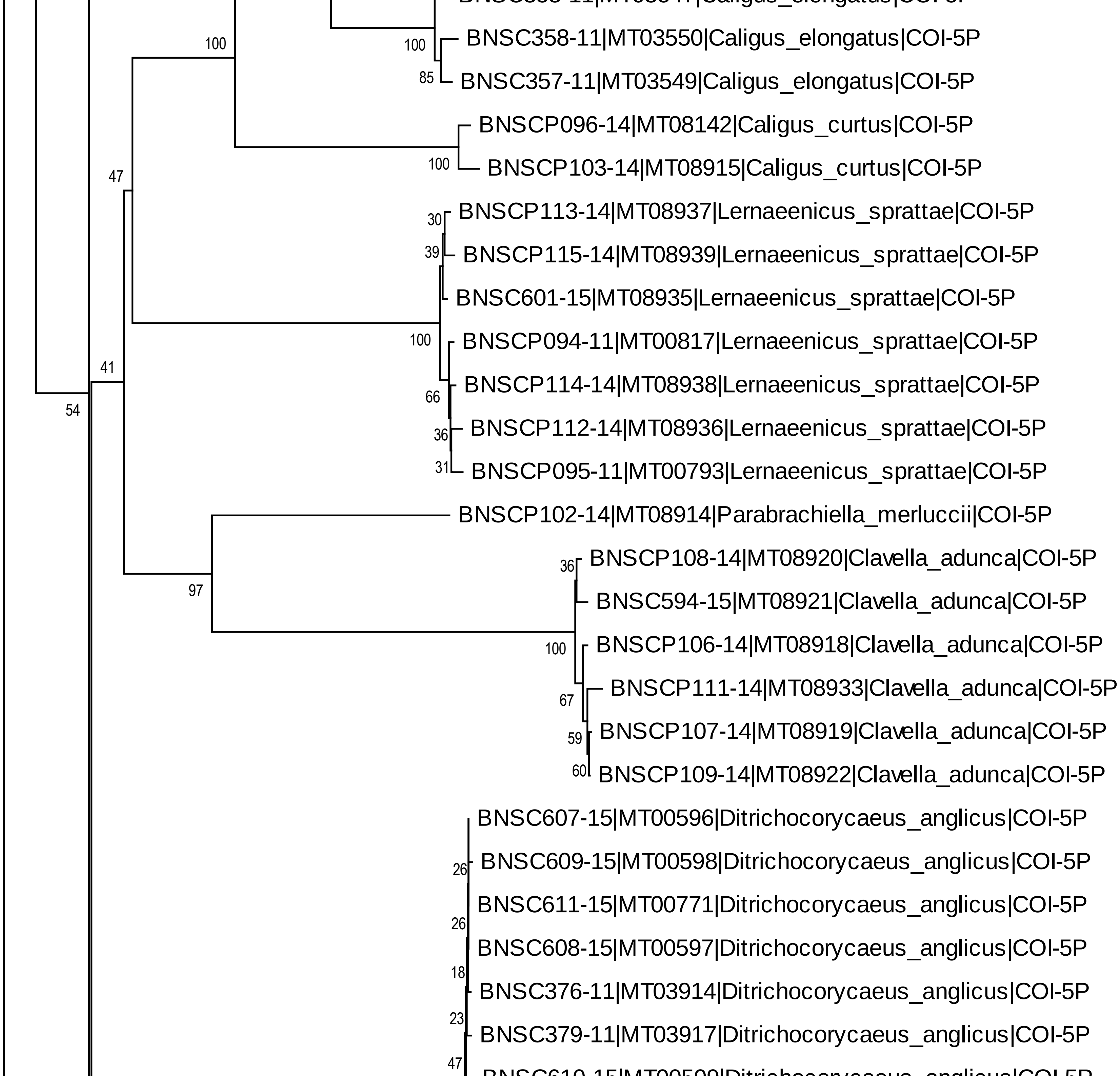
BNSC358-11|MT03550|Caligus_elongatus|COI-5P
BNSC357-11|MT03549|Caligus_elongatus|COI-5P
BNSCP096-14|MT08142|Caligus_curtus|COI-5P
BNSCP103-14|MT08915|Caligus_curtus|COI-5P
BNSCP113-14|MT08937|Lernaeenicus_sprattae|COI-5P
BNSCP115-14|MT08939|Lernaeenicus_sprattae|COI-5P
BNSC601-15|MT08935|Lernaeenicus_sprattae|COI-5P
BNSCP094-11|MT00817|Lernaeenicus_sprattae|COI-5P
BNSCP114-14|MT08938|Lernaeenicus_sprattae|COI-5P
BNSCP112-14|MT08936|Lernaeenicus_sprattae|COI-5P
BNSCP095-11|MT00793|Lernaeenicus_sprattae|COI-5P
BNSCP102-14|MT08914|Parabrachiella_merluccii|COI-5P
BNSCP108-14|MT08920|Clavella_adunca|COI-5P
BNSC594-15|MT08921|Clavella_adunca|COI-5P
BNSCP106-14|MT08918|Clavella_adunca|COI-5P
BNSCP111-14|MT08933|Clavella_adunca|COI-5P
BNSCP107-14|MT08919|Clavella_adunca|COI-5P
BNSCP109-14|MT08922|Clavella_adunca|COI-5P
BNSC607-15|MT00596|Ditrichocorycaeus_anglicus|COI-5P
BNSC609-15|MT00598|Ditrichocorycaeus_anglicus|COI-5P
BNSC611-15|MT00771|Ditrichocorycaeus_anglicus|COI-5P
BNSC608-15|MT00597|Ditrichocorycaeus_anglicus|COI-5P
BNSC376-11|MT03914|Ditrichocorycaeus_anglicus|COI-5P
BNSC379-11|MT03917|Ditrichocorycaeus_anglicus|COI-5P

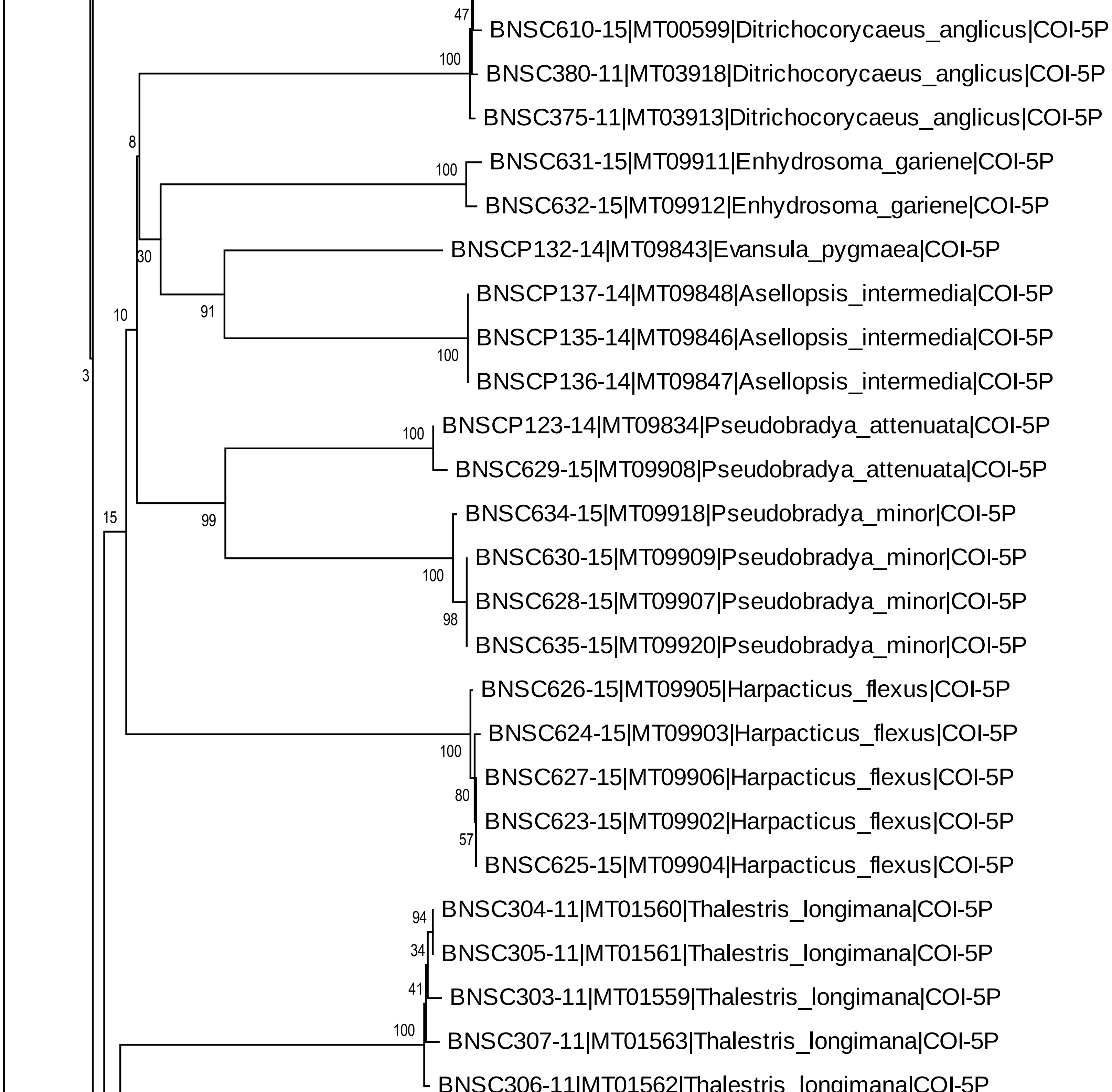

47
BNSC610-15|MT00599|Ditrichocorycaeus_anglicus|COI-5P
100
BNSC380-11|MT03918|Ditrichocorycaeus_anglicus|COI-5P
BNSC375-11|MT03913|Ditrichocorycaeus_anglicus|COI-5P
8
100
BNSC631-15|MT09911|Enhydrosoma_gariene|COI-5P
BNSC632-15|MT09912|Enhydrosoma_gariene|COI-5P
BNSCP132-14|MT09843|Evansula_pygmaea|COI-5P
30
BNSCP137-14|MT09848|Asellopsis_intermedia|COI-5P
10
91
BNSCP135-14|MT09846|Asellopsis_intermedia|COI-5P
100
BNSCP136-14|MT09847|Asellopsis_intermedia|COI-5P
3
100
BNSCP123-14|MT09834|Pseudobradya_attenuata|COI-5P
BNSC629-15|MT09908|Pseudobradya_attenuata|COI-5P
15
99
BNSC634-15|MT09918|Pseudobradya_minor|COI-5P
BNSC630-15|MT09909|Pseudobradya_minor|COI-5P
100
BNSC628-15|MT09907|Pseudobradya_minor|COI-5P
98
BNSC635-15|MT09920|Pseudobradya_minor|COI-5P
BNSC626-15|MT09905|Harpacticus_flexus|COI-5P
BNSC624-15|MT09903|Harpacticus_flexus|COI-5P
100
BNSC627-15|MT09906|Harpacticus_flexus|COI-5P
80
BNSC623-15|MT09902|Harpacticus_flexus|COI-5P
57
BNSC625-15|MT09904|Harpacticus_flexus|COI-5P
94
BNSC304-11|MT01560|Thalestris_longimana|COI-5P
34
BNSC305-11|MT01561|Thalestris_longimana|COI-5P
41
BNSC303-11|MT01559|Thalestris_longimana|COI-5P
100
BNSC307-11|MT01563|Thalestris_longimana|COI-5P
BNSC306-11|MT01562|Thalestris_longimana|COI-5P

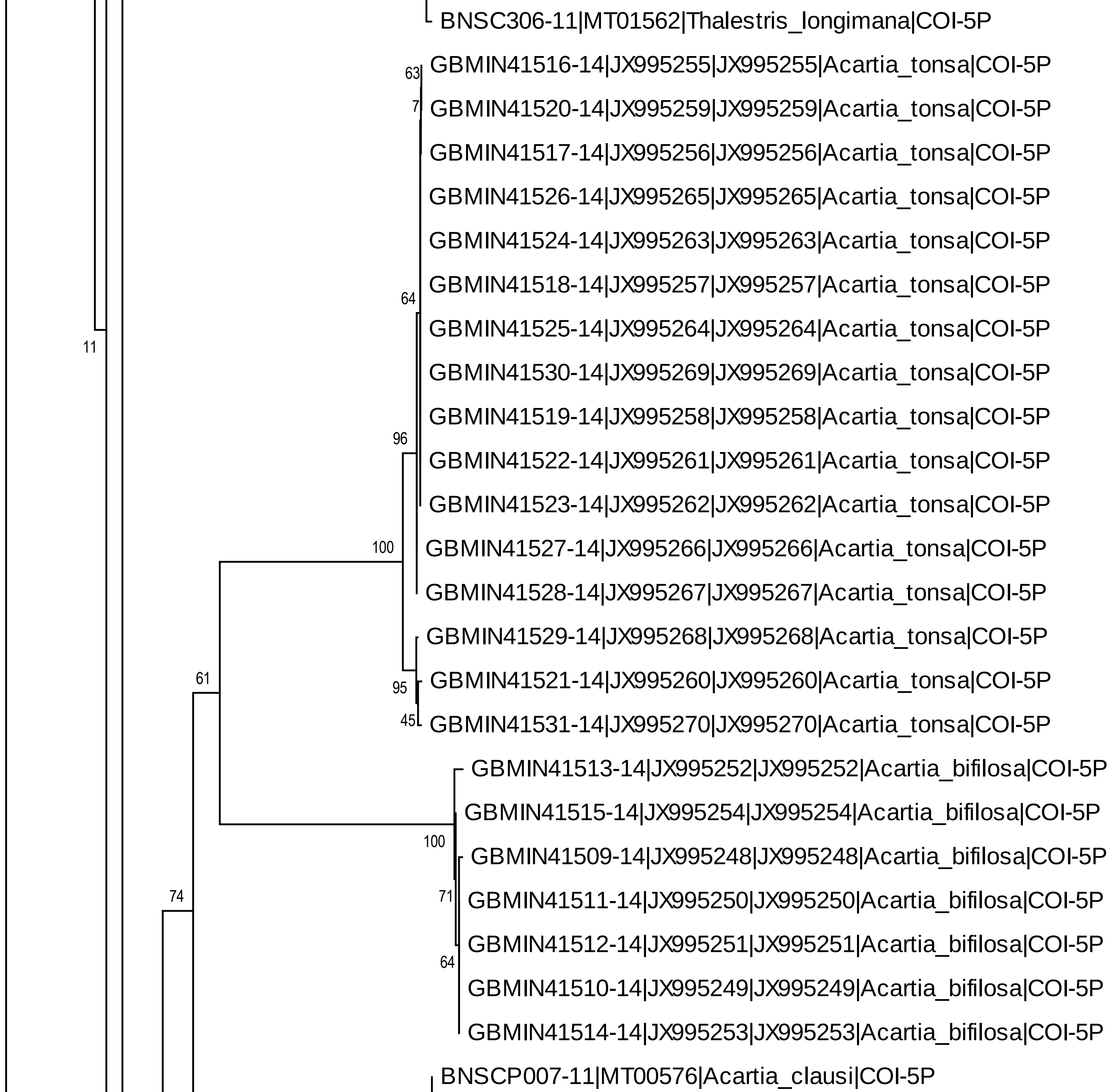
BNSC306-11|MT01562|Thalestris_longimana|COI-5P
63
GBMIN41516-14|JX995255|JX995255|Acartia_tonsa|COI-5P
7
GBMIN41520-14|JX995259|JX995259|Acartia_tonsa|COI-5P
GBMIN41517-14|JX995256|JX995256|Acartia_tonsa|COI-5P
GBMIN41526-14|JX995265|JX995265|Acartia_tonsa|COI-5P
GBMIN41524-14|JX995263|JX995263|Acartia_tonsa|COI-5P
64
GBMIN41518-14|JX995257|JX995257|Acartia_tonsa|COI-5P
11
GBMIN41525-14|JX995264|JX995264|Acartia_tonsa|COI-5P
GBMIN41530-14|JX995269|JX995269|Acartia_tonsa|COI-5P
GBMIN41519-14|JX995258|JX995258|Acartia_tonsa|COI-5P
96
GBMIN41522-14|JX995261|JX995261|Acartia_tonsa|COI-5P
GBMIN41523-14|JX995262|JX995262|Acartia_tonsa|COI-5P
100
GBMIN41527-14|JX995266|JX995266|Acartia_tonsa|COI-5P
GBMIN41528-14|JX995267|JX995267|Acartia_tonsa|COI-5P
GBMIN41529-14|JX995268|JX995268|Acartia_tonsa|COI-5P
61
95
GBMIN41521-14|JX995260|JX995260|Acartia_tonsa|COI-5P
45
GBMIN41531-14|JX995270|JX995270|Acartia_tonsa|COI-5P
GBMIN41513-14|JX995252|JX995252|Acartia_bifilosa|COI-5P
GBMIN41515-14|JX995254|JX995254|Acartia_bifilosa|COI-5P
100
GBMIN41509-14|JX995248|JX995248|Acartia_bifilosa|COI-5P
74
71
GBMIN41511-14|JX995250|JX995250|Acartia_bifilosa|COI-5P
GBMIN41512-14|JX995251|JX995251|Acartia_bifilosa|COI-5P
64
GBMIN41510-14|JX995249|JX995249|Acartia_bifilosa|COI-5P
GBMIN41514-14|JX995253|JX995253|Acartia_bifilosa|COI-5P
BNSCP007-11|MT00576|Acartia_clausi|COI-5P

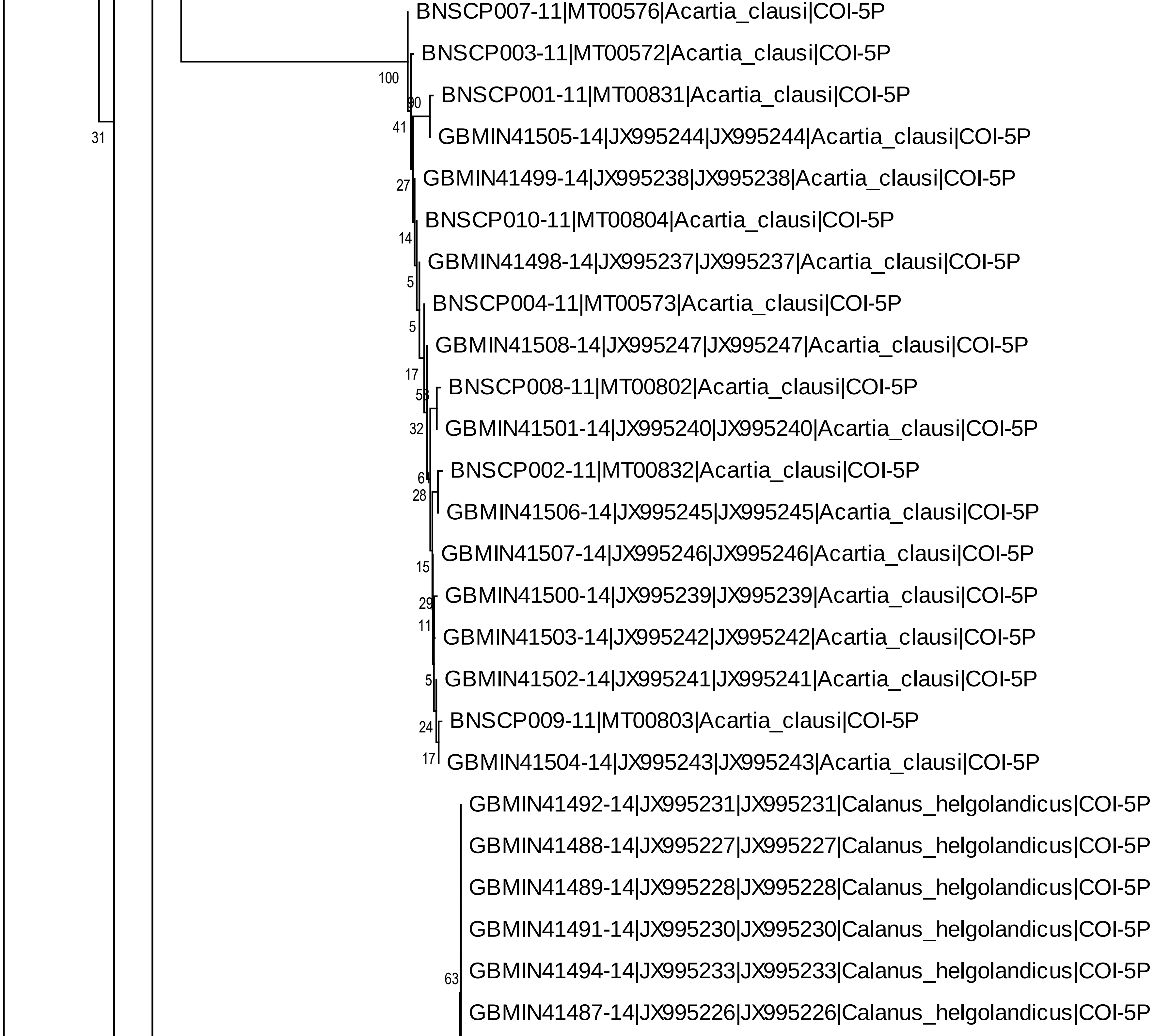
BNSCP007-11|MT00576|Acartia_clausi|COI-5P
BNSCP003-11|MT00572|Acartia_clausi|COI-5P
100
BNSCP001-11|MT00831|Acartia_clausi|COI-5P
90
31
41
GBMIN41505-14|JX995244|JX995244|Acartia_clausi|COI-5P
GBMIN41499-14|JX995238|JX995238|Acartia_clausi|COI-5P
27
BNSCP010-11|MT00804|Acartia_clausi|COI-5P
14
GBMIN41498-14|JX995237|JX995237|Acartia_clausi|COI-5P
5
BNSCP004-11|MT00573|Acartia_clausi|COI-5P
5
GBMIN41508-14|JX995247|JX995247|Acartia_clausi|COI-5P
17
BNSCP008-11|MT00802|Acartia_clausi|COI-5P
58
32
GBMIN41501-14|JX995240|JX995240|Acartia_clausi|COI-5P
BNSCP002-11|MT00832|Acartia_clausi|COI-5P
6
28
GBMIN41506-14|JX995245|JX995245|Acartia_clausi|COI-5P
GBMIN41507-14|JX995246|JX995246|Acartia_clausi|COI-5P
15
GBMIN41500-14|JX995239|JX995239|Acartia_clausi|COI-5P
29
11
GBMIN41503-14|JX995242|JX995242|Acartia_clausi|COI-5P
5
GBMIN41502-14|JX995241|JX995241|Acartia_clausi|COI-5P
BNSCP009-11|MT00803|Acartia_clausi|COI-5P
24
17
GBMIN41504-14|JX995243|JX995243|Acartia_clausi|COI-5P
GBMIN41492-14|JX995231|JX995231|Calanus_helgolandicus|COI-5P
GBMIN41488-14|JX995227|JX995227|Calanus_helgolandicus|COI-5P
GBMIN41489-14|JX995228|JX995228|Calanus_helgolandicus|COI-5P
GBMIN41491-14|JX995230|JX995230|Calanus_helgolandicus|COI-5P
GBMIN41494-14|JX995233|JX995233|Calanus_helgolandicus|COI-5P
63
GBMIN41487-14|JX995226|JX995226|Calanus_helgolandicus|COI-5P

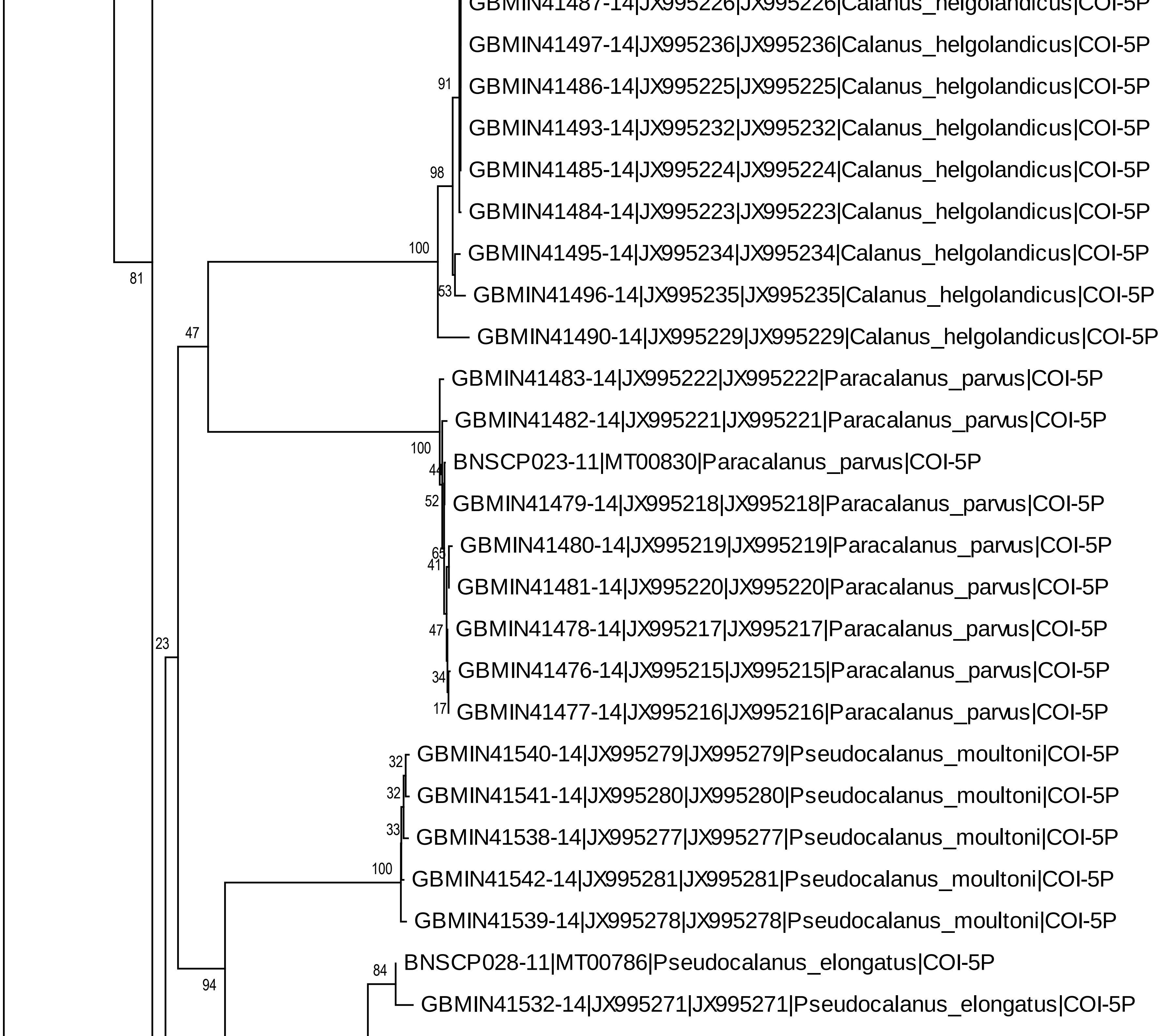
GBMIN41487-14|JX995226|JX995226|Calanus_helgolandicus|COI-5P
GBMIN41497-14|JX995236|JX995236|Calanus_helgolandicus|COI-5P
91
GBMIN41486-14|JX995225|JX995225|Calanus_helgolandicus|COI-5P
GBMIN41493-14|JX995232|JX995232|Calanus_helgolandicus|COI-5P
98
GBMIN41485-14|JX995224|JX995224|Calanus_helgolandicus|COI-5P
GBMIN41484-14|JX995223|JX995223|Calanus_helgolandicus|COI-5P
100
GBMIN41495-14|JX995234|JX995234|Calanus_helgolandicus|COI-5P
81
53
GBMIN41496-14|JX995235|JX995235|Calanus_helgolandicus|COI-5P
47
GBMIN41490-14|JX995229|JX995229|Calanus_helgolandicus|COI-5P
GBMIN41483-14|JX995222|JX995222|Paracalanus_parvus|COI-5P
GBMIN41482-14|JX995221|JX995221|Paracalanus_parvus|COI-5P
100
BNSCP023-11|MT00830|Paracalanus_parvus|COI-5P
44
52
GBMIN41479-14|JX995218|JX995218|Paracalanus_parvus|COI-5P
65
GBMIN41480-14|JX995219|JX995219|Paracalanus_parvus|COI-5P
41
GBMIN41481-14|JX995220|JX995220|Paracalanus_parvus|COI-5P
47
GBMIN41478-14|JX995217|JX995217|Paracalanus_parvus|COI-5P
23
34
GBMIN41476-14|JX995215|JX995215|Paracalanus_parvus|COI-5P
17
GBMIN41477-14|JX995216|JX995216|Paracalanus_parvus|COI-5P
32
GBMIN41540-14|JX995279|JX995279|Pseudocalanus_moultoni|COI-5P
32
GBMIN41541-14|JX995280|JX995280|Pseudocalanus_moultoni|COI-5P
33
GBMIN41538-14|JX995277|JX995277|Pseudocalanus_moultoni|COI-5P
100
GBMIN41542-14|JX995281|JX995281|Pseudocalanus_moultoni|COI-5P
GBMIN41539-14|JX995278|JX995278|Pseudocalanus_moultoni|COI-5P
84
BNSCP028-11|MT00786|Pseudocalanus_elongatus|COI-5P
94
GBMIN41532-14|JX995271|JX995271|Pseudocalanus_elongatus|COI-5P

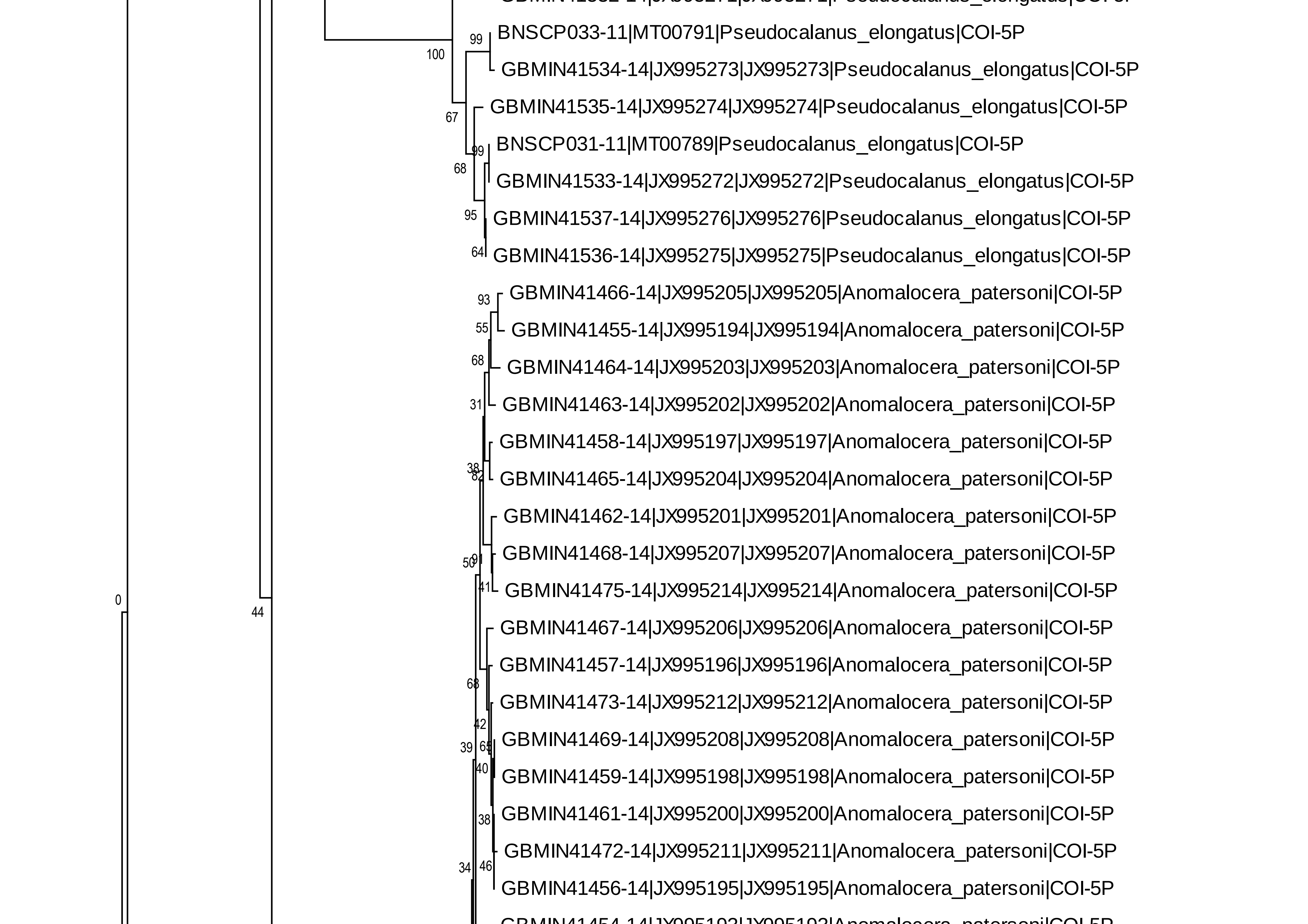
BNSCP033-11|MT00791|Pseudocalanus_elongatus|COI-5P
GBMIN41534-14|JX995273|JX995273|Pseudocalanus_elongatus|COI-5P
GBMIN41535-14|JX995274|JX995274|Pseudocalanus_elongatus|COI-5P
BNSCP031-11|MT00789|Pseudocalanus_elongatus|COI-5P
GBMIN41533-14|JX995272|JX995272|Pseudocalanus_elongatus|COI-5P
GBMIN41537-14|JX995276|JX995276|Pseudocalanus_elongatus|COI-5P
GBMIN41536-14|JX995275|JX995275|Pseudocalanus_elongatus|COI-5P
GBMIN41466-14|JX995205|JX995205|Anomalocera_patersoni|COI-5P
GBMIN41455-14|JX995194|JX995194|Anomalocera_patersoni|COI-5P
GBMIN41464-14|JX995203|JX995203|Anomalocera_patersoni|COI-5P
GBMIN41463-14|JX995202|JX995202|Anomalocera_patersoni|COI-5P
GBMIN41458-14|JX995197|JX995197|Anomalocera_patersoni|COI-5P
GBMIN41465-14|JX995204|JX995204|Anomalocera_patersoni|COI-5P
GBMIN41462-14|JX995201|JX995201|Anomalocera_patersoni|COI-5P
GBMIN41468-14|JX995207|JX995207|Anomalocera_patersoni|COI-5P
GBMIN41475-14|JX995214|JX995214|Anomalocera_patersoni|COI-5P
GBMIN41467-14|JX995206|JX995206|Anomalocera_patersoni|COI-5P
GBMIN41457-14|JX995196|JX995196|Anomalocera_patersoni|COI-5P
GBMIN41473-14|JX995212|JX995212|Anomalocera_patersoni|COI-5P
GBMIN41469-14|JX995208|JX995208|Anomalocera_patersoni|COI-5P
GBMIN41459-14|JX995198|JX995198|Anomalocera_patersoni|COI-5P
GBMIN41461-14|JX995200|JX995200|Anomalocera_patersoni|COI-5P
GBMIN41472-14|JX995211|JX995211|Anomalocera_patersoni|COI-5P
GBMIN41456-14|JX995195|JX995195|Anomalocera_patersoni|COI-5P

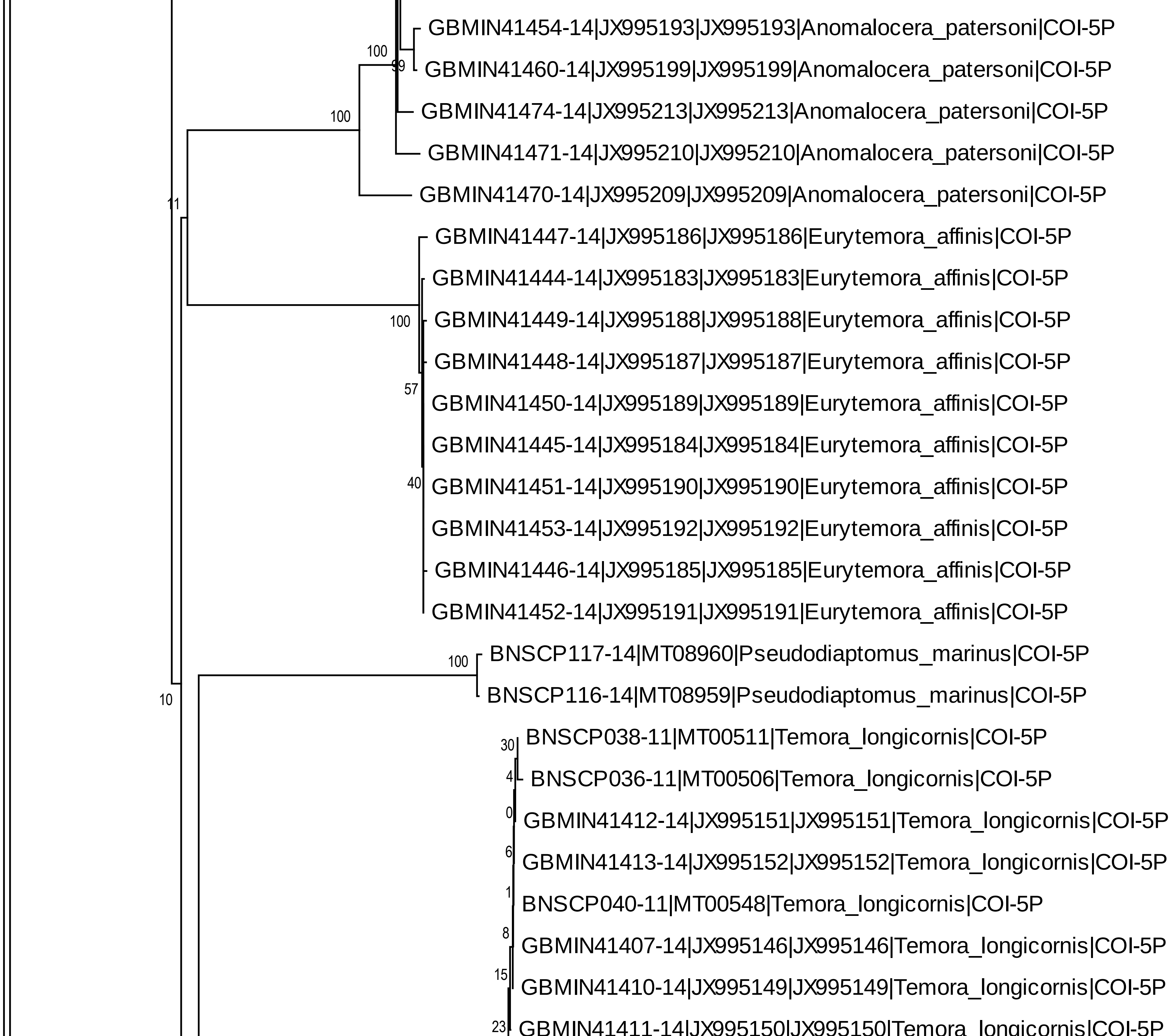
GBMIN41454-14|JX995193|JX995193|Anomalocera_patersoni|COI-5P
GBMIN41460-14|JX995199|JX995199|Anomalocera_patersoni|COI-5P
GBMIN41474-14|JX995213|JX995213|Anomalocera_patersoni|COI-5P
GBMIN41471-14|JX995210|JX995210|Anomalocera_patersoni|COI-5P
GBMIN41470-14|JX995209|JX995209|Anomalocera_patersoni|COI-5P
GBMIN41447-14|JX995186|JX995186|Eurytemora_affinis|COI-5P
GBMIN41444-14|JX995183|JX995183|Eurytemora_affinis|COI-5P
GBMIN41449-14|JX995188|JX995188|Eurytemora_affinis|COI-5P
GBMIN41448-14|JX995187|JX995187|Eurytemora_affinis|COI-5P
GBMIN41450-14|JX995189|JX995189|Eurytemora_affinis|COI-5P
GBMIN41445-14|JX995184|JX995184|Eurytemora_affinis|COI-5P
GBMIN41451-14|JX995190|JX995190|Eurytemora_affinis|COI-5P
GBMIN41453-14|JX995192|JX995192|Eurytemora_affinis|COI-5P
GBMIN41446-14|JX995185|JX995185|Eurytemora_affinis|COI-5P
GBMIN41452-14|JX995191|JX995191|Eurytemora_affinis|COI-5P
BNSCP117-14|MT08960|Pseudodiaptomus_marinus|COI-5P
BNSCP116-14|MT08959|Pseudodiaptomus_marinus|COI-5P
BNSCP038-11|MT00511|Temora_longicornis|COI-5P
BNSCP036-11|MT00506|Temora_longicornis|COI-5P
GBMIN41412-14|JX995151|JX995151|Temora_longicornis|COI-5P
GBMIN41413-14|JX995152|JX995152|Temora_longicornis|COI-5P
BNSCP040-11|MT00548|Temora_longicornis|COI-5P
GBMIN41407-14|JX995146|JX995146|Temora_longicornis|COI-5P
GBMIN41410-14|JX995149|JX995149|Temora_longicornis|COI-5P
GBMIN41411-14|JX995150|JX995150|Temora_longicornis|COI-5P

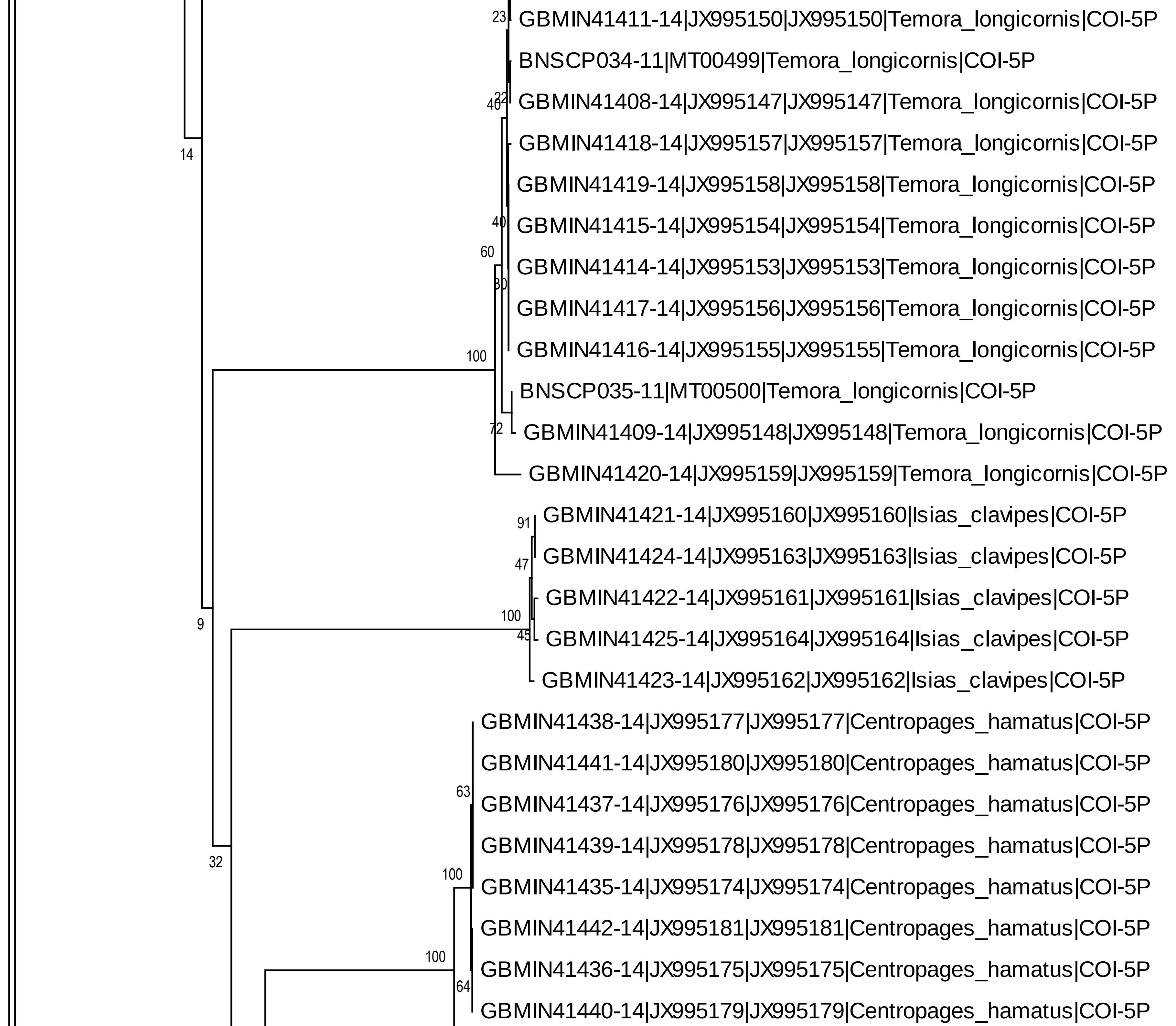

GBMIN41411-14|JX995150|JX995150|Temora_longicornis|COI-5P
BNSCP034-11|MT00499|Temora_longicornis|COI-5P
GBMIN41408-14|JX995147|JX995147|Temora_longicornis|COI-5P
GBMIN41418-14|JX995157|JX995157|Temora_longicornis|COI-5P
GBMIN41419-14|JX995158|JX995158|Temora_longicornis|COI-5P
GBMIN41415-14|JX995154|JX995154|Temora_longicornis|COI-5P
GBMIN41414-14|JX995153|JX995153|Temora_longicornis|COI-5P
GBMIN41417-14|JX995156|JX995156|Temora_longicornis|COI-5P
GBMIN41416-14|JX995155|JX995155|Temora_longicornis|COI-5P
BNSCP035-11|MT00500|Temora_longicornis|COI-5P
GBMIN41409-14|JX995148|JX995148|Temora_longicornis|COI-5P
GBMIN41420-14|JX995159|JX995159|Temora_longicornis|COI-5P
GBMIN41421-14|JX995160|JX995160|Isias_clavipes|COI-5P
GBMIN41424-14|JX995163|JX995163|Isias_clavipes|COI-5P
GBMIN41422-14|JX995161|JX995161|Isias_clavipes|COI-5P
GBMIN41425-14|JX995164|JX995164|Isias_clavipes|COI-5P
GBMIN41423-14|JX995162|JX995162|Isias_clavipes|COI-5P
GBMIN41438-14|JX995177|JX995177|Centropages_hamatus|COI-5P
GBMIN41441-14|JX995180|JX995180|Centropages_hamatus|COI-5P
GBMIN41437-14|JX995176|JX995176|Centropages_hamatus|COI-5P
GBMIN41439-14|JX995178|JX995178|Centropages_hamatus|COI-5P
GBMIN41435-14|JX995174|JX995174|Centropages_hamatus|COI-5P
GBMIN41442-14|JX995181|JX995181|Centropages_hamatus|COI-5P
GBMIN41436-14|JX995175|JX995175|Centropages_hamatus|COI-5P
GBMIN41440-14|JX995179|JX995179|Centropages_hamatus|COI-5P
23
22
40
14
40
60
30
100
72
91
47
100
45
9
32
63
100
100
64

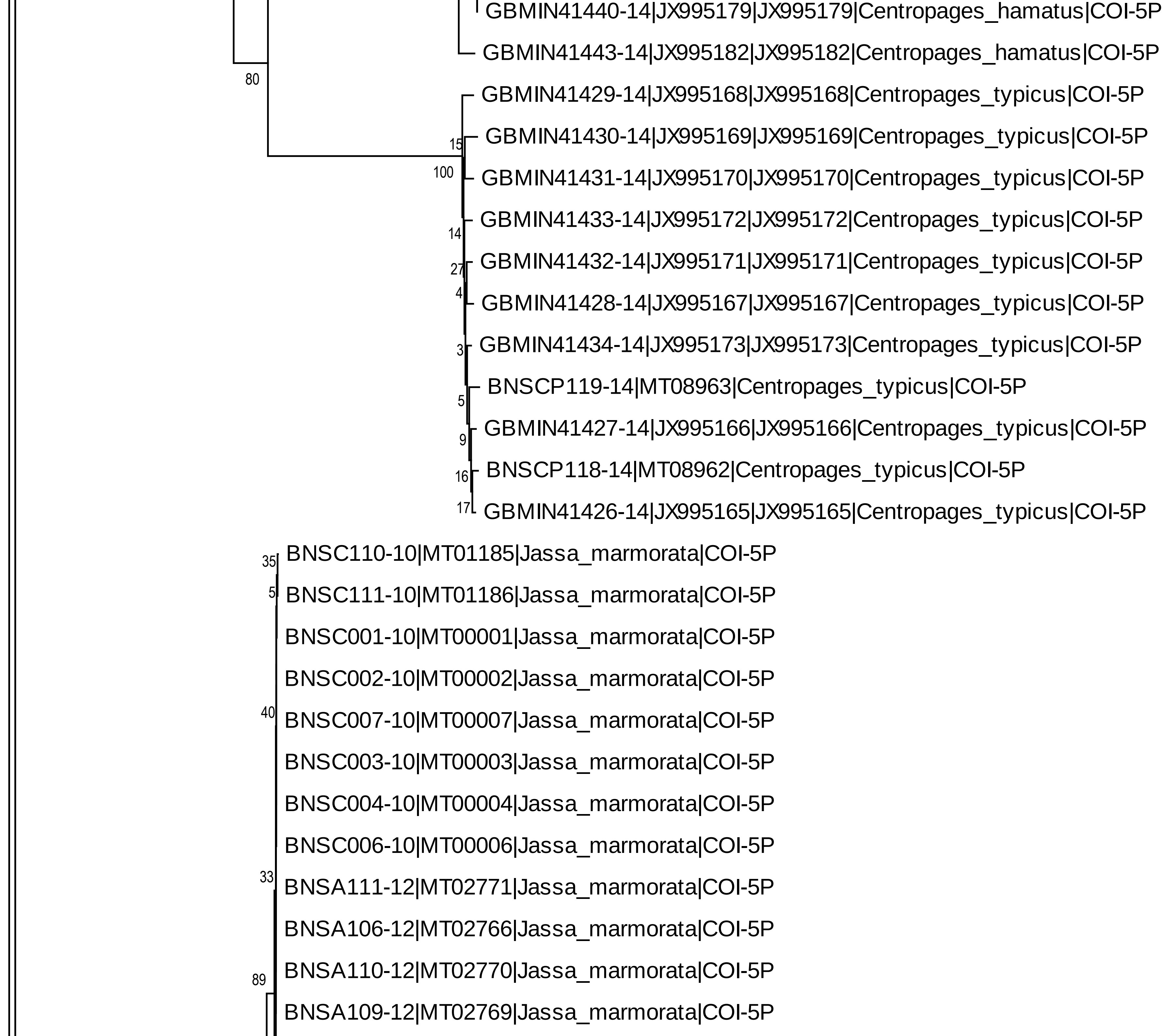
GBMIN41440-14|JX995179|JX995179|Centropages_hamatus|COI-5P
GBMIN41443-14|JX995182|JX995182|Centropages_hamatus|COI-5P
GBMIN41429-14|JX995168|JX995168|Centropages_typicus|COI-5P
GBMIN41430-14|JX995169|JX995169|Centropages_typicus|COI-5P
GBMIN41431-14|JX995170|JX995170|Centropages_typicus|COI-5P
GBMIN41433-14|JX995172|JX995172|Centropages_typicus|COI-5P
GBMIN41432-14|JX995171|JX995171|Centropages_typicus|COI-5P
GBMIN41428-14|JX995167|JX995167|Centropages_typicus|COI-5P
GBMIN41434-14|JX995173|JX995173|Centropages_typicus|COI-5P
BNSCP119-14|MT08963|Centropages_typicus|COI-5P
GBMIN41427-14|JX995166|JX995166|Centropages_typicus|COI-5P
BNSCP118-14|MT08962|Centropages_typicus|COI-5P
GBMIN41426-14|JX995165|JX995165|Centropages_typicus|COI-5P
BNSC110-10|MT01185|Jassa_marmorata|COI-5P
BNSC111-10|MT01186|Jassa_marmorata|COI-5P
BNSC001-10|MT00001|Jassa_marmorata|COI-5P
BNSC002-10|MT00002|Jassa_marmorata|COI-5P
BNSC007-10|MT00007|Jassa_marmorata|COI-5P
BNSC003-10|MT00003|Jassa_marmorata|COI-5P
BNSC004-10|MT00004|Jassa_marmorata|COI-5P
BNSC006-10|MT00006|Jassa_marmorata|COI-5P
BNSA111-12|MT02771|Jassa_marmorata|COI-5P
BNSA106-12|MT02766|Jassa_marmorata|COI-5P
BNSA110-12|MT02770|Jassa_marmorata|COI-5P
BNSA109-12|MT02769|Jassa_marmorata|COI-5P

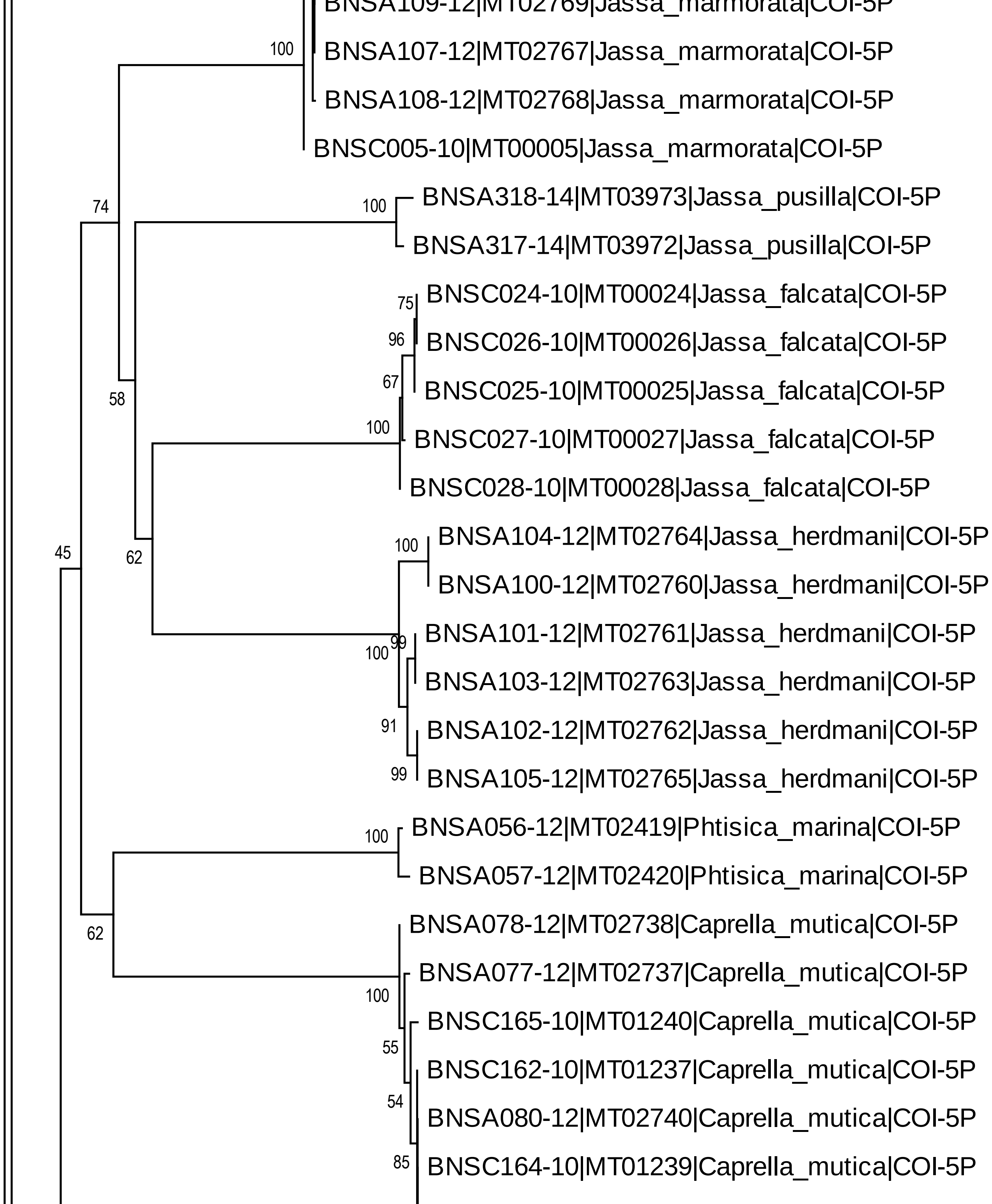

BNSA109-12|MT02769|Jassa_marmorata|COI-5P
BNSA107-12|MT02767|Jassa_marmorata|COI-5P
BNSA108-12|MT02768|Jassa_marmorata|COI-5P
BNSC005-10|MT00005|Jassa_marmorata|COI-5P
BNSA318-14|MT03973|Jassa_pusilla|COI-5P
BNSA317-14|MT03972|Jassa_pusilla|COI-5P
BNSC024-10|MT00024|Jassa_falcata|COI-5P
BNSC026-10|MT00026|Jassa_falcata|COI-5P
BNSC025-10|MT00025|Jassa_falcata|COI-5P
BNSC027-10|MT00027|Jassa_falcata|COI-5P
BNSC028-10|MT00028|Jassa_falcata|COI-5P
BNSA104-12|MT02764|Jassa_herdmani|COI-5P
BNSA100-12|MT02760|Jassa_herdmani|COI-5P
BNSA101-12|MT02761|Jassa_herdmani|COI-5P
BNSA103-12|MT02763|Jassa_herdmani|COI-5P
BNSA102-12|MT02762|Jassa_herdmani|COI-5P
BNSA105-12|MT02765|Jassa_herdmani|COI-5P
BNSA056-12|MT02419|Phtisica_marina|COI-5P
BNSA057-12|MT02420|Phtisica_marina|COI-5P
BNSA078-12|MT02738|Caprella_mutica|COI-5P
BNSA077-12|MT02737|Caprella_mutica|COI-5P
BNSC165-10|MT01240|Caprella_mutica|COI-5P
BNSC162-10|MT01237|Caprella_mutica|COI-5P
BNSA080-12|MT02740|Caprella_mutica|COI-5P
BNSC164-10|MT01239|Caprella_mutica|COI-5P

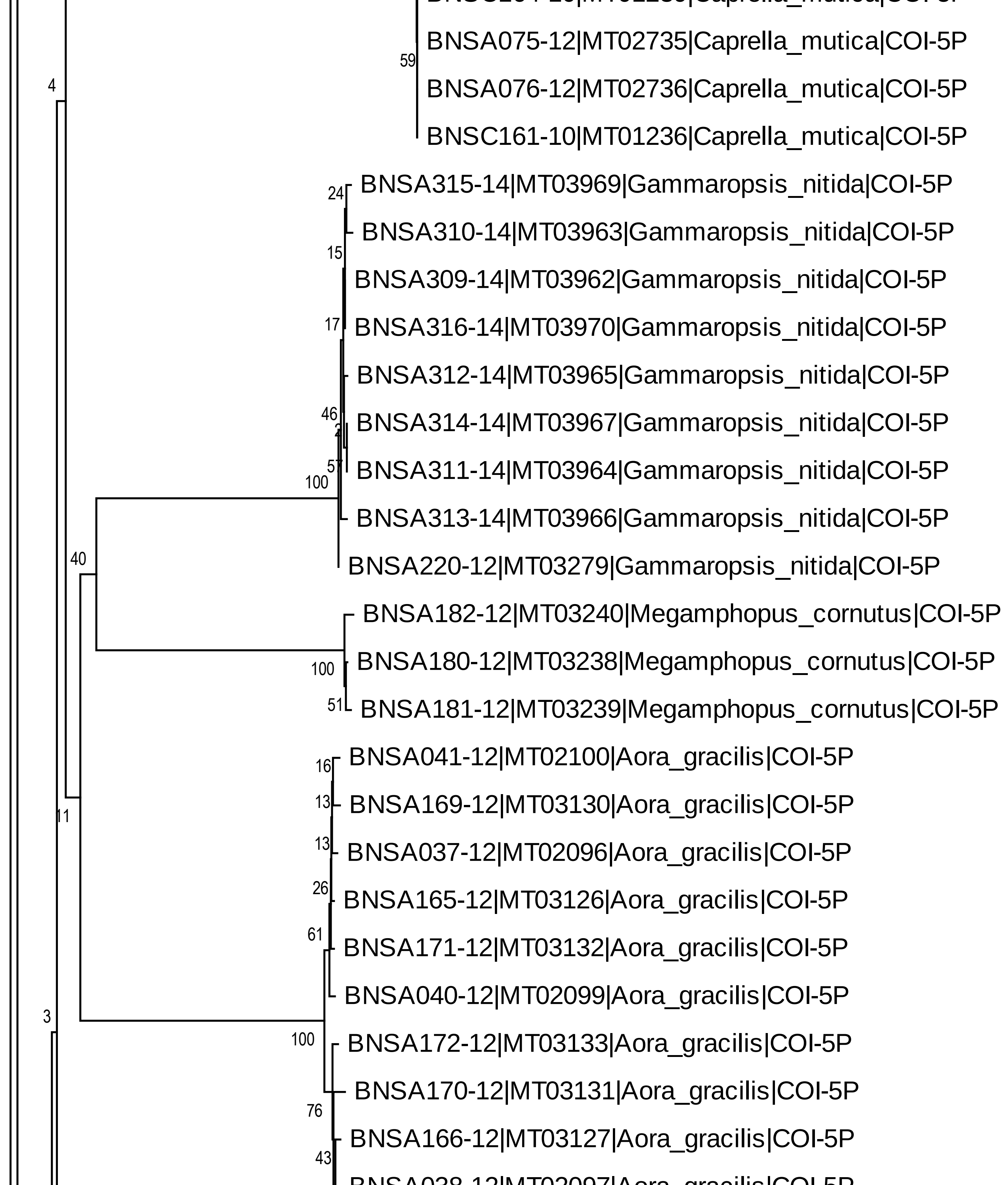
BNSA075-12|MT02735|Caprella_mutica|COI-5P
BNSA076-12|MT02736|Caprella_mutica|COI-5P
BNSC161-10|MT01236|Caprella_mutica|COI-5P
BNSA315-14|MT03969|Gammaropsis_nitida|COI-5P
BNSA310-14|MT03963|Gammaropsis_nitida|COI-5P
BNSA309-14|MT03962|Gammaropsis_nitida|COI-5P
BNSA316-14|MT03970|Gammaropsis_nitida|COI-5P
BNSA312-14|MT03965|Gammaropsis_nitida|COI-5P
BNSA314-14|MT03967|Gammaropsis_nitida|COI-5P
BNSA311-14|MT03964|Gammaropsis_nitida|COI-5P
BNSA313-14|MT03966|Gammaropsis_nitida|COI-5P
BNSA220-12|MT03279|Gammaropsis_nitida|COI-5P
BNSA182-12|MT03240|Megamphopus_cornutus|COI-5P
BNSA180-12|MT03238|Megamphopus_cornutus|COI-5P
BNSA181-12|MT03239|Megamphopus_cornutus|COI-5P
BNSA041-12|MT02100|Aora_gracilis|COI-5P
BNSA169-12|MT03130|Aora_gracilis|COI-5P
BNSA037-12|MT02096|Aora_gracilis|COI-5P
BNSA165-12|MT03126|Aora_gracilis|COI-5P
BNSA171-12|MT03132|Aora_gracilis|COI-5P
BNSA040-12|MT02099|Aora_gracilis|COI-5P
BNSA172-12|MT03133|Aora_gracilis|COI-5P
BNSA170-12|MT03131|Aora_gracilis|COI-5P
BNSA166-12|MT03127|Aora_gracilis|COI-5P

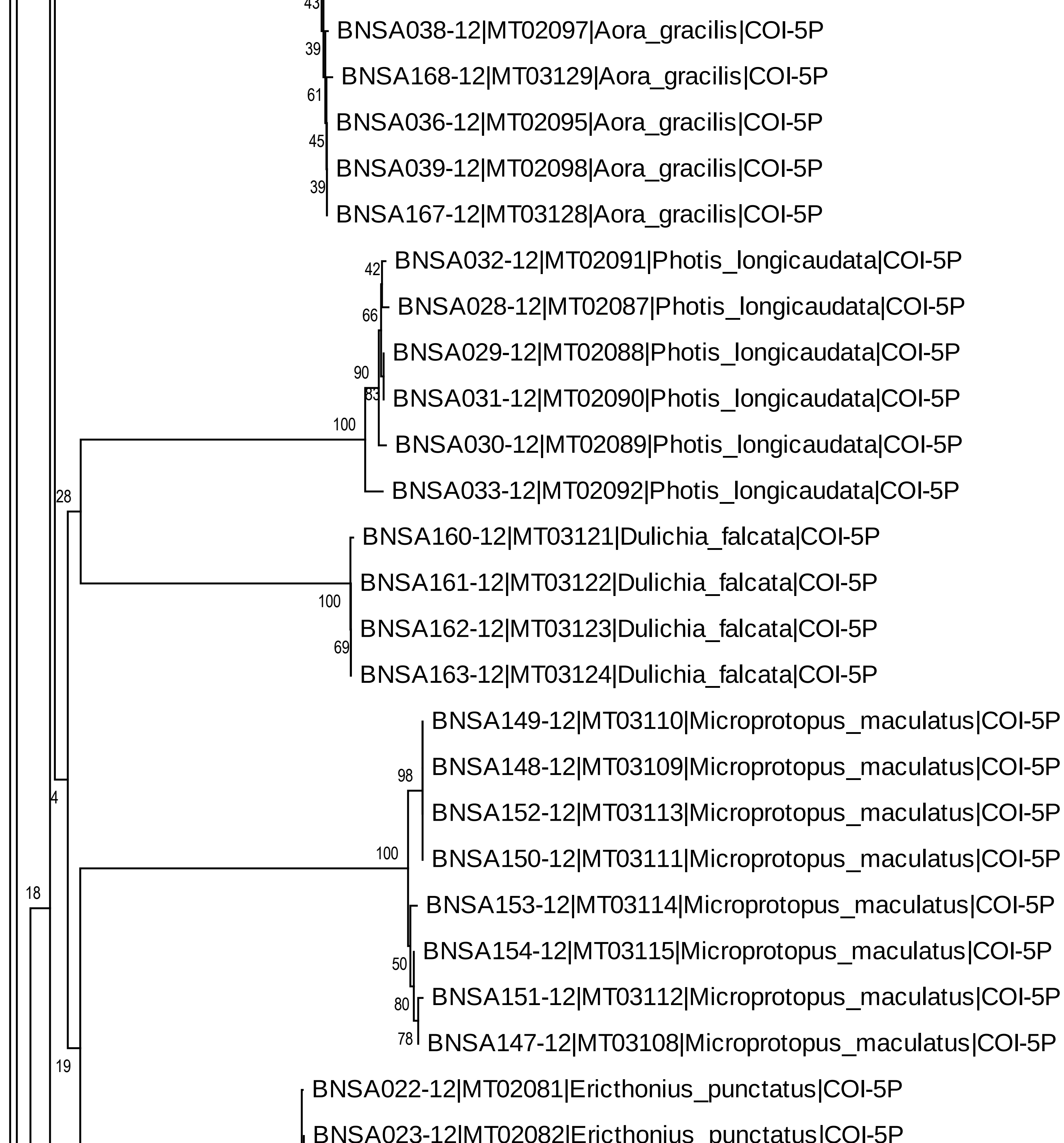

BNSA038-12|MT02097|Aora_gracilis|COI-5P
BNSA168-12|MT03129|Aora_gracilis|COI-5P
BNSA036-12|MT02095|Aora_gracilis|COI-5P
BNSA039-12|MT02098|Aora_gracilis|COI-5P
BNSA167-12|MT03128|Aora_gracilis|COI-5P
BNSA032-12|MT02091|Photis_longicaudata|COI-5P
BNSA028-12|MT02087|Photis_longicaudata|COI-5P
BNSA029-12|MT02088|Photis_longicaudata|COI-5P
BNSA031-12|MT02090|Photis_longicaudata|COI-5P
BNSA030-12|MT02089|Photis_longicaudata|COI-5P
BNSA033-12|MT02092|Photis_longicaudata|COI-5P
BNSA160-12|MT03121|Dulichia_falcata|COI-5P
BNSA161-12|MT03122|Dulichia_falcata|COI-5P
BNSA162-12|MT03123|Dulichia_falcata|COI-5P
BNSA163-12|MT03124|Dulichia_falcata|COI-5P
BNSA149-12|MT03110|Microprotopus_maculatus|COI-5P
BNSA148-12|MT03109|Microprotopus_maculatus|COI-5P
BNSA152-12|MT03113|Microprotopus_maculatus|COI-5P
BNSA150-12|MT03111|Microprotopus_maculatus|COI-5P
BNSA153-12|MT03114|Microprotopus_maculatus|COI-5P
BNSA154-12|MT03115|Microprotopus_maculatus|COI-5P
BNSA151-12|MT03112|Microprotopus_maculatus|COI-5P
BNSA147-12|MT03108|Microprotopus_maculatus|COI-5P
BNSA022-12|MT02081|Ericthonius_punctatus|COI-5P
BNSA023-12|MT02082|Ericthonius_punctatus|COI-5P

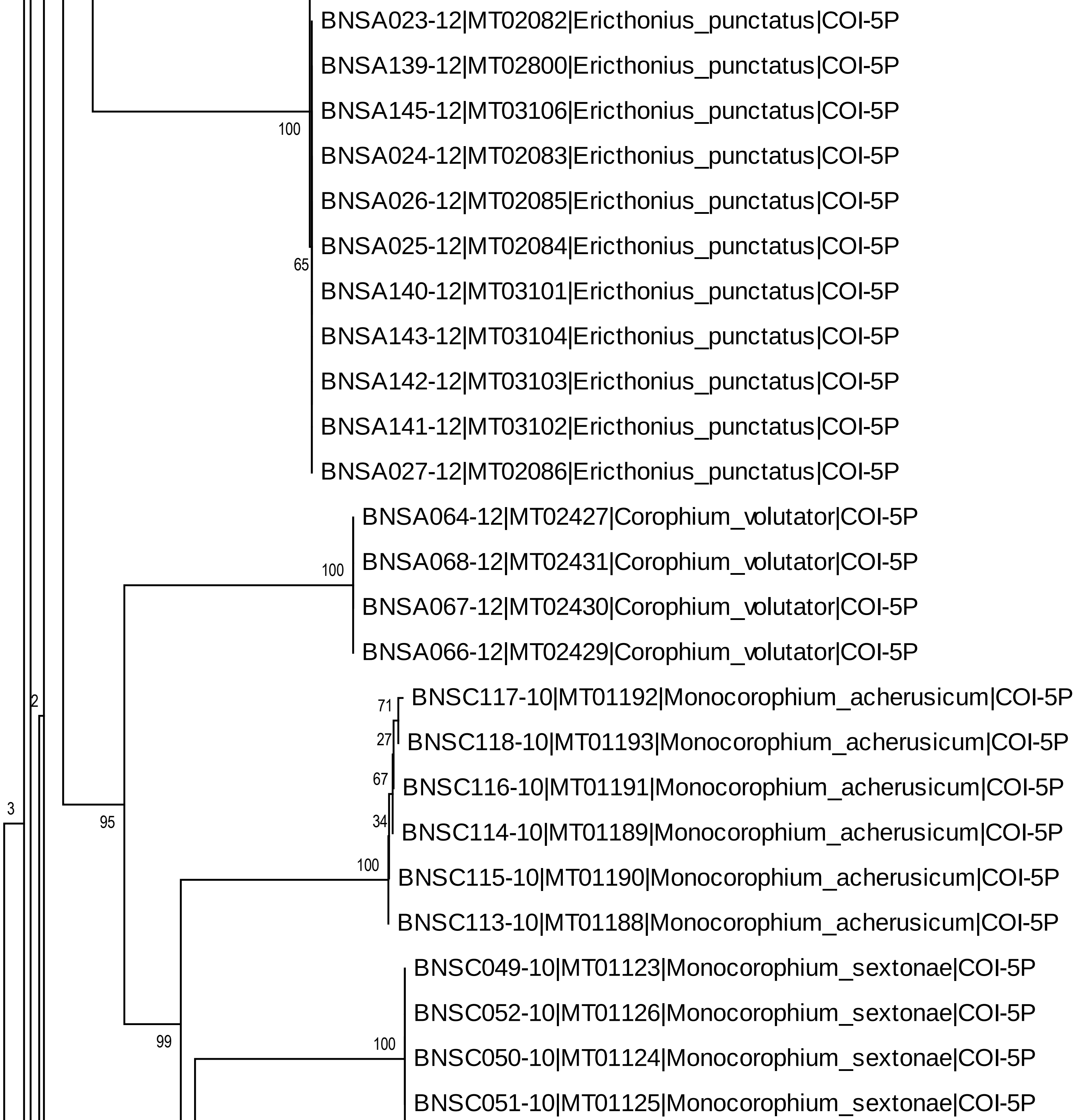
BNSA023-12|MT02082|Ericthonius_punctatus|COI-5P
BNSA139-12|MT02800|Ericthonius_punctatus|COI-5P
BNSA145-12|MT03106|Ericthonius_punctatus|COI-5P
100
BNSA024-12|MT02083|Ericthonius_punctatus|COI-5P
BNSA026-12|MT02085|Ericthonius_punctatus|COI-5P
BNSA025-12|MT02084|Ericthonius_punctatus|COI-5P
65
BNSA140-12|MT03101|Ericthonius_punctatus|COI-5P
BNSA143-12|MT03104|Ericthonius_punctatus|COI-5P
BNSA142-12|MT03103|Ericthonius_punctatus|COI-5P
BNSA141-12|MT03102|Ericthonius_punctatus|COI-5P
BNSA027-12|MT02086|Ericthonius_punctatus|COI-5P
BNSA064-12|MT02427|Corophium_volutator|COI-5P
100
BNSA068-12|MT02431|Corophium_volutator|COI-5P
BNSA067-12|MT02430|Corophium_volutator|COI-5P
BNSA066-12|MT02429|Corophium_volutator|COI-5P
71
BNSC117-10|MT01192|Monocorophium_acherusicum|COI-5P
2
27
BNSC118-10|MT01193|Monocorophium_acherusicum|COI-5P
67
BNSC116-10|MT01191|Monocorophium_acherusicum|COI-5P
3
95
34
BNSC114-10|MT01189|Monocorophium_acherusicum|COI-5P
100
BNSC115-10|MT01190|Monocorophium_acherusicum|COI-5P
BNSC113-10|MT01188|Monocorophium_acherusicum|COI-5P
BNSC049-10|MT01123|Monocorophium_sextonae|COI-5P
BNSC052-10|MT01126|Monocorophium_sextonae|COI-5P
99
100
BNSC050-10|MT01124|Monocorophium_sextonae|COI-5P
BNSC051-10|MT01125|Monocorophium_sextonae|COI-5P

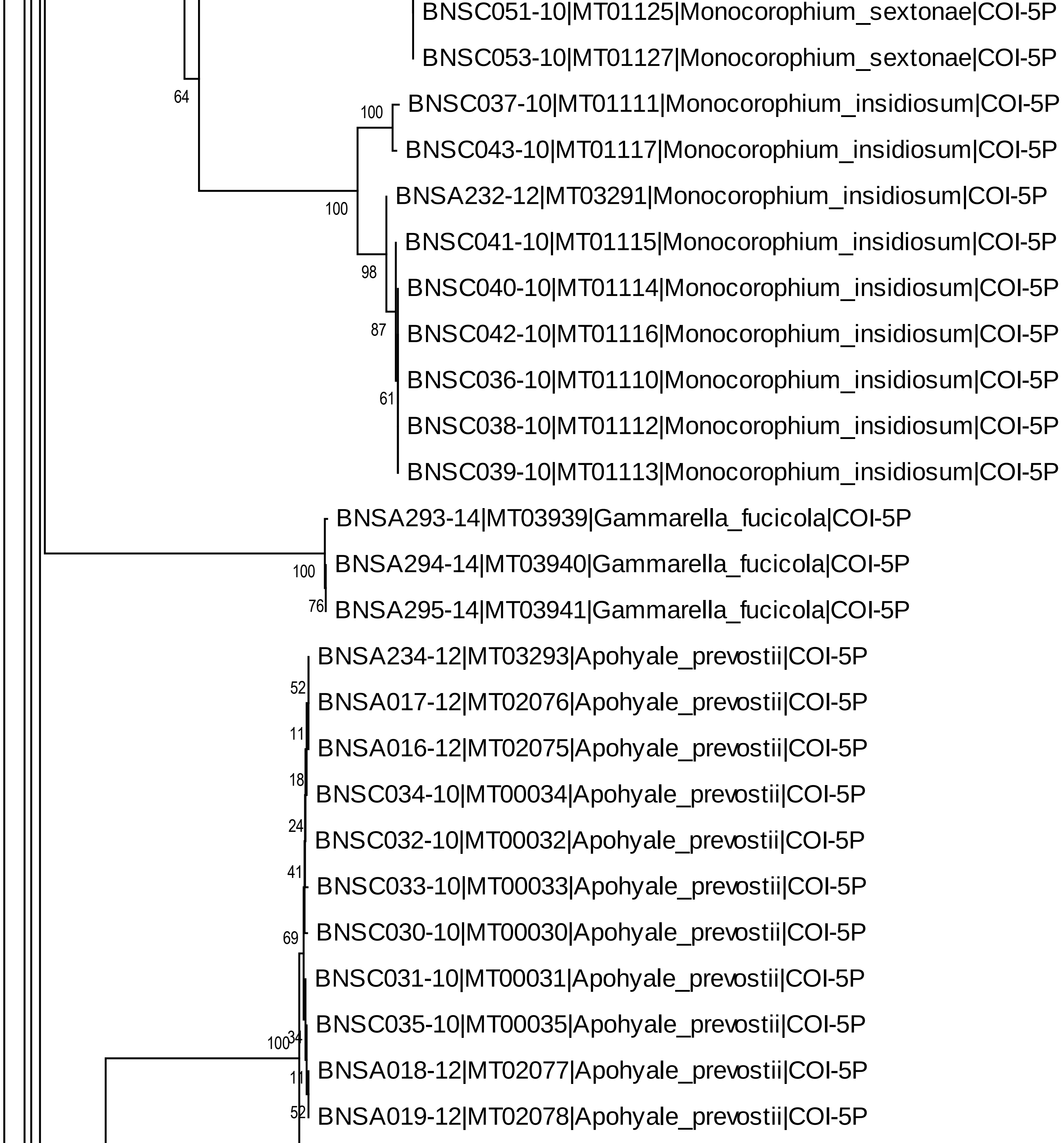
BNSC051-10|MT01125|Monocorophium_sextonae|COI-5P
BNSC053-10|MT01127|Monocorophium_sextonae|COI-5P
BNSC037-10|MT01111|Monocorophium_insidiosum|COI-5P
BNSC043-10|MT01117|Monocorophium_insidiosum|COI-5P
BNSA232-12|MT03291|Monocorophium_insidiosum|COI-5P
BNSC041-10|MT01115|Monocorophium_insidiosum|COI-5P
BNSC040-10|MT01114|Monocorophium_insidiosum|COI-5P
BNSC042-10|MT01116|Monocorophium_insidiosum|COI-5P
BNSC036-10|MT01110|Monocorophium_insidiosum|COI-5P
BNSC038-10|MT01112|Monocorophium_insidiosum|COI-5P
BNSC039-10|MT01113|Monocorophium_insidiosum|COI-5P
BNSA293-14|MT03939|Gammarella_fucicola|COI-5P
BNSA294-14|MT03940|Gammarella_fucicola|COI-5P
BNSA295-14|MT03941|Gammarella_fucicola|COI-5P
BNSA234-12|MT03293|Apohyale_prevostii|COI-5P
BNSA017-12|MT02076|Apohyale_prevostii|COI-5P
BNSA016-12|MT02075|Apohyale_prevostii|COI-5P
BNSC034-10|MT00034|Apohyale_prevostii|COI-5P
BNSC032-10|MT00032|Apohyale_prevostii|COI-5P
BNSC033-10|MT00033|Apohyale_prevostii|COI-5P
BNSC030-10|MT00030|Apohyale_prevostii|COI-5P
BNSC031-10|MT00031|Apohyale_prevostii|COI-5P
BNSC035-10|MT00035|Apohyale_prevostii|COI-5P
BNSA018-12|MT02077|Apohyale_prevostii|COI-5P
BNSA019-12|MT02078|Apohyale_prevostii|COI-5P

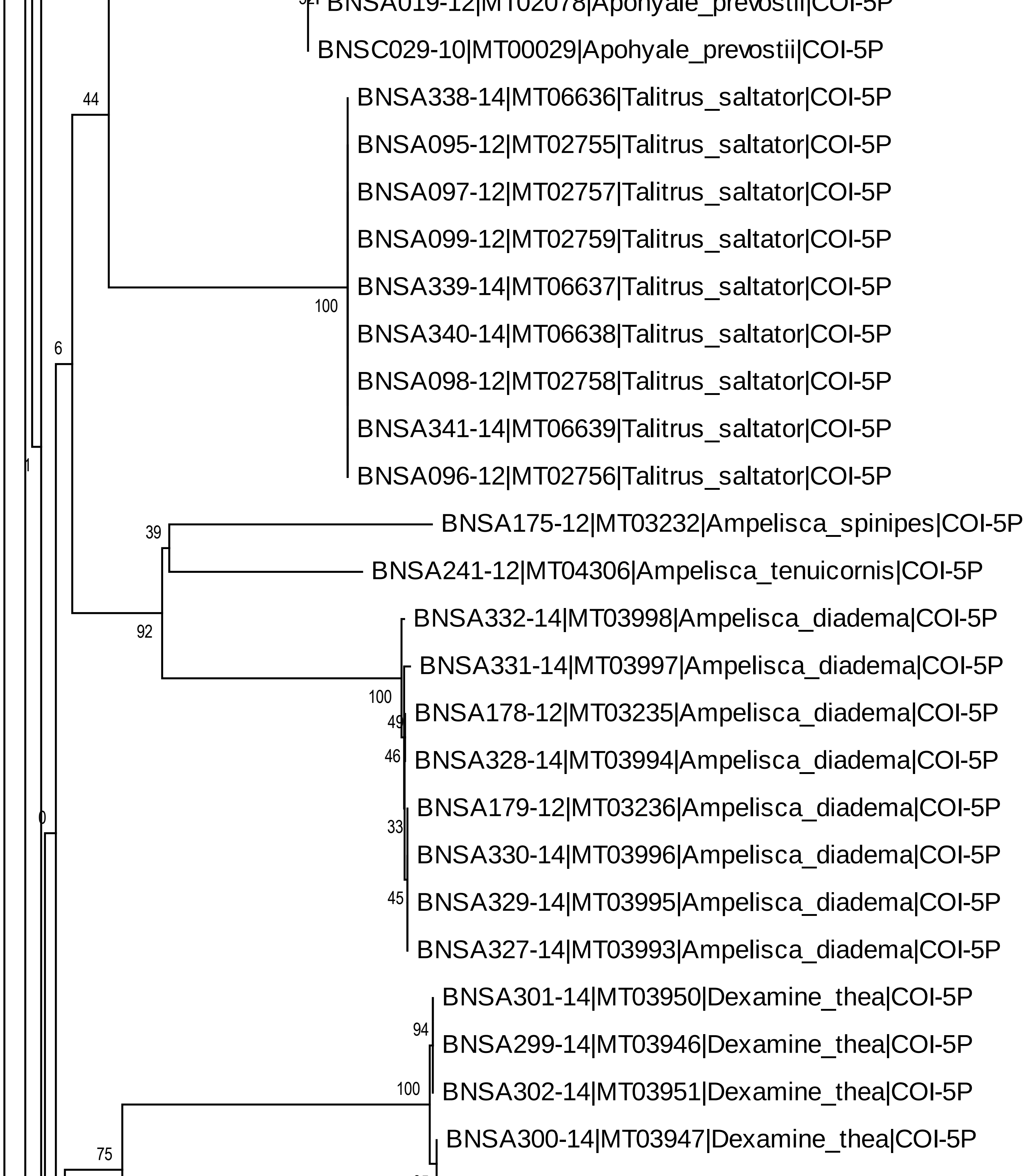
BNSA019-12|MT02078|Apohyale_prevostii|COI-5P
BNSC029-10|MT00029|Apohyale_prevostii|COI-5P
BNSA338-14|MT06636|Talitrus_saltator|COI-5P
BNSA095-12|MT02755|Talitrus_saltator|COI-5P
BNSA097-12|MT02757|Talitrus_saltator|COI-5P
BNSA099-12|MT02759|Talitrus_saltator|COI-5P
BNSA339-14|MT06637|Talitrus_saltator|COI-5P
BNSA340-14|MT06638|Talitrus_saltator|COI-5P
BNSA098-12|MT02758|Talitrus_saltator|COI-5P
BNSA341-14|MT06639|Talitrus_saltator|COI-5P
BNSA096-12|MT02756|Talitrus_saltator|COI-5P
BNSA175-12|MT03232|Ampelisca_spinipes|COI-5P
BNSA241-12|MT04306|Ampelisca_tenuicornis|COI-5P
BNSA332-14|MT03998|Ampelisca_diadema|COI-5P
BNSA331-14|MT03997|Ampelisca_diadema|COI-5P
BNSA178-12|MT03235|Ampelisca_diadema|COI-5P
BNSA328-14|MT03994|Ampelisca_diadema|COI-5P
BNSA179-12|MT03236|Ampelisca_diadema|COI-5P
BNSA330-14|MT03996|Ampelisca_diadema|COI-5P
BNSA329-14|MT03995|Ampelisca_diadema|COI-5P
BNSA327-14|MT03993|Ampelisca_diadema|COI-5P
BNSA301-14|MT03950|Dexamine_thea|COI-5P
BNSA299-14|MT03946|Dexamine_thea|COI-5P
BNSA302-14|MT03951|Dexamine_thea|COI-5P
BNSA300-14|MT03947|Dexamine_thea|COI-5P
44
6
1
0
100
39
92
100
49
46
33
45
94
100
75

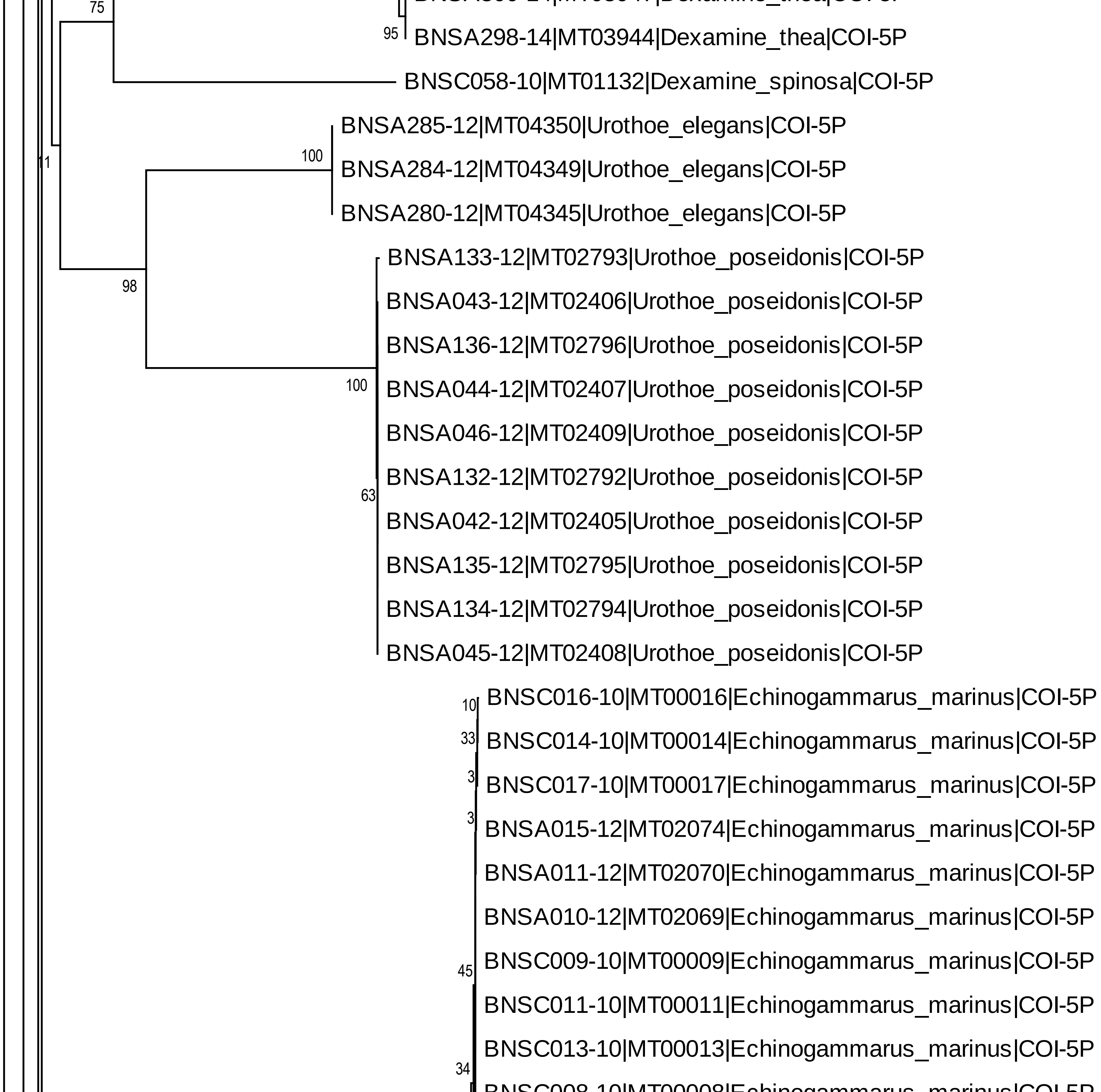
75
95
BNSA298-14|MT03944|Dexamine_thea|COI-5P
BNSC058-10|MT01132|Dexamine_spinosa|COI-5P
BNSA285-12|MT04350|Urothoe_elegans|COI-5P
11
100
BNSA284-12|MT04349|Urothoe_elegans|COI-5P
BNSA280-12|MT04345|Urothoe_elegans|COI-5P
BNSA133-12|MT02793|Urothoe_poseidonis|COI-5P
98
BNSA043-12|MT02406|Urothoe_poseidonis|COI-5P
BNSA136-12|MT02796|Urothoe_poseidonis|COI-5P
100
BNSA044-12|MT02407|Urothoe_poseidonis|COI-5P
BNSA046-12|MT02409|Urothoe_poseidonis|COI-5P
BNSA132-12|MT02792|Urothoe_poseidonis|COI-5P
63
BNSA042-12|MT02405|Urothoe_poseidonis|COI-5P
BNSA135-12|MT02795|Urothoe_poseidonis|COI-5P
BNSA134-12|MT02794|Urothoe_poseidonis|COI-5P
BNSA045-12|MT02408|Urothoe_poseidonis|COI-5P
10
BNSC016-10|MT00016|Echinogammarus_marinus|COI-5P
33
BNSC014-10|MT00014|Echinogammarus_marinus|COI-5P
3
BNSC017-10|MT00017|Echinogammarus_marinus|COI-5P
3
BNSA015-12|MT02074|Echinogammarus_marinus|COI-5P
BNSA011-12|MT02070|Echinogammarus_marinus|COI-5P
BNSA010-12|MT02069|Echinogammarus_marinus|COI-5P
BNSC009-10|MT00009|Echinogammarus_marinus|COI-5P
45
BNSC011-10|MT00011|Echinogammarus_marinus|COI-5P
BNSC013-10|MT00013|Echinogammarus_marinus|COI-5P
34

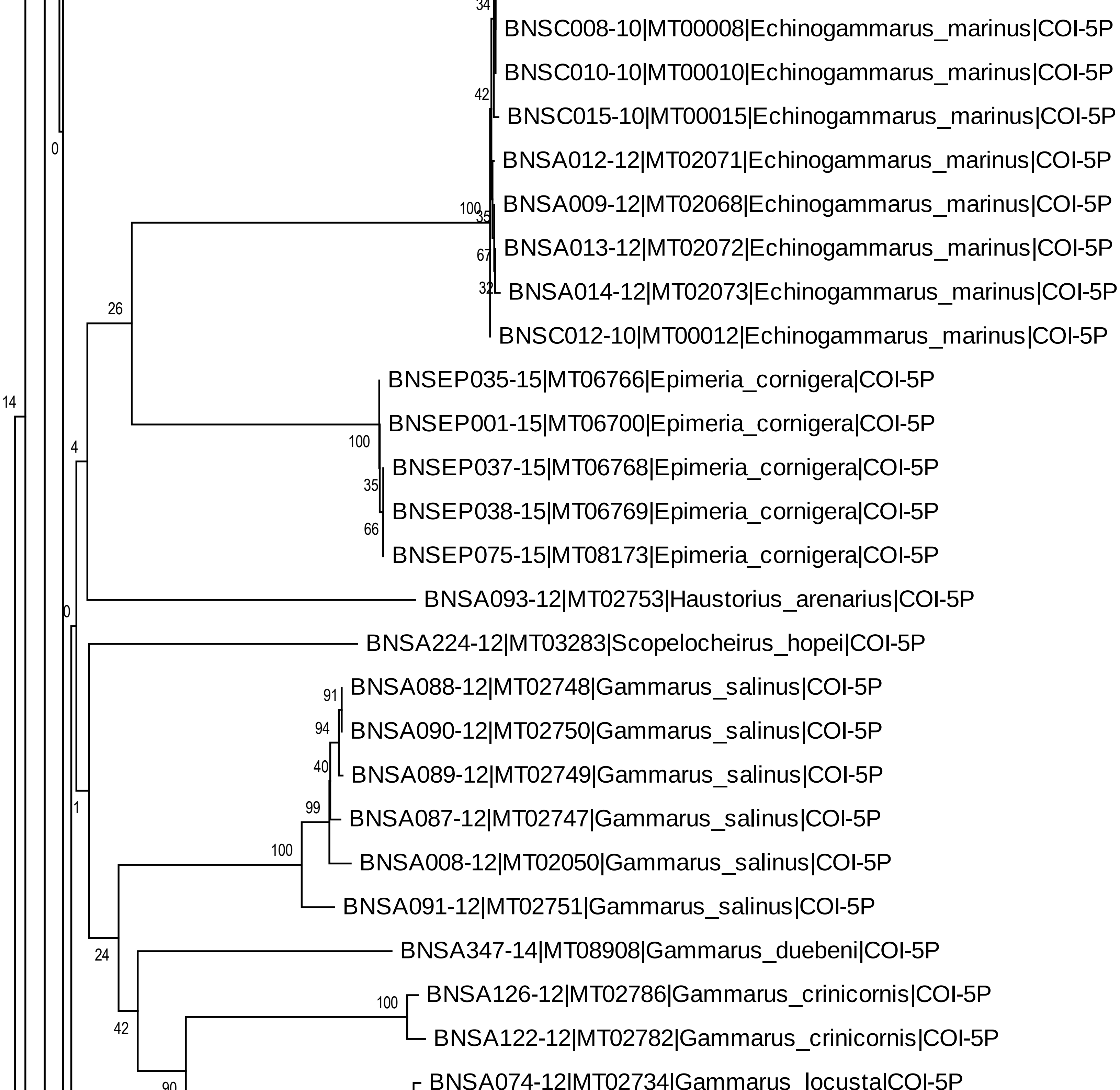
34
BNSC008-10|MT00008|Echinogammarus_marinus|COI-5P
BNSC010-10|MT00010|Echinogammarus_marinus|COI-5P
42
BNSC015-10|MT00015|Echinogammarus_marinus|COI-5P
0
BNSA012-12|MT02071|Echinogammarus_marinus|COI-5P
100
35
BNSA009-12|MT02068|Echinogammarus_marinus|COI-5P
67
BNSA013-12|MT02072|Echinogammarus_marinus|COI-5P
32
BNSA014-12|MT02073|Echinogammarus_marinus|COI-5P
26
BNSC012-10|MT00012|Echinogammarus_marinus|COI-5P
BNSEP035-15|MT06766|Epimeria_cornigera|COI-5P
14
BNSEP001-15|MT06700|Epimeria_cornigera|COI-5P
100
4
BNSEP037-15|MT06768|Epimeria_cornigera|COI-5P
35
BNSEP038-15|MT06769|Epimeria_cornigera|COI-5P
66
BNSEP075-15|MT08173|Epimeria_cornigera|COI-5P
BNSA093-12|MT02753|Haustorius_arenarius|COI-5P
0
BNSA224-12|MT03283|Scopelocheirus_hopei|COI-5P
91
BNSA088-12|MT02748|Gammarus_salinus|COI-5P
94
BNSA090-12|MT02750|Gammarus_salinus|COI-5P
40
BNSA089-12|MT02749|Gammarus_salinus|COI-5P
1
99
BNSA087-12|MT02747|Gammarus_salinus|COI-5P
100
BNSA008-12|MT02050|Gammarus_salinus|COI-5P
BNSA091-12|MT02751|Gammarus_salinus|COI-5P
24
BNSA347-14|MT08908|Gammarus_duebeni|COI-5P
100
BNSA126-12|MT02786|Gammarus_crinicornis|COI-5P
42
BNSA122-12|MT02782|Gammarus_crinicornis|COI-5P
90
BNSA074-12|MT02734|Gammarus_locusta|COI-5P

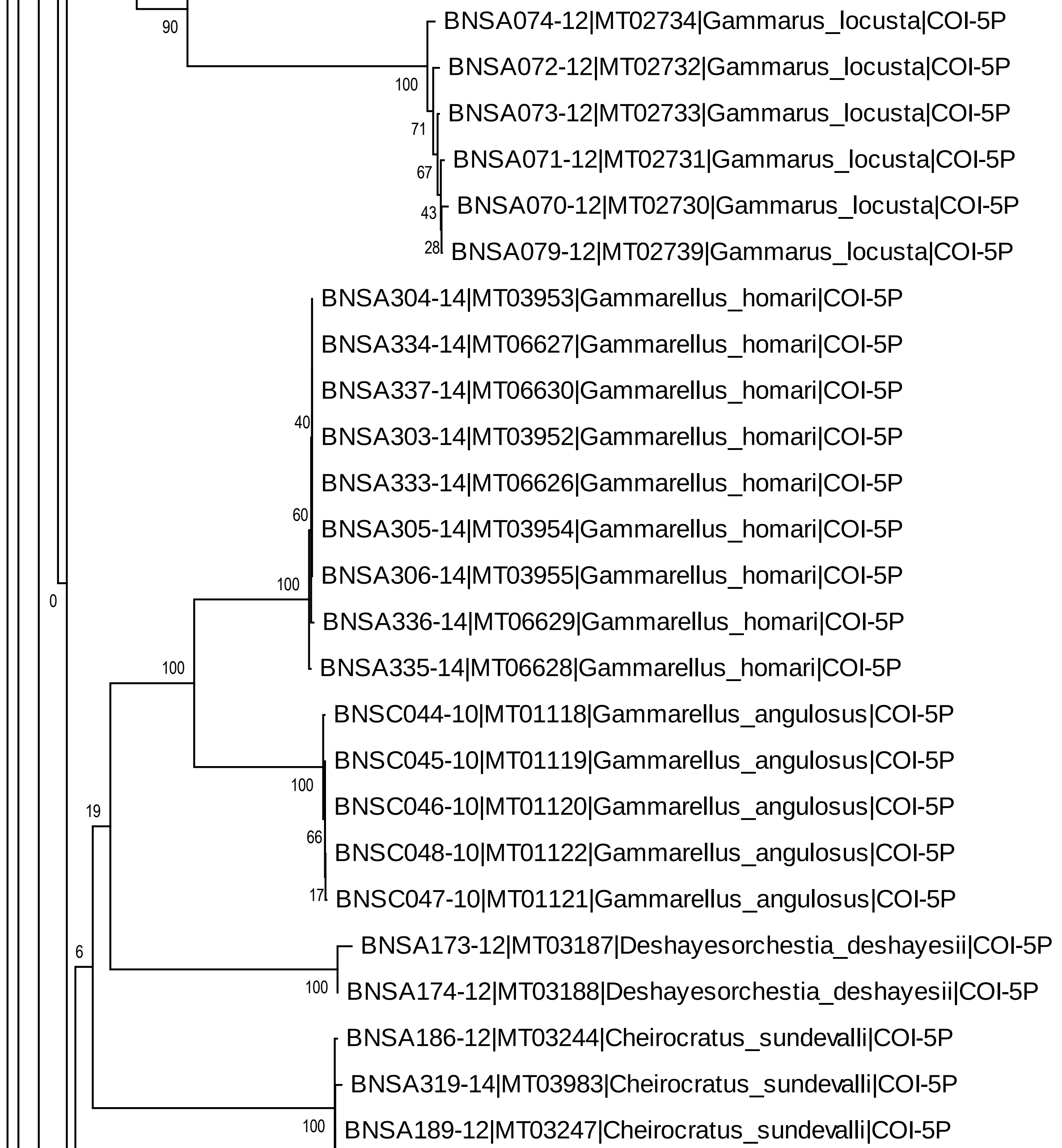

90
BNSA074-12|MT02734|Gammarus_locusta|COI-5P
BNSA072-12|MT02732|Gammarus_locusta|COI-5P
100
BNSA073-12|MT02733|Gammarus_locusta|COI-5P
71
BNSA071-12|MT02731|Gammarus_locusta|COI-5P
67
BNSA070-12|MT02730|Gammarus_locusta|COI-5P
43
28
BNSA079-12|MT02739|Gammarus_locusta|COI-5P
BNSA304-14|MT03953|Gammarellus_homari|COI-5P
BNSA334-14|MT06627|Gammarellus_homari|COI-5P
BNSA337-14|MT06630|Gammarellus_homari|COI-5P
40
BNSA303-14|MT03952|Gammarellus_homari|COI-5P
BNSA333-14|MT06626|Gammarellus_homari|COI-5P
60
BNSA305-14|MT03954|Gammarellus_homari|COI-5P
BNSA306-14|MT03955|Gammarellus_homari|COI-5P
100
0
BNSA336-14|MT06629|Gammarellus_homari|COI-5P
100
BNSA335-14|MT06628|Gammarellus_homari|COI-5P
BNSC044-10|MT01118|Gammarellus_angulosus|COI-5P
BNSC045-10|MT01119|Gammarellus_angulosus|COI-5P
100
BNSC046-10|MT01120|Gammarellus_angulosus|COI-5P
19
66
BNSC048-10|MT01122|Gammarellus_angulosus|COI-5P
17
BNSC047-10|MT01121|Gammarellus_angulosus|COI-5P
BNSA173-12|MT03187|Deshayesorchestia_deshayesii|COI-5P
6
100
BNSA174-12|MT03188|Deshayesorchestia_deshayesii|COI-5P
BNSA186-12|MT03244|Cheirocratus_sundevalli|COI-5P
BNSA319-14|MT03983|Cheirocratus_sundevalli|COI-5P
100
BNSA189-12|MT03247|Cheirocratus_sundevalli|COI-5P

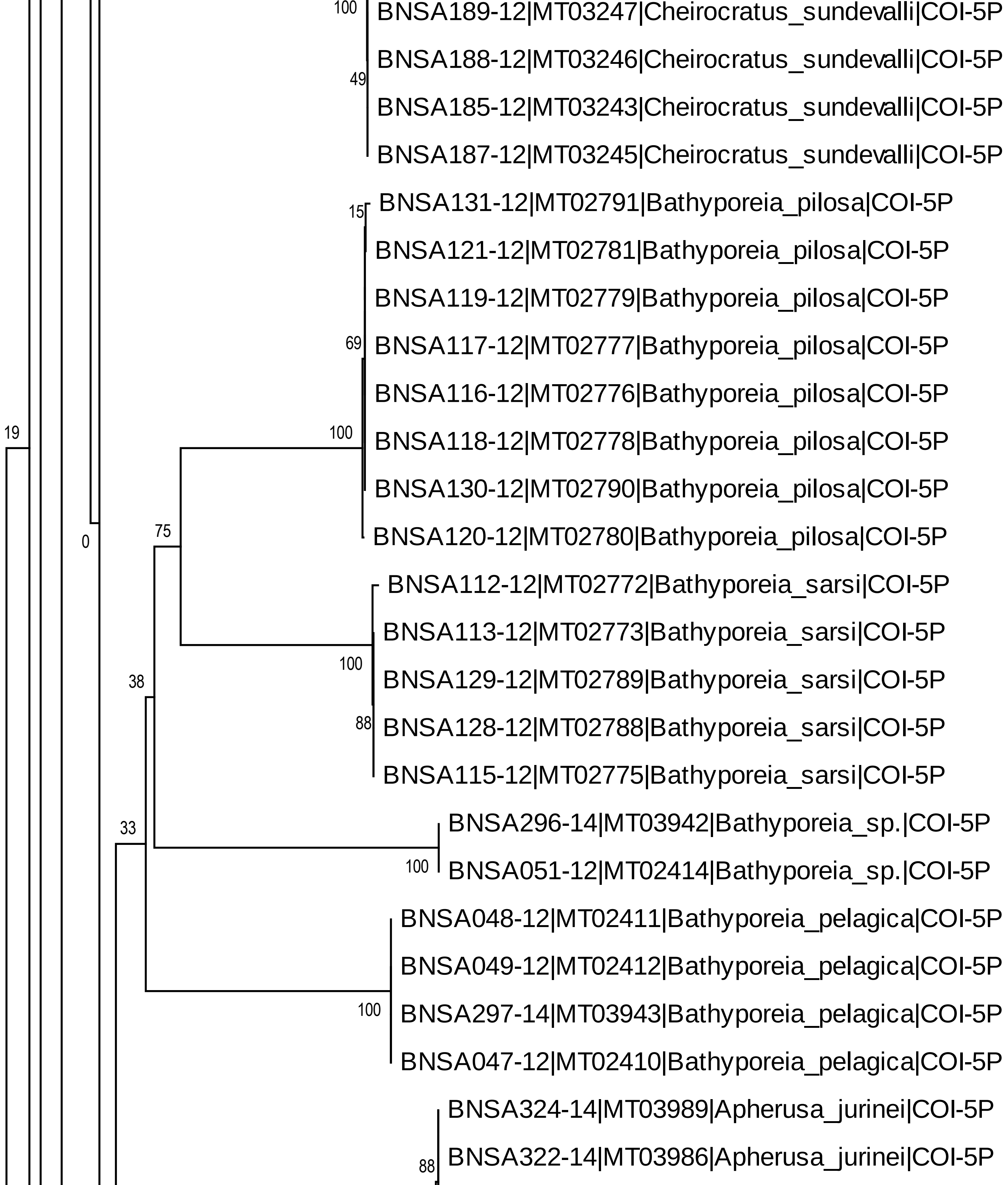
100
BNSA189-12|MT03247|Cheirocratus_sundevalli|COI-5P
BNSA188-12|MT03246|Cheirocratus_sundevalli|COI-5P
49
BNSA185-12|MT03243|Cheirocratus_sundevalli|COI-5P
BNSA187-12|MT03245|Cheirocratus_sundevalli|COI-5P
15
BNSA131-12|MT02791|Bathyporeia_pilosa|COI-5P
BNSA121-12|MT02781|Bathyporeia_pilosa|COI-5P
BNSA119-12|MT02779|Bathyporeia_pilosa|COI-5P
69
BNSA117-12|MT02777|Bathyporeia_pilosa|COI-5P
BNSA116-12|MT02776|Bathyporeia_pilosa|COI-5P
19
100
BNSA118-12|MT02778|Bathyporeia_pilosa|COI-5P
BNSA130-12|MT02790|Bathyporeia_pilosa|COI-5P
75
0
BNSA120-12|MT02780|Bathyporeia_pilosa|COI-5P
BNSA112-12|MT02772|Bathyporeia_sarsi|COI-5P
BNSA113-12|MT02773|Bathyporeia_sarsi|COI-5P
100
38
BNSA129-12|MT02789|Bathyporeia_sarsi|COI-5P
88
BNSA128-12|MT02788|Bathyporeia_sarsi|COI-5P
BNSA115-12|MT02775|Bathyporeia_sarsi|COI-5P
33
BNSA296-14|MT03942|Bathyporeia_sp.|COI-5P
100
BNSA051-12|MT02414|Bathyporeia_sp.|COI-5P
BNSA048-12|MT02411|Bathyporeia_pelagica|COI-5P
BNSA049-12|MT02412|Bathyporeia_pelagica|COI-5P
100
BNSA297-14|MT03943|Bathyporeia_pelagica|COI-5P
BNSA047-12|MT02410|Bathyporeia_pelagica|COI-5P
BNSA324-14|MT03989|Apherusa_jurinei|COI-5P
BNSA322-14|MT03986|Apherusa_jurinei|COI-5P
88

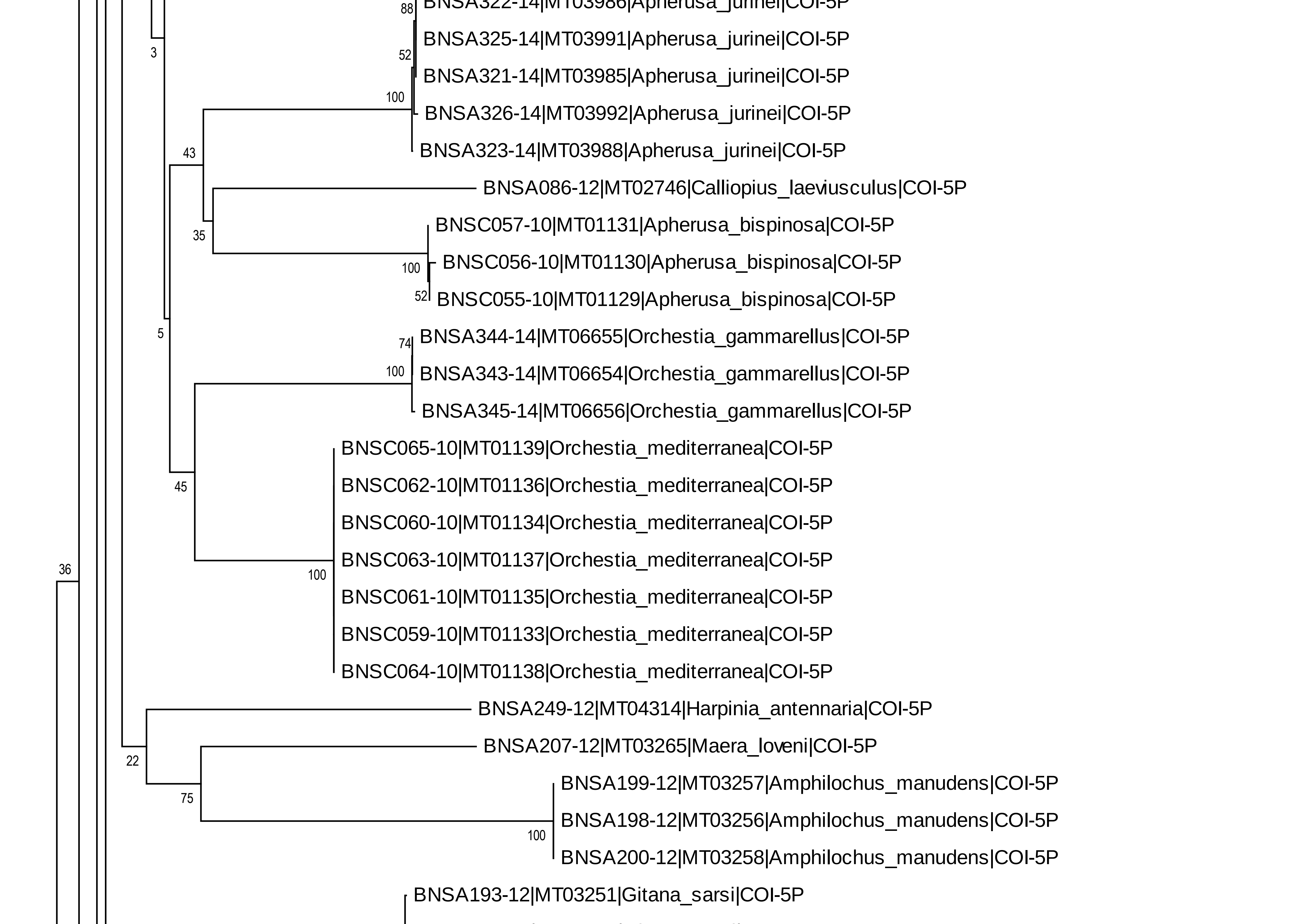

88
BNSA325-14|MT03991|Apherusa_jurinei|COI-5P
3
52
BNSA321-14|MT03985|Apherusa_jurinei|COI-5P
100
BNSA326-14|MT03992|Apherusa_jurinei|COI-5P
BNSA323-14|MT03988|Apherusa_jurinei|COI-5P
43
BNSA086-12|MT02746|Calliopius_laeviusculus|COI-5P
BNSC057-10|MT01131|Apherusa_bispinosa|COI-5P
35
100
BNSC056-10|MT01130|Apherusa_bispinosa|COI-5P
52
BNSC055-10|MT01129|Apherusa_bispinosa|COI-5P
5
74
BNSA344-14|MT06655|Orchestia_gammarellus|COI-5P
100
BNSA343-14|MT06654|Orchestia_gammarellus|COI-5P
BNSA345-14|MT06656|Orchestia_gammarellus|COI-5P
BNSC065-10|MT01139|Orchestia_mediterranea|COI-5P
45
BNSC062-10|MT01136|Orchestia_mediterranea|COI-5P
BNSC060-10|MT01134|Orchestia_mediterranea|COI-5P
36
BNSC063-10|MT01137|Orchestia_mediterranea|COI-5P
100
BNSC061-10|MT01135|Orchestia_mediterranea|COI-5P
BNSC059-10|MT01133|Orchestia_mediterranea|COI-5P
BNSC064-10|MT01138|Orchestia_mediterranea|COI-5P
BNSA249-12|MT04314|Harpinia_antennaria|COI-5P
BNSA207-12|MT03265|Maera_loveni|COI-5P
22
BNSA199-12|MT03257|Amphilochus_manudens|COI-5P
75
BNSA198-12|MT03256|Amphilochus_manudens|COI-5P
100
BNSA200-12|MT03258|Amphilochus_manudens|COI-5P
BNSA193-12|MT03251|Gitana_sarsi|COI-5P

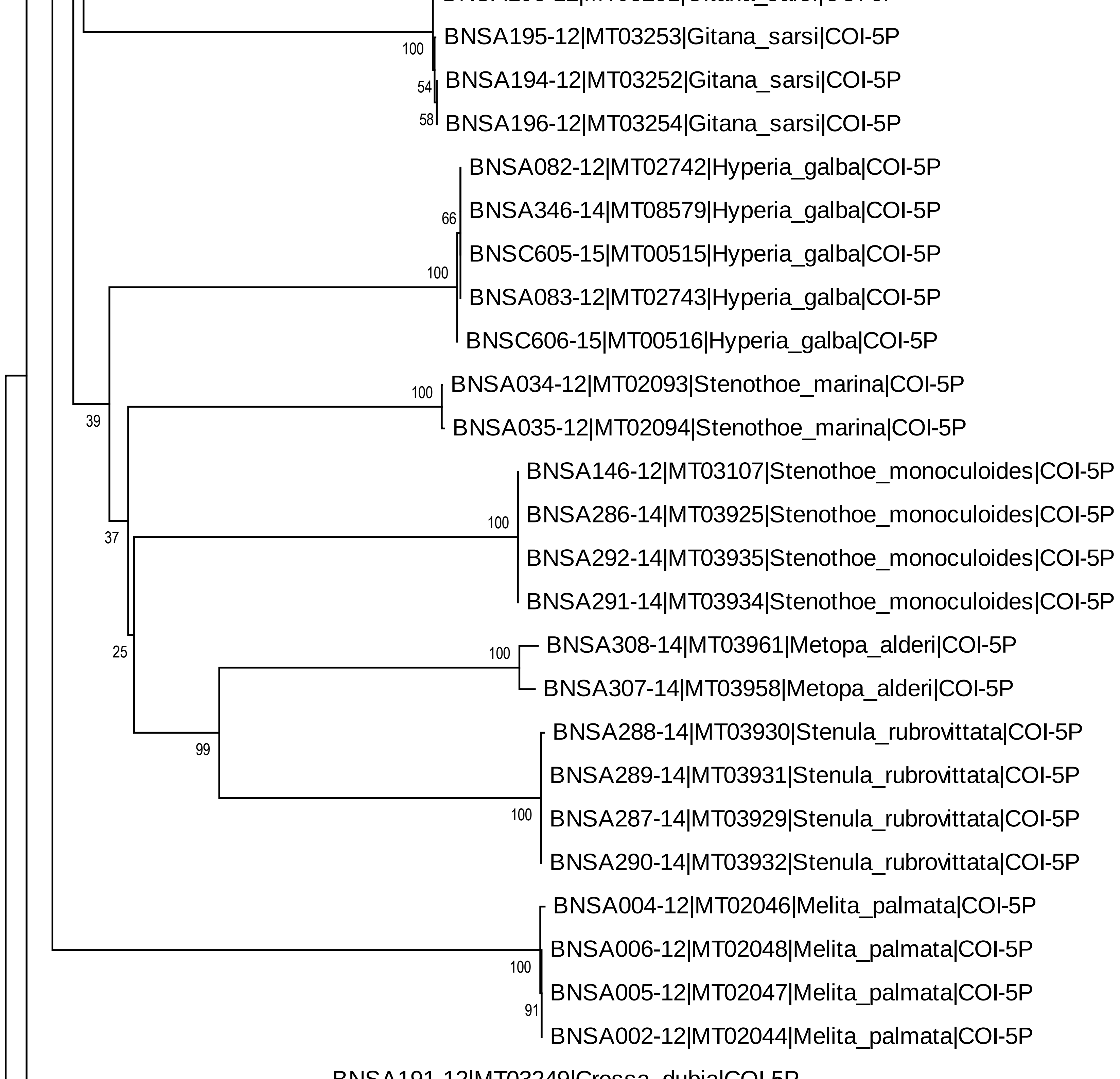

BNSA195-12|MT03253|Gitana_sarsi|COI-5P
100
BNSA194-12|MT03252|Gitana_sarsi|COI-5P
54
BNSA196-12|MT03254|Gitana_sarsi|COI-5P
58
BNSA082-12|MT02742|Hyperia_galba|COI-5P
BNSA346-14|MT08579|Hyperia_galba|COI-5P
66
BNSC605-15|MT00515|Hyperia_galba|COI-5P
100
BNSA083-12|MT02743|Hyperia_galba|COI-5P
BNSC606-15|MT00516|Hyperia_galba|COI-5P
BNSA034-12|MT02093|Stenothoe_marina|COI-5P
100
BNSA035-12|MT02094|Stenothoe_marina|COI-5P
39
BNSA146-12|MT03107|Stenothoe_monoculoides|COI-5P
BNSA286-14|MT03925|Stenothoe_monoculoides|COI-5P
100
BNSA292-14|MT03935|Stenothoe_monoculoides|COI-5P
BNSA291-14|MT03934|Stenothoe_monoculoides|COI-5P
37
BNSA308-14|MT03961|Metopa_alderi|COI-5P
100
BNSA307-14|MT03958|Metopa_alderi|COI-5P
25
BNSA288-14|MT03930|Stenula_rubrovittata|COI-5P
99
BNSA289-14|MT03931|Stenula_rubrovittata|COI-5P
BNSA287-14|MT03929|Stenula_rubrovittata|COI-5P
100
BNSA290-14|MT03932|Stenula_rubrovittata|COI-5P
BNSA004-12|MT02046|Melita_palmata|COI-5P
BNSA006-12|MT02048|Melita_palmata|COI-5P
100
BNSA005-12|MT02047|Melita_palmata|COI-5P
91
BNSA002-12|MT02044|Melita_palmata|COI-5P

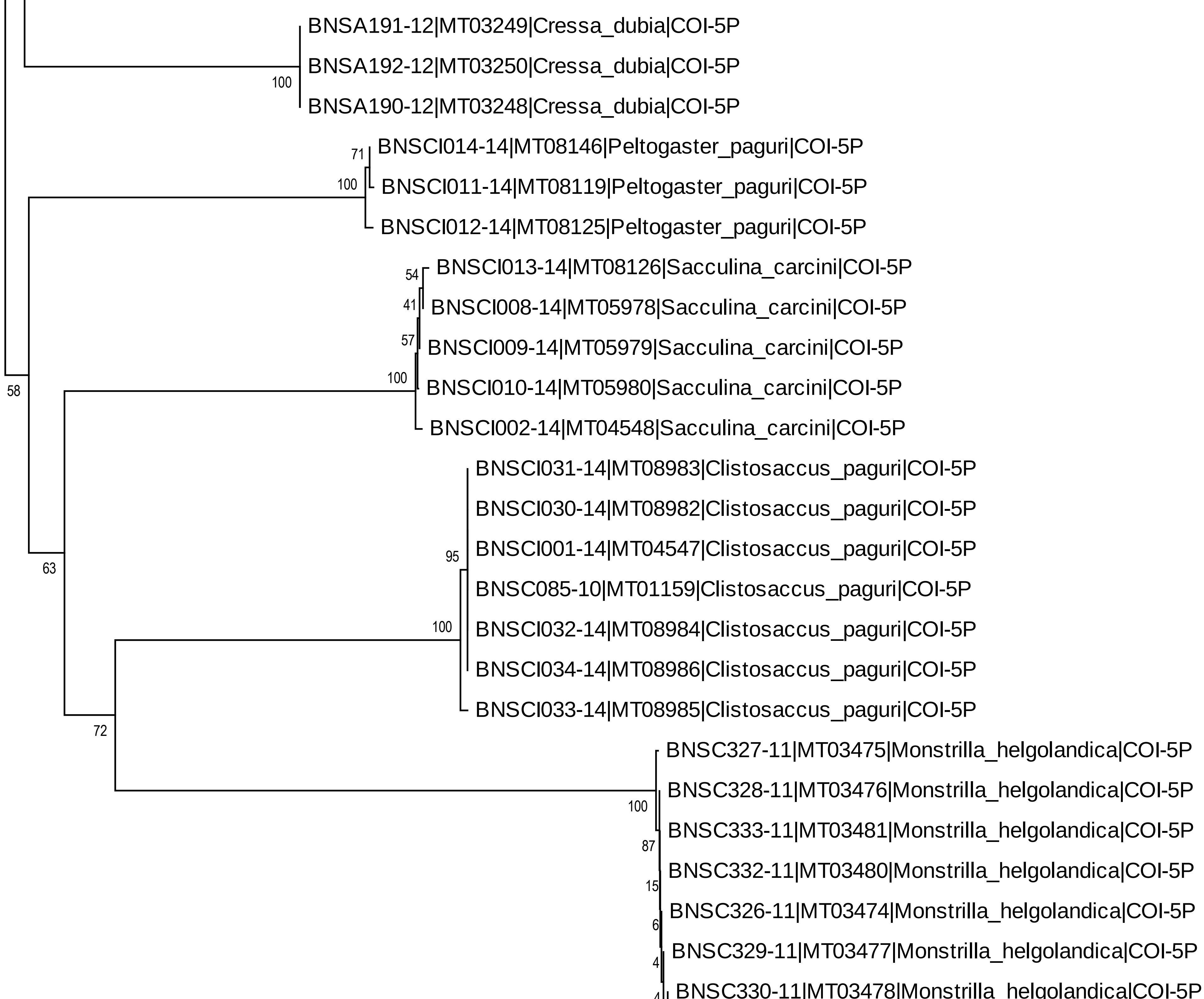
BNSA191-12|MT03249|Cressa_dubia|COI-5P
BNSA192-12|MT03250|Cressa_dubia|COI-5P
100
BNSA190-12|MT03248|Cressa_dubia|COI-5P
71
BNSCI014-14|MT08146|Peltogaster_paguri|COI-5P
100
BNSCI011-14|MT08119|Peltogaster_paguri|COI-5P
BNSCI012-14|MT08125|Peltogaster_paguri|COI-5P
54
BNSCI013-14|MT08126|Sacculina_carcini|COI-5P
41
BNSCI008-14|MT05978|Sacculina_carcini|COI-5P
57
BNSCI009-14|MT05979|Sacculina_carcini|COI-5P
100
BNSCI010-14|MT05980|Sacculina_carcini|COI-5P
58
BNSCI002-14|MT04548|Sacculina_carcini|COI-5P
BNSCI031-14|MT08983|Clistosaccus_paguri|COI-5P
BNSCI030-14|MT08982|Clistosaccus_paguri|COI-5P
BNSCI001-14|MT04547|Clistosaccus_paguri|COI-5P
95
63
BNSC085-10|MT01159|Clistosaccus_paguri|COI-5P
100
BNSCI032-14|MT08984|Clistosaccus_paguri|COI-5P
BNSCI034-14|MT08986|Clistosaccus_paguri|COI-5P
BNSCI033-14|MT08985|Clistosaccus_paguri|COI-5P
72
BNSC327-11|MT03475|Monstrilla_helgolandica|COI-5P
BNSC328-11|MT03476|Monstrilla_helgolandica|COI-5P
100
BNSC333-11|MT03481|Monstrilla_helgolandica|COI-5P
87
BNSC332-11|MT03480|Monstrilla_helgolandica|COI-5P
15
BNSC326-11|MT03474|Monstrilla_helgolandica|COI-5P
6
BNSC329-11|MT03477|Monstrilla_helgolandica|COI-5P
4
BNSC330-11|MT03478|Monstrilla_helgolandica|COI-5P

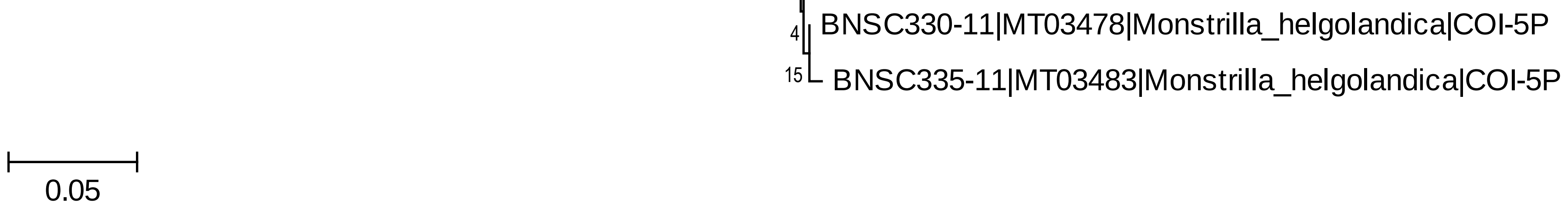
4
BNSC330-11|MT03478|Monstrilla_helgolandica|COI-5P
15
BNSC335-11|MT03483|Monstrilla_helgolandica|COI-5P
0.05
