# Supplementary material for: The Application of DNA Barcodes for the Identification of Marine Crustaceans from the North Sea and Adjacent Regions
Source: PLoS One. 2015 Sep 29;10(9):e0139421. doi: 10.1371/journal.pone.0139421 (PMC4587929; doi:10.1371/journal.pone.0139421)
Supplement: S1 Table — Divergence values were calculated for all studied sequences, using the Nearest Neighbor Summary implemented in the Barcode Gap Analysis tool provided by the Barcode of Life Data System (BOLD). Align sequencing option: BOLD aligner (amino acid based HMM), ambiguous base/gap handling: pairwise deletion. ISD = intraspecific distance. BINs are based on the barcode analysis from 02-06-2015. AphiaID codes were retrieved from the World Register of Marine Species (WoRMS; www.marinespecies.org) on 02-06-2015. Asterisks indicate new species to BOLD. (DOCX) [file pone.0139421.s004.docx]

| Species | WoRMS  AphiaID | BIN | *n* | Mean  ISD | Max  ISD | Nearest species (NS) | Distance  to NS |
| --- | --- | --- | --- | --- | --- | --- | --- |
| Branchiopoda: Phyllopoda: Diplostraca |  |  |  |  |  |  |  |
| *Evadne nordmanni* Lovén, 1836 | 106273 | ACF5469 | 1 | N.A. | N.A. | *Podon intermedius* | 18.64 |
| **Evadne spinifera* P.E. Müller, 1867 | 106274 | ACP7351 | 8 | 0.94 | 1.87 | *Podon intermedius* | 19.06 |
| *Penilia avirostris* Dana, 1849 | 106272 | AAC6757 | 1 | N.A. | N.A. | *Pisidia longicornis* | 26.98 |
| *Podon intermedius* Lilljeborg, 1853 | 106276 | AAD4610 | 5 | 0 | 0 | *Podon leuckartii* | 18.01 |
| *Podon leuckartii* (Sars G.O., 1862) | 106277 | AAD4606 | 9 | 0.03 | 0.15 | *Podon intermedius* | 18.01 |
| Maxillopoda: Thecostraca: Rhizocephala: Aktentrogonida |  |  |  |  |  |  |  |
| **Clistosaccus* *paguri* Lilljeborg, 1861 | 134786 | ACR4918 | 7 | 0.22 | 0.77 | *Ebalia tumefacta* | 37.28 |
| Maxillopoda: Thecostraca: Rhizocephala: Kentrogonida |  |  |  |  |  |  |  |
| **Peltogaster* *paguri* Rathke, 1842 | 134798 | ACR6158 | 3 | 0.51 | 0.77 | *Balanus balanus* | 31.34 |
| *Sacculina* *carcini* Thompson, 1836 | 134805 | AAC4415 | 5 | 0.31 | 0.62 | *Hyperia galba* | 34.75 |
| Maxillopoda: Thecostraca: Thoracica: Lepadiformes |  |  |  |  |  |  |  |
| **Dosima* *fascicularis* (Ellis & Solander, 1786) | 106148 | ACR5913 | 4 | 1.58 | 2.18 | *Verruca stroemia* | 20.31 |
| Maxillopoda: Thecostraca: Thoracica: Scalpelliformes |  |  |  |  |  |  |  |
| **Scalpellum* *scalpellum* (Linnaeus, 1767) | 106204 | ACR4968 | 6 | 0.52 | 0.77 | *Amphibalanus improvisus* | 20.3 |
| Maxillopoda: Thecostraca: Thoracica: Sessilia |  |  |  |  |  |  |  |
| *Amphibalanus* *improvisus* (Darwin, 1854) | 421139 | AAE2482 | 5 | 0.43 | 0.93 | *Balanus balanus* | 15.33 |
| *Austrominius* *modestus* (Darwin, 1854) | 712167 | ABX4245, ACR4768 | 10 | 1.39 | 3.81 | *Balanus balanus* | 17.1 |
| *Balanus* *balanus* (Linnaeus, 1758) | 106213 | AAB1410 | 10 | 0.32 | 0.77 | *Semibalanus balanoides* | 14.85 |
| **Balanus* *crenatus* Bruguière, 1789 | 106215 | AAY4996 | 15 | 0.74 | 1.89 | *Balanus* sp*.* | 14.35 |
| *Balanus* sp. | N.A. | AAG0069 | 3 | 0.93 | 1.24 | *Balanus crenatus* | 14.35 |
| *Semibalanus* *balanoides* (Linnaeus, 1767) | 106210 | AAA3935 | 18 | 0.59 | 1.73 | *Balanus balanus* | 14.85 |
| *Verruca stroemia* (Müller, 1776) | 106257 | ACH5756 | 6 | 0.44 | 0.62 | *Verruca stroemia* | 15.32 |
| Maxillopoda: Copepoda: Gymnoplea: Calanoida |  |  |  |  |  |  |  |
| *Acartia bifilosa* (Giesbrecht, 1881) | 104249 | ACM7252 | 7 | 0.25 | 0.77 | *Acartia tonsa* | 21.03 |
| *Acartia clausi* Giesbrecht, 1889 | 104251 | AAT9961 | 19 | 0.4 | 1.27 | *Acartia tonsa* | 20.53 |
| *Acartia tonsa* Dana, 1849 | 104262 | AAA5453 | 16 | 0.53 | 1.71 | *Acartia clausi* | 20.53 |
| *Anomalocera patersoni* Templeton, 1837 | 104722 | ACM8185, ACM8186 | 22 | 1.87 | 6.11 | *Eurytemora affinis* | 23.15 |
| *Calanus helgolandicus* (Claus, 1863) | 104466 | AAB3934 | 14 | 0.64 | 2.99 | *Paracalanus parvus* | 25.07 |
| *Centropages hamatus* (Lilljeborg, 1853) | 104496 | ACM7673 | 9 | 0.46 | 1.87 | *Centropages typicus* | 20.65 |
| *Centropages typicus* Krøyer, 1849 | 104499 | AAW6024 | 11 | 0.89 | 1.4 | *Centropages hamatus* | 20.65 |
| *Eurytemora affinis* (Poppe, 1880) | 104872 | AAA4925 | 10 | 0.24 | 0.77 | *Anomalocera patersoni* | 23.15 |
| *Isias claviceps* Boeck, 1865 | 104501 | ACM7740 | 5 | 0.43 | 0.61 | *Centropages hamatus* | 26.69 |
| *Paracalanus parvus* (Claus, 1863) | 104685 | AAU0467 | 9 | 0.29 | 0.62 | *Pseudocalanus elongatus* | 23.26 |
| *Pseudocalanus elongatus* (Boeck, 1865) | 104515 | AAF6145 | 9 | 1.93 | 4.43 | *Pseudocalanus moultoni* | 17.38 |
| *Pseudocalanus moultoni* Frost, 1989 | 157678 | AAH8137 | 5 | 0.46 | 0.81 | *Pseudocalanus elongatus* | 17.38 |
| *Pseudodiaptomus marinus* Sato, 1913 | 360352 | AAX6988 | 2 | 0.31 | 0.31 | *Pseudocalanus elongatus* | 25.5 |
| *Temora longicornis* (Müller O.F., 1785) | 104878 | AAO2762 | 19 | 0.48 | 2.66 | *Anomalocera patersoni* | 28.5 |
| Maxillopoda: Copepoda: Podoplea: Cyclopoida |  |  |  |  |  |  |  |
| **Chondracanthus lophii* Johnston, 1836 | 128763 | ACS7807 | 6 | 0.19 | 0.46 | *Chondrac. merluccii* | 23.81 |
| **Chondracanthus merluccii* (Holten, 1802) | 128764 | ACR5718 | 5 | 0.8 | 1.08 | *Chondracanthus lophii* | 23.81 |
| **Ditrichocorycaeus anglicus* (Lubbock, 1857) | 128805 | ABU9030 | 9 | 0.38 | 0.77 | *Evansula pygmaea* | 28.95 |
| *Oithona similis* Claus, 1866 | 106656 | AAG5172 | 2 | 0 | 0 | *Hemigrapsus takanoi* | 34.24 |
| Maxillopoda: Copepoda: Podoplea: Harpacticoida |  |  |  |  |  |  |  |
| **Asellopsis intermedia* (Scott T., 1895) | 116217 | ACR6121 | 3 | 0 | 0 | *Evansula pygmaea* | 22.31 |
| *Delavalia palustris* Brady, 1868 | 350706 | ACS7374 | 1 | N.A. | N.A. | *Asellopsis intermedia* | 35.13 |
| **Enhydrosoma gariene* Gurney, 1930 | 115780 | ACS8050 | 2 | 1.23 | 1.23 | *Evansula pygmaea* | 28.17 |
| *Euterpina acutifrons* (Dana, 1847) | 116162 | ACQ8377 | 4 | 0 | 0 | *Tachidius discipes* | 37.31 |
| **Evansula pygmaea* (Scott T., 1903) | 115815 | ACR6237 | 1 | N.A. | N.A. | *Asellopsis intermedia* | 22.31 |
| **Harpacticus flexus* Brady & Robertson D., 1873 | 116168 | ACS7639 | 5 | 0.25 | 0.61 | *Evansula pygmaea* | 28.96 |
| **Pseudobradya attenuata* Sars G.O., 1920 | 116125 | ACR5921 | 2 | 0.32 | 0.32 | *Pseudobradya minor* | 21.42 |
| **Pseudobradya minor* Scott T. & A., 1895 | 116140 | ACS7853 | 4 | 0.42 | 0.83 | *Pseudobradya attenuata* | 21.42 |
| **Tachidius discipes* Giesbrecht, 1881 | 157725 | ACR6189 | 4 | 1.15 | 2.1 | *Euterpina acutifrons* | 37.31 |
| **Thalestris longimana* Claus, 1863 | 116619 | ABU8720 | 5 | 0.84 | 1.42 | *Evansula pygmaea* | 25.36 |
| Maxillopoda: Copepoda: Podoplea: Siphonostomatoida |  |  |  |  |  |  |  |
| **Caligus curtus* O. F. Müller, 1785 | 135749 | AAW8408 | 2 | 1.47 | 1.47 | *Caligus elongatus* | 22.43 |
| **Caligus elongatus* Nordmann, 1832 | 135754 | AAE8403, AAE8404 | 10 | 6.9 | 14.87 | *Caligus curtus* | 22.43 |
| **Clavella adunca* (Strøm, 1762) | 135842 | ACR5924 | 6 | 1.02 | 1.54 | *Parabrachiella merluccii* | 29.43 |
| **Lernaeenicus sprattae* (Sowerby, 1806) | 135991 | AAU6385 | 7 | 1.09 | 1.56 | *Caligus elongatus* | 30.64 |
| **Parabrachiella merluccii* (Bassett-Smith, 1896) | 478458 | ACR6106 | 1 | N.A. | N.A. | *Clavella adunca* | 29.43 |
| Maxillopoda: Copepoda: Podoplea: Monstrilloida |  |  |  |  |  |  |  |
| *Monstrilla helgolandica* Claus, 1863 | 119802 | ABU8379 | 8 | 0.08 | 0.31 | *Neomysis americana* | 44.38 |
| Malacostraca: Hoplocarida: Stomatopoda |  |  |  |  |  |  |  |
| **Rissoides desmaresti* (Risso, 1816) | 136135 | ACP8957 | 1 | N.A. | N.A. | *Crangon crangon* | 20.38 |
| Malacostraca: Eumalacostraca: Peracarida: Mysida |  |  |  |  |  |  |  |
| *Mesopodopsis slabberi* (Van Beneden, 1861) | 120072 | ABX4244 | 5 | 1.15 | 1.87 | *Schistomysis kervillei* | 27.6 |
| **Neomysis americana* (S.I. Smith, 1873) | 157807 | ACG4579 | 5 | 0.52 | 0.77 | *Eualus cranchii* | 24.17 |
| *Praunus flexuosus* (Müller, 1776) | 120177 | AAH7607 | 12 | 0.5 | 0.94 | *Praunus inermis* | 26.01 |
| **Praunus inermis* (Rathke, 1843) | 120178 | ACP7467 | 1 | N.A. | N.A. | *Praunus flexuosus* | 26.01 |
| **Schistomysis kervillei* (Sars G.O., 1865) | 120203 | ABX4215 | 5 | 0.18 | 0.46 | *Schistomysis ornata* | 23.33 |
| **Schistomysis ornata* (Sars G.O., 1864) | 120204 | ACP8186 | 1 | N.A. | N.A. | *Schistomysis kervillei* | 23.33 |
| **Siriella armata* (Milne Edwards, 1837) | 120208 | ABX2409 | 6 | 1.16 | 1.71 | *Idotea balthica* | 28.35 |
| Malacostraca: Eumalacostraca: Peracarida: Amphipoda |  |  |  |  |  |  |  |
| **Ampelisca diadema* (Costa, 1853) | 101896 | ABW2163 | 8 | 0.2 | 0.62 | *Ampelisca tenuicornis* | 20.07 |
| **Ampelisca spinipes* Boeck, 1861 | 101928 | ABW4667 | 1 | N.A. | N.A. | *Ampelisca tenuicornis* | 20.51 |
| *Ampelisca tenuicornis* Lilljeborg, 1855 | 101930 | AAJ2286 | 1 | N.A | N.A. | *Ampeliscas diadema* | 20.07 |
| *Amphilochus manudens* Bate, 1862 | 101967 | ABX4251 | 3 | 0 | 0 | *Maera loveni* | 36.54 |
| **Aora gracilis* (Bate, 1857) | 102012 | ABW1982 | 14 | 0.88 | 1.71 | *Jassa falcata* | 21.52 |
| **Apherusa bispinosa* (Bate, 1857) | 102160 | AAU3746 | 3 | 0.1 | 0.16 | *Apherusa jurinei* | 24.81 |
| *Apherusa jurinei* Milne Edwards, 1830 | 102168 | AAY5341 | 6 | 0.21 | 0.46 | *Orchestria mediterranea* | 21.42 |
| *Apohyale prevostii* (Milne Edwards, 1830) | 490616 | AAB5862 | 12 | 0.14 | 0.33 | *Gammarus salinus* | 19.37 |
| **Bathyporeia pelagica* (Bate, 1856) | 103066 | ABW4326 | 4 | 0 | 0 | *Bathyporeia pilosa* | 19.99 |
| **Bathyporeia pilosa* Lindström, 1855 | 103068 | ABW2047 | 8 | 0.08 | 0.31 | *Bathyporeia sarsi* | 16.89 |
| **Bathyporeia sarsi* Watkin, 1938 | 103073 | ACG7768 | 5 | 0.12 | 0.31 | *Bathyporeia pilosa* | 16.89 |
| **Bathyporeia* sp. | N.A. | ACG9200 | 2 | 0 | 0 | *Bathyporeia pilosa* | 22.31 |
| *Calliopius laeviusculus* (Krøyer, 1838) | 102178 | AAM7244 | 1 | N.A | N.A. | *Gammarellus homari* | 27.35 |
| *Caprella mutica* Schurin, 1935 | 146768 | AAE7686 | 9 | 0.24 | 1.09 | *Jassa marmorata* | 22.82 |
| *Cheirocratus sundevalli* (Rathke, 1843) | 102798 | ABW2107 | 6 | 0.15 | 0.46 | *Jassa marmorata* | 21.03 |
| *Corophium volutator* (Pallas, 1766) | 102101 | AAE9752 | 4 | 0 | 0 | *Monocorophium sextonae* | 22.51 |
| **Cressa dubia* (Bate, 1857) | 102119 | ABW4724 | 3 | 0 | 0 | *Jassa marmorata* | 27.72 |
| **Deshayesorchestia deshayesii* (Audouin, 1826) | 236548 | ABW1937 | 2 | 0.61 | 0.61 | *Orchestria mediterranea* | 19.46 |
| **Dexamine spinosa* (Montagu, 1813) | 102135 | AAV9173 | 1 | N.A | N.A. | *Orchestria mediterranea* | 26.33 |
| **Dexamine thea* Boeck, 1861 | 102136 | AAG7227 | 5 | 0.28 | 0.46 | *Dexamine spinosa* | 27.93 |
| **Dulichia falcata* (Bate, 1857) | 103031 | ABW2232 | 4 | 0.08 | 0.15 | *Orchestria mediterranea* | 24.21 |
| *Echinogammarus marinus* (Leach, 1815) | 102261 | AAC5398 | 17 | 0.23 | 0.65 | *Epimeria cornigera* | 29.62 |
| **Epimeria cornigera* (Fabricius, 1779) | 102145 | AAU2062 | 5 | 0.09 | 0.15 | *Gammarellus homari* | 24.86 |
| **Ericthonius punctatus* (Bate, 1857) | 102408 | ABW2100 | 12 | 0.03 | 0.15 | *Apohyale prevostii* | 20.78 |
| **Gammarella fucicola* (Leach, 1814) | 102811 | ACP7515 | 3 | 0.1 | 0.15 | *Gammaropsis nitida* | 23.63 |
| *Gammarellus angulosus* (Rathke, 1843) | 102251 | AAL3037 | 5 | 0.06 | 0.16 | *Gammarellus homari* | 11.35 |
| **Gammarellus homari* (Fabricius, 1779) | 102253 | ACP9414 | 9 | 0.07 | 0.31 | *Gammarellus angulosus* | 11.35 |
| **Gammaropsis nitida* (Stimpson, 1853) | 102367 | ACP9777 | 9 | 0.36 | 0.96 | *Ericthonius punctatus* | 20.8 |
| *Gammarus crinicornis* Stock, 1966 | 102275 | AAF5354 | 2 | 1.39 | 1.39 | *Gammarus salinus* | 22.82 |
| *Gammarus duebeni* Lilljeborg, 1852 | 102276 | AAA5117 | 1 | N.A | N.A. | *Gammarus salinus* | 24.62 |
| *Gammarus locusta* (Linnaeus, 1758) | 102281 | AAC0402 | 6 | 0.6 | 1.08 | *Gammarus crinicornis* | 22.96 |
| *Gammarus salinus* Spooner, 1947 | 102292 | ACG9079, AAB7068,  ACG8870 | 6 | 2.01 | 4.14 | *Gammarellus angulosus* | 18.85 |
| **Gitana sarsi* Boeck, 1871 | 101977 | ABX4219 | 4 | 0.16 | 0.31 | *Ebalia cranchii* | 28.64 |
| **Harpinia antennaria* Meinert, 1890 | 102960 | ABX4454 | 1 | N.A | N.A. | *Orchestria mediterranea* | 32.59 |
| **Haustorius arenarius* (Slabber, 1769) | 102317 | ACG9283 | 1 | N.A | N.A. | *Eualus cranchii* | 28.09 |
| *Hyperia galba* (Montagu, 1815) | 103251 | AAE5954 | 5 | 0.06 | 0.15 | *Jassa marmorata* | 30.13 |
| *Jassa falcata* (Montagu, 1808) | 102431 | AAU2334 | 5 | 0.42 | 0.78 | *Jassa marmorata* | 20.48 |
| *Jassa herdmani* (Walker, 1893) | 102432 | AAX8442 | 6 | 1.33 | 2.18 | *Jassa marmorata* | 20.86 |
| *Jassa marmorata* Holmes, 1905 | 102433 | AAA3311 | 15 | 0.02 | 0.16 | *Jassa falcata* | 20.48 |
| **Jassa pusilla* (Sars, 1894) | 102437 | ACP9511 | 2 | 1.02 | 1.02 | *Jassa marmorata* | 20.63 |
| **Maera loveni* (Bruzelius, 1859) | 102820 | ABX4234 | 1 | N.A | N.A. | *Cheirocratus sundevalli* | 31.87 |
| **Megamphopus cornutus* Norman, 1869 | 102377 | ABW4956 | 3 | 0.51 | 0.62 | *Gammaropsis nitida* | 21.6 |
| *Melita palmata* (Montagu, 1804) | 102843 | AAY5475 | 4 | 0.08 | 0.15 | *Apohyale prevostii* | 35.42 |
| **Metopa alderi* (Bate, 1857) | 103116 | ACP7788 | 2 | 1.71 | 1.71 | *Stenula rubrovittata* | 31.34 |
| **Microprotopus maculatus* Norman, 1867 | 102380 | ABW2067 | 8 | 0.66 | 1.24 | *Ericthonius puctatus* | 26.03 |
| *Monocorophium acherusicum* (Costa, 1853) | 225814 | AAU1637 | 6 | 0.17 | 0.32 | *Monocorophium insidiosum* | 18.89 |
| *Monocorophium insidiosum* (Crawford, 1937) | 148592 | AAE1628, AAE9749 | 9 | 1.24 | 3.41 | *Monocorophium sextonae* | 18.7 |
| *Monocorophium sextonae* (Crawford, 1937) | 148603 | AAU1797 | 5 | 0 | 0 | *Monocorophium insidiosum* | 18.7 |
| *Orchestia gammarellus* (Pallas, 1766) | 103202 | AAA2088 | 3 | 0.1 | 0.15 | *Orchestria mediterranea* | 20.62 |
| *Orchestia mediterranea* Costa, 1853 | 103208 | AAC8222 | 7 | 0 | 0 | *Deshayesorch. deshayesii* | 19.46 |
| **Photis longicaudata* (Bate & Westwood, 1862) | 102383 | ACG9506 | 6 | 0.98 | 2.36 | *Ericthonius punctatus* | 25.4 |
| **Phtisica marina* Slabber, 1769 | 101864 | ABW2045 | 2 | 0.62 | 0.62 | *Caprella mutica* | 24.78 |
| **Scopelocheirus hopei* (Costa, 1851) | 102720 | ABX4447 | 1 | N.A | N.A. | *Bathyporeia pilosa* | 20.58 |
| **Stenothoe marina* (Bate, 1856) | 103166 | ABW3806 | 2 | 0.15 | 0.15 | *Apohyale prevostii* | 29.23 |
| **Stenothoe monoculoides* (Montagu, 1815) | 103169 | ABW4652 | 4 | 0 | 0 | *Orchestria mediterranea* | 33.56 |
| **Stenula rubrovittata* (Sars, 1882) | 103178 | ACP8131 | 4 | 0.08 | 0.15 | *Metopa alderi* | 31.34 |
| *Talitrus saltator* (Montagu, 1808) | 103220 | AAC4712 | 9 | 0 | 0 | *Apohyale prevostii* | 19.89 |
| **Urothoe elegans* (Bate, 1857) | 103228 | ABX4246 | 3 | 0 | 0 | *Urothoe poseidonis* | 20.62 |
| *Urothoe poseidonis* Reibish, 1905 | 103235 | ABW2004 | 10 | 0.03 | 0.15 | *Urothoe elegans* | 20.62 |
| Malacostraca: Eumalacostraca: Peracarida: Isopoda |  |  |  |  |  |  |  |
| **Astacilla intermedia* (Goodsir, 1841) | 119023 | ACP7495, ACP7496 | 3 | 3.19 | 4.79 | *Astacilla longicornis* | 20.6 |
| **Astacilla longicornis* (Sowerby, 1806) | 119024 | ACP9543 | 10 | 0.19 | 0.61 | *Astacilla intermedia* | 20.6 |
| **Athelges paguri* (Rathke, 1843) | 118196 | ACS7386 | 3 | 1.03 | 1.4 | *Macropodia tenuirostris* | 25.67 |
| *Eurydice pulchra* Leach, 1815 | 118852 | ACH9769 | 8 | 0.15 | 0.61 | *Idotea balthica* | 24.49 |
| *Idotea balthica* (Pallas, 1772) | 119039 | AAA8398 | 6 | 0.5 | 0.89 | *Idotea emarginata* | 15.08 |
| **Idotea emarginata* (Fabricius, 1793) | 119043 | AAU0407 | 3 | 0.94 | 1.26 | *Idotea balthica* | 15.08 |
| *Idotea granulosa* Rathke, 1843 | 119044 | AAM7896 | 9 | 0.16 | 0.32 | *Idotea pelagica* | 12.01 |
| **Idotea linearis* (Linnaeus, 1766) | 119046 | ACP8008 | 9 | 0.17 | 0.31 | *Idotea balthica* | 19.92 |
| **Idotea metallica* Bosc, 1802 | 119047 | ACP4947 | 2 | 0.17 | 0.17 | *Idotea emarginata* | 17.49 |
| *Idotea pelagica* Leach, 1815 | 119050 | AAO1731 | 3 | 0 | 0 | *Idotea granulosa* | 12.01 |
| *Jaera albifrons* Leach, 1814 | 118715 | AAI7930 | 8 | 0.97 | 1.71 | *Eualus cranchii* | 26.56 |
| **Janira maculosa* Leach, 1814 | 118732 | AAU1507 | 6 | 0 | 0 | *Ebalia tuberosa* | 27.68 |
| **Lekanesphaera rugicauda* (Leach, 1814) | 118958 | ACP7370 | 5 | 0.06 | 0.15 | *Idotea pelagica* | 24.48 |
| *Ligia oceanica* (Linnaeus, 1767) | 146999 | AAJ2795 | 5 | 0.09 | 0.15 | *Idotea granulosa* | 27.17 |
| *Natatolana borealis* (Lilljeborg, 1851) | 118859 | ACR3559 | 3 | 0 | 0 | *Natatolana gallica* | 15.82 |
| **Natatolana gallica* (Hansen, 1905) | 118861 | ACP8030 | 1 | N.A. | N.A. | *Natatolana borealis* | 15.82 |
| Malacostraca: Eumalacostraca: Peracarida: Cumacea |  |  |  |  |  |  |  |
| **Bodotria scorpioides* (Montagu, 1804) | 110445 | ABW6667 | 9 | 0.31 | 0.62 | *Munida rugosa* | 29.92 |
| *Diastylis rathkei* (Krøyer, 1841) | 110487 | AAI9440 | 1 | N.A. | N.A. | *Goneplax rhomboides* | 26 |
| **Diastylis rugosa* Sars G.O., 1865 | 110488 | AAV9179 | 1 | N.A. | N.A. | *Diastylis rathkei* | 29.73 |
| **Monopseudocuma gilsoni* (Gilson, 1906) | 422916 | AAU1761 | 3 | 0.52 | 0.62 | *Idotea pelagica* | 28.28 |
| Malacostraca: Eumalacostraca: Eucarida: Euphausiacea |  |  |  |  |  |  |  |
| *Meganyctiphanes norvegica* (Sars M., 1857) | 110690 | AAB7620 | 4 | 0.33 | 0.62 | *Pagurus prideaux* | 18.34 |
| Malacostraca: Eumalacostraca: Eucarida: Decapoda |  |  |  |  |  |  |  |
| *Anapagurus laevis* (Bell, 1846) | 107218 | ABX3753 | 2 | 0.92 | 0.92 | *Lithodes maja* | 17.11 |
| *Atelecyclus rotundatus* (Olivi, 1792) | 107273 | AAE4444 | 7 | 0.53 | 0.92 | *Cancer pagurus* | 17.2 |
| **Axius stirynchus* Leach, 1815 | 477515 | ACN5342 | 1 | N.A. | N.A. | *Galathea intermedia* | 26.12 |
| *Callianassa subterranea* (Montagu, 1808) | 107729 | ABX4223 | 5 | 0.68 | 0.99 | *Diogenes pugilator* | 22.22 |
| *Cancer pagurus* Linnaeus, 1758 | 107276 | AAF8058 | 14 | 0.07 | 0.32 | *Atelecyclus rotundatus* | 17.2 |
| *Carcinus maenas* (Linnaeus, 1758) | 107381 | AAA7687 | 11 | 0.64 | 1.24 | *Liocarcinus navigator* | 19.45 |
| *Corystes cassivelaunus* (Pennant, 1777) | 107277 | AAF0742 | 8 | 0.08 | 0.31 | *Cancer pagurus* | 20.04 |
| **Crangon allmanni* Kinahan, 1860 | 107551 | AAY7692 | 10 | 0.03 | 0.15 | *Crangon crangon* | 10.85 |
| **Crangon crangon* (Linnaeus, 1758) | 107552 | AAU2652 | 4 | 0.34 | 0.68 | *Crangon allmanni* | 10.85 |
| **Diogenes pugilator* (Roux, 1829) | 107199 | AAX9663 | 9 | 0.12 | 0.35 | *Munida rugosa* | 17.95 |
| *Ebalia cranchii* Leach, 1817 | 107294 | AAY0490 | 17 | 0.25 | 1.71 | *Ebalia tumefacta* | 6.14 |
| **Ebalia tuberosa* (Pennant, 1777) | 107301 | ACN6436 | 5 | 0.28 | 0.46 | *Ebalia cranchii* | 15.43 |
| *Ebalia tumefacta* (Montagu, 1808) | 107302 | ABX7355 | 5 | 0.15 | 0.31 | *Ebalia cranchii* | 6.14 |
| *Eriocheir sinensis* Milne Edwards, 1853 | 107451 | AAB0750, AAA8754 | 5 | 2.06 | 4.76 | *Hemigrapsus takanoi* | 16.05 |
| *Eualus cranchii* (Leach, 1817) | 156083 | ABW0161 | 2 | 0.46 | 0.46 | *Munida rugosa* | 17.66 |
| *Eualus pusiolus* (Krøyer, 1841) | 107507 | AAF3716 | 3 | 0.51 | 0.77 | *Eualus* sp. | 11.95 |
| **Eualus* sp. | N.A. | ABX4202 | 1 | N.A. | N.A. | *Eualus pusiolus* | 11.95 |
| *Eurynome aspera* (Pennant, 1777) | 107318 | ABV4962 | 2 | 0.93 | 0.93 | *Goneplax rhomboides* | 21.18 |
| *Galathea* *dispersa* Bate, 1859 | 107148 | AAL3139 | 10 | 0.87 | 2.19 | *Galathea intermedia* | 15.35 |
| *Galathea* *intermedia* Lilljeborg, 1851 | 107150 | AAV6847 | 18 | 0.13 | 0.64 | *Galathea dispersa* | 15.35 |
| *Goneplax* *rhomboides* (Linnaeus, 1758) | 107292 | AAB5890 | 6 | 0 | 0 | *Ebalia tumefacta* | 15.87 |
| *Hemigrapsus* *sanguineus* (De Haan, 1835) | 158417 | AAB4163 | 10 | 0.31 | 0.77 | *Hemigrapsus takanoi* | 12.97 |
| *Hemigrapsus* *takanoi* Asakura & Watanabe, 2005 | 389288 | AAY5062 | 14 | 0.28 | 0.78 | *Hemigrapsus sanguineus* | 12.97 |
| *Hippolyte* *varians* Leach, 1814 | 107518 | ABV3915 | 1 | N.A. | N.A. | *Eualus cranchii* | 24.6 |
| *Homarus* *gammarus* (Linnaeus, 1758) | 107253 | AAK5475 | 3 | 0 | 0 | *Nephrops norvegicus* | 13.86 |
| *Hyas* *araneus* (Linnaeus, 1758) | 107322 | AAC2751 | 11 | 0.63 | 1.36 | *Hyas coarctatus* | 2.36 |
| *Hyas* *coarctatus* Leach, 1816 | 107323 | AAA9922 | 10 | 0.06 | 0.31 | *Hyas araneus* | 2.36 |
| *Inachus* *dorsettensis* (Pennant, 1777) | 107327 | ABX4182 | 3 | 0.41 | 0.62 | *Macropodia prava* | 14.42 |
| *Liocarcinus* *depurator* (Linnaeus, 1758) | 107387 | AAC6700 | 22 | 0.6 | 1.25 | *Liocarcinus marmoreus* | 13.25 |
| *Liocarcinus* *holsatus* (Fabricius, 1798) | 107388 | AAC6074 | 11 | 0.17 | 0.49 | *Liocarcinus marmoreus* | 15.6 |
| *Liocarcinus* *marmoreus* (Leach, 1814) | 107390 | AAX5076 | 5 | 0.25 | 0.46 | *Liocarcinus depurator* | 13.25 |
| *Liocarcinus* *navigator* (Herbst, 1794) | 107392 | AAX5074 | 5 | 0.06 | 0.15 | *Liocarcinus pusillus* | 11.02 |
| *Liocarcinus* *pusillus* (Leach, 1816) | 107393 | ABX7419 | 2 | 0 | 0 | *Liocarcinus navigator* | 11.02 |
| *Lithodes* *maja* (Linnaeus, 1758) | 107205 | AAD4025 | 17 | 0.31 | 0.94 | *Pagurus alatus* | 15.59 |
| **Macropodia* *parva* Van Noort & Adema, 1985 | 107344 | AAF8277 | 9 | 0.58 | 1.12 | *Macropodia rostrata* | 0 |
| *Macropodia* *rostrata* (Linnaeus, 1761) | 107345 | AAF8277 | 7 | 0.48 | 0.77 | *Macropodia parva* | 0 |
| *Macropodia* *tenuirostris* (Leach, 1814) | 107346 | AAD5026 | 20 | 0.19 | 0.62 | *Marcopodia parva* | 4.32 |
| *Monodaeus* *couchi* (Couch, 1851) | 241154 | AAB4955 | 1 | N.A. | N.A. | *Hyas coarctatus* | 15.13 |
| *Munida* *rugosa* (Fabricius, 1775) | 107160 | AAX3135 | 1 | N.A. | N.A. | *Upogebia deltaura* | 17.31 |
| *Necora* *puber* (Linnaeus, 1767) | 107398 | AAB0797 | 1 | N.A. | N.A. | *Liocarcinus pusillus* | 16.8 |
| *Nephrops* *norvegicus* (Linnaeus, 1758) | 107254 | AAD0739 | 12 | 0.54 | 1.45 | *Homarus gammarus* | 13.86 |
| *Pagurus* *alatus* Fabricius, 1775 | 107230 | ACP5591 | 4 | 0 | 0 | *Pagurus prideaux* | 13.53 |
| *Pagurus* *bernhardus* (Linnaeus, 1758) | 107232 | AAC3652 | 8 | 0.76 | 1.67 | *Pagurus pubescens* | 11.69 |
| *Pagurus* *prideaux* Leach, 1815 | 107239 | AAB0339 | 13 | 0.48 | 1.33 | *Pagurus alatus* | 13.53 |
| *Pagurus* *pubescens* Krøyer, 1838 | 107240 | AAB6221 | 12 | 1.6 | 2.89 | *Pagurus bernhardus* | 11.69 |
| *Palaemon* *adspersus* Rathke, 1837 | 107613 | AAX1913 | 5 | 0.25 | 0.46 | *Palaemon longirostris* | 12.61 |
| *Palaemon* *elegans* Rathke, 1837 | 107614 | AAC8513 | 5 | 0.55 | 1.24 | *Palaemon adspersus* | 20.08 |
| *Palaemon* *longirostris* Milne Edwards, 1837 | 107615 | AAC6996 | 5 | 0.17 | 0.46 | *Palaemon adspersus* | 12.61 |
| *Palaemon* *macrodactylus* Rathbun, 1902 | 181372 | ACG4332 | 8 | 0.13 | 0.31 | *Palaemon longirostris* | 19.93 |
| *Palaemon* *serratus* (Pennant, 1777) | 107616 | AAE4224 | 2 | 0.15 | 0.15 | *Palaemon adspersus* | 14.36 |
| *Palaemon* *varians* Leach, 1813 | 587704 | ACH4587 | 6 | 0.1 | 0.31 | *Palaemon adspersus* | 21.94 |
| *Pandalina* *brevirostris* (Rathke, 1843) | 107647 | ABV9748 | 8 | 0.04 | 0.16 | *Eualus cranchii* | 20.81 |
| *Pandalus* *montagui* Leach, 1814 | 107651 | AAB2199 | 21 | 1.49 | 4 | *Eualus cranchii* | 20.08 |
| *Philocheras* *bispinosus* (Hailstone, 1835) | 107557 | AAU2693 | 15 | 0.02 | 0.17 | *Philocheras trispinosus* | 14.97 |
| **Philocheras* *sculptus* (Bell, 1847) | 107561 | ACN3706 | 7 | 0.44 | 1.55 | *Philocheras bispinosus* | 21.25 |
| **Philocheras* *trispinosus* (Hailstone, 1835) | 107562 | ACN4156 | 2 | 0.15 | 0.15 | *Philocheras bispinosus* | 14.97 |
| *Pilumnus* *hirtellus* (Linnaeus, 1761) | 107418 | AAF8101 | 13 | 0.04 | 0.15 | *Goneplax rhomboides* | 19.03 |
| **Pinnotheres* *pisum* (Linnaeus, 1767) | 107473 | ABX7503 | 5 | 0 | 0 | *Liocarcinus pusillus* | 18.55 |
| *Pisa* *armata* (Latreille, 1803) | 107353 | ABV5409 | 2 | 0.31 | 0.31 | *Ebalia tuberosa* | 16.93 |
| *Pisidia* *longicornis* (Linnaeus, 1767) | 107188 | AAE3057 | 32 | 0.5 | 1.41 | *Liocarcinus navigator* | 20.44 |
| *Pontophilus* *spinosus* (Leach, 1816) | 107564 | ABU8850 | 4 | 0.15 | 0.31 | *Eualus cranchii* | 22.67 |
| **Processa* *modica* Williamson, 1979 | 107688 | ACN5555 | 6 | 0.1 | 0.31 | *Processa nouveli holthuisi* | 17.99 |
| *Processa* *nouveli* *holthuisi* Al-Adhub & Williamson, 1975 | 108344 | AAY7530 | 11 | 0.06 | 0.31 | *Processa modica* | 17.99 |
| *Spirontocaris* *liljeborgii* (Danielssen, 1859) | 107531 | AAU0406 | 13 | 0.24 | 0.62 | *Eualus pusiolus* | 20.56 |
| **Thia* *scutellata* (Fabricius, 1793) | 107281 | ACF2724 | 3 | 0.51 | 0.77 | *Liocarcinus pusillus* | 17.46 |
| **Upogebia* *deltaura* (Leach, 1815) | 107739 | ABX4222 | 9 | 0.38 | 0.77 | *Pagurus alatus* | 15.56 |
